# Predicting phenotypic traits of prokaryotes from protein domain frequencies

**DOI:** 10.1186/1471-2105-11-481

**Published:** 2010-09-24

**Authors:** Thomas Lingner, Stefanie Mühlhausen, Toni Gabaldón, Cedric Notredame, Peter Meinicke

**Affiliations:** 1 Department of Bioinformatics, Institute of Microbiology and Genetics, Georg-August-University Göttingen, Germany; 2 Bioinformatics Program, Centre for Genomic Regulation (CRG), UPF, Barcelona, Spain

## Abstract

**Background:**

Establishing the relationship between an organism's genome sequence and its phenotype is a fundamental challenge that remains largely unsolved. Accurately predicting microbial phenotypes solely based on genomic features will allow us to infer relevant phenotypic characteristics when the availability of a genome sequence precedes experimental characterization, a scenario that is favored by the advent of novel high-throughput and single cell sequencing techniques.

**Results:**

We present a novel approach to predict the phenotype of prokaryotes directly from their protein domain frequencies. Our discriminative machine learning approach provides high prediction accuracy of relevant phenotypes such as motility, oxygen requirement or spore formation. Moreover, the set of discriminative domains provides biological insight into the underlying phenotype-genotype relationship and enables deriving hypotheses on the possible functions of uncharacterized domains.

**Conclusions:**

Fast and accurate prediction of microbial phenotypes based on genomic protein domain content is feasible and has the potential to provide novel biological insights. First results of a systematic check for annotation errors indicate that our approach may also be applied to semi-automatic correction and completion of the existing phenotype annotation.

## Background

Despite initial expectations that the elucidation of the complete genome of an organism would enable understanding its biology, the establishment of specific links between genotype and phenotype remains one of the major challenges that biology faces today. In particular, this applies to complex phenotypes that depend on the effect of many genes. The identification of phenotype-specific genes or other genomic features opens the way to (1) formulate testable hypotheses on how the action of these genes may explain the occurrence of that phenotype and (2) predict the occurrence of that phenotype from the analysis of genomic sequences. Especially, the inference of microbial phenotypes on the basis of genomic features is highly relevant within the context of a growing number of (meta)genomic projects. Despite the progress that has been achieved for the investigation of phenotype-specific groups of genes, no practical solution exists for the genome-based prediction of phenotypical properties of prokaryotes.

The association of phenotypic and genotypic traits has been intensively investigated in the field of comparative genomics, mostly by exploiting the fact that organisms that share a particular phenotype are expected to share the set of genes responsible for that trait. In particular, *phylogenetic profiles *- presence/absence patterns of a given gene in a set of genomes - have been used to identify the function of uncharacterized proteins based on their co-occurrence with known proteins (e.g. [1], for an overview see [2]). On the other hand, the use of gene co-occurrence patterns is highly affected by missing data resulting from genome misannotation or erroneous assignment of orthology [2]. Since the orthology assignment step is a pivotal element of most phylogenomics approaches, the results of such gene-based methods can easily be deteriorated by simple genomic rearrangements such as gene fusion/fission events or domain shuffling [3]. Rather than correlating gene occurrence patterns only, several alternative approaches explicitly analyze genotype-phenotype associations by linking genes to a particular phenotype, e.g. Gram stain, oxygen requirement, endospore formation or motility [4-8]. In order to circumvent the orthology search step and the problem of incomplete presence/absence patterns, most of these approaches map genes and proteins to a more generic level such as clusters of orthologous genes [4,7], protein domains [5] and metabolic pathways [5,8].

A natural extension to these approaches is the *de novo *prediction of an organism's phenotype from the information contained in the genome. Such a predictive approach is likely to be increasingly useful in the context of rapid generation of genomic sequences from a growing number of microorganisms. For instance, current sequencing techniques allow the determination of genomic sequences of unculturable organisms as well as the rapid generation of sequences from recently isolated strains and even from single cells [9,10]. In particular, the rapidly growing number and sequencing depth of metagenomes from different natural environments and human body sites will allow the analysis of the genetic potential of the most dominant species without isolation of these organisms [11,12]. In many of the abovementioned cases, the availability of the genomic sequence will precede the standard phenotypic characterization by means of experimental tests.

To our knowledge, the possibility of directly predicting phenotypic properties from genome content has only been investigated in the context of assessing the predictive power of phenotype-associated genotypic features. For instance, in Kastenmüller et al. [8] genome-specific metabolic pathways have been evaluated for phenotype prediction using an intermediate mapping of proteins to pathways via EC categories together with multi-variate machine learning techniques. However, as the authors point out, the pathway inference method has several limitations arising from incomplete EC annotations for proteins and the limited metabolic knowledge represented in pathway databases. Furthermore, the approach is limited to pathways that have been detected and described in culturable species.

Here we present an approach for the prediction of microbial phenotypes that is entirely based on discriminative learning from protein domain frequencies as obtained from a large number of prokaryotic genomes. This approach does not require the identification of orthologous genes and fully operates on local genome features, i.e. the presence or absence of certain Pfam [13] domain families. The latter property in particular allows to make predictions in very early stages of a genome project, just after a sufficient sequencing coverage has been obtained. Provided the sequencing reads are long enough, e.g. like in recent versions of 454 technology and upcoming Solexa paired-end modules, this status can already be achieved before assembly of the reads into longer contigs. Thanks to the availability of HMMER3 (http://hmmer.janelia.org/), which achieves the speed of RPS-BLAST [14], and with the availability of the UFO web server [15], which is 100 times faster than RPS-BLAST, the detection of protein domains at genomic scale is no longer a computationally expensive task.

## Results

Our approach for phenotype prediction is based on the complete genomic sequences and the NCBI phenotype annotation of more than 1000 prokaryotic organisms from 21 different phylogenetic groups available at the NCBI web site http://www.ncbi.nlm.nih.gov/. The predictions were performed by applying a discriminative machine learning technique to the organisms' *protein domain profiles*, i.e. frequencies of Pfam [13] domain families in their genomes, and the organisms' phenotype annotations (see section "Methods"). As we will describe below, our approach has two important outcomes: (1) the accurate prediction of microbial phenotypes from organism-specific protein domain profiles and (2) the identification of discriminative, i.e. phenotype-specific, Pfam domain families.

First, we compared our approach to a method that can be used for microbial phenotype prediction and which is based on inferred metabolic pathways [8] (for details see section "Methods"). Table 1 shows the results of our evaluation in terms of the average values of the product of sensitivity and specificity and the area under ROC curve (aucROC, [16]) over 100 repetitions of a cross-validation procedure. The performance values for our domain-based approach are consistently higher than that of the pathway-based method: for both phenotype categories our approach achieves a 6 percentage point improvement in terms of the product of sensitivity and specificity.

**Table 1:** Performance comparison with pathway-based prediction

phenotype	best pathway-based		domain-based	
	
	aucROC	sens × spec	aucROC (avg/std)	sens × spec (avg/std)
Gram stain	0.93	0.90	0.97/0.01	0.96/0.01

Oxygen Requirement	0.93	0.88	0.95/0.02	0.94/0.02

Prediction performance comparison between a pathway-based method [8] and the protein domain profile-based approach. The first column indicates the phenotype category. The second and fourth column represent the phenotype prediction performance in terms of the area under ROC curve (aucROC), the third and fifth column denote the prediction performance in terms of the product of sensitivity (sens) and specificity (spec). For the domain-based method the values denote average (avg) and standard deviation (std) over 100 repetitions of a ten-fold cross-validation with random partitioning. Performance values for the pathway-based methods have been taken from the supplemental material associated with the original work.

Afterwards, we extended our evaluation to the whole set of organisms from the NCBI web site (for details see section "Methods"). Column 2 of table 2 shows the phenotype prediction performance of our domain-based approach in terms of the harmonic mean of sensitivity and specificity, which is also known as *F*_1_-measure. The average accuracy over all phenotype categories is high (0.954), indicating that the high-dimensional protein domain profile space provides a sufficient separability of examples by the linear classification model.

**Table 2:** Validation performance

Phenotype	no genus partition	with genus partition	difference
Endospores	0.946	0.821	-0.125
Gram stain	0.966	0.905	-0.061
Motility	0.932	0.870	-0.062
Oxygen Requirement	0.973	0.949	-0.024
average	0.954	0.886	-0.068

Comparison of phenotype prediction performance for different validation sets according to phylogenetic proximity of organisms (see also section "Methods"). The first column indicates the phenotype category, the second and third column represent the prediction performance in terms of the harmonic mean of sensitivity and specificity for the validation set without and with using the genus partition, respectively. Values in the fourth column correspond to the difference of prediction performance when the genus-partitioned data set is used.

Among the sequenced genomes available at the moment, several entries represent closely related organisms from the same genus or merely different strains from the same species (see also additional file 1). To estimate the prediction accuracy in case of the absence of those close relatives, we also evaluated our method on a genus-partitioned data set, i.e. organisms belonging to genera that have been used for training were not used for performance assessment. In the evaluation setup at hand this can also be seen as a rather rigorous redundancy reduction on a particular phylogenetic level. Column 3 of table 2 shows the performance values after genus partitioning. The average performance decline (shown in column 4 of table 2) is ≈ 7 percentage points, ranging from ≈ 2 percentage points for the phenotype category "Oxygen Requirement" to ≈ 13 percentage points for the "Endospores" category.

**Figure 1:**
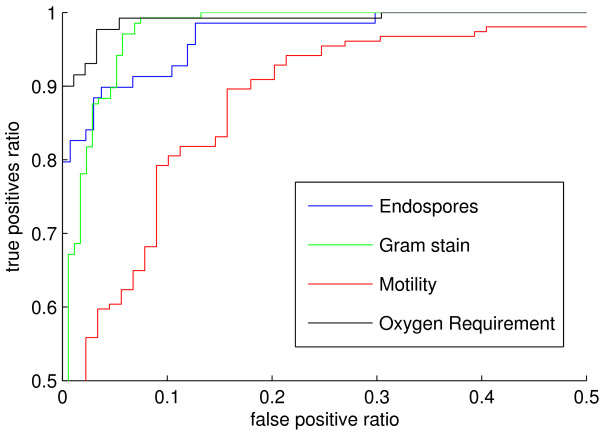
**ROC curves for generalization performance**. Receiver-operator characteristics (ROC) curves representing the phenotype prediction performance on the independent test set. Each phenotype-specific curve is assigned a unique color according to the legend within the figure. Axes are limited to minimum 50% true positive ratio and maximum 50% false positive ratio. The associated area under ROC curve (aucROC) values are given in table 3.

To estimate the generalization capability of our approach, we measured the prediction performance on an independent test set of 443 proteomes from organisms that have not been used in the validation procedure (see also section "Methods"). Table 3 shows the generalization performance in terms of the harmonic mean and other performance indices and figure 1 shows the associated ROC curves.

**Table 3:** Generalization performance

Phenotype	sensitivity	specificity	harmonic mean	aucROC	aucPRC
Endospores	0.913	0.875	0.894	0.984	0.959
Gram stain	0.993	0.907	0.948	0.986	0.968
Motility	0.942	0.874	0.906	0.927	0.944
Oxygen Requirement	0.992	0.963	0.977	0.993	0.987
average	0.960	0.904	0.931	0.972	0.965

Generalization performance using an independent test data set (for details see section "Methods"). The first column indicates the phenotype category, the remaining columns represent the prediction accuracy in terms of different performance measures. Here, "aucROC" and "aucPRC" correspond to "area under ROC curve" and "area under PRC curve", respectively.

In principle, our discriminative approach can be used with full genes as genomic features instead of (or in addition to) protein domains. To evaluate this, we also used clusters of orthologous genes (COGs, [17]) to construct organism-specific gene frequency profiles (see section "Methods"). On average, the validation performance is marginally lower as compared with the protein domain profiles (-0.003 without genus partition, -0.011 with genus partition). The same can be stated for the average generalization performance (-0.002), although here the sensitivity is slightly higher (+0.009).

So far, we limited our evaluation to binary cases of phenotypic traits, but in general, phenotype characteristics can have multiple values and may even be continuous. In contrast to other discriminative machine learning techniques, the method we used provides the possibility of multi-class learning (and classification by regression) as a standard feature (see also section "Methods"). Thus, we extended our evaluation to three phenotypes with multiple values: "Salinity" (with the traits "Extreme halophilic", "Mesophilic", "Moderate halophilic' and "Non-halophilic"), "Temperature range" ("Thermophilic", Hyperthermophilic" and "Mesophilic") and "Oxygen Requirement" including facultative organisms. Since the above mentioned performance indices are not defined for multi-class problems, we measured the generalization performance in terms of the average classification error. Here, the categories "Oxygen Requirement" and "Temperature range" show a good performance with average errors of ≈ 2% and ≈ 11%, respectively. In contrast, the phenotype category "Salinity" yields a classification error of ≈ 33%.

A potential application of the approach that we present here is the prediction of phenotypical traits for organisms that cannot be cultivated but whose genomes can be sequenced using single cell sequencing techniques or may be assembled from metagenomic sequence data because of the organism's abundance. As a pilot study, we analyzed two microbial genomes obtained from a single cell sequencing approach [12]. Our prediction for the two flavobacteria (*MS024-2A *and *MS024-3C*) indicate that both organisms do not form endospores, are gram negative, do not show evidence for motility and a weak evidence for an aerobic lifestyle. These results are in good agreement with what would be expected from flavobacteria in a marine environment.

## Discussion

As we showed in the previous section, our discriminative protein domain-based approach to microbial phenotype prediction provides a good validation and generalization performance and outperforms a method based on metabolic pathways [8]. The latter finding can possibly be explained by the fact that the pathway-based method was not intended for the purpose of phenotype prediction, but for the identification of phenotype-associated pathways. In contrast to our approach, which is designed to identify discriminative features from a large set of protein domains, the restriction to a few detectable metabolic pathways is likely to dismiss many of such discriminative features. Furthermore, the EC-based mapping of proteins to metabolic pathways as described in [8] complicates the practical application for *de novo *prediction of newly sequenced organisms. In contrast, our simpler approach only requires the phenotype-specific discriminative weight vectors and the organism's protein domain profile as obtained from a fast domain detection such as UFO [15].

Since protein domain sequences are often substantially shorter than the corresponding genes, domain detection can even be performed on short sequence fragments which do not contain full genes. This also qualifies our approach for phenotype prediction on sequence read data as obtained from high-throughput sequencing, providing a sufficient number of reads can be assigned to a particular species. However, if the complete protein sequences of an organism are available, our results indicate that clusters of orthologous genes (COGs) can be used as well to provide genomic features within our discriminative framework. This suggests that the choice between full genes and protein domains should depend on the kind of discriminative features one would like to obtain for further analysis (see below). In principle, our approach could also be used to study the importance of protein domain architecture for microbial genotype-phenotype association, e.g. by considering particular domain architectures as single features. However, this requires to deal with several rearrangements such as gene fusion, gene fission and domain shuffling, which lead to a high-dimensional and sparsely occupied feature space that complicates learning. Our results indicate a good generalization performance and thus a real world suitability of our approach. However, compared to the values obtained during validation, a decreased performance for all phenotype categories except "Oxygen Requirement" can be observed. We assume that this results from a combination of sequencing status and annotation quality of the organisms, which we used for the test set: the mostly unfinished genome sequences (337 out of 443) may lead to wrongly estimated domain profiles and the sometimes unverified phenotype annotation of these newly sequenced organisms may have been derived merely on the basis of phylogenetic proximity to well-studied organisms.

Our genus partition experiment simulated the absence of close species across the training and validation sets and showed that the prediction accuracy decreases differently across the phenotype categories. These results suggest that certain phenotypic traits can be predicted reliably without the presence of closely related species in the training data, while other phenotype categories lack a representative phylogenetic diversity in current databases and may thus depend on the presence of closely related organisms for accurate prediction. Similarly, our pre-study regarding multi-class phenotypes showed divergent accuracy results for the three tested phenotypes. In particular, the "Salinity" prediction performed poorly, with every third example being wrongly assigned. This may be explained by the low number of training examples for particular traits ("Extreme halophilic"/"Moderate halophilic": 8/18 examples) and the difficulty to unambiguously assign one of these phenotype characteristics to an organism.

### Investigation of discriminative domain families

In order to assess the biological relevance of the discriminative protein domain features, we investigate the 50 most discriminative phenotype-specific domain families within a biological context. For brevity, we restrict the analysis to the two phenotype categories "Endospores" (spore formation) and "Motility". However, the investigation can be performed in a similar fashion for the "Gram stain" and "Oxygen Requirement" phenotypes using the ranked lists of discriminative domain families provided in additional file 2. The features are based on the discriminative model that has been learned from all training organisms using the parameter associated with the best validation performance. We also discuss the results from the phylogenetic clustering of these families, which may be used to identify functional modules of domains (for details see section "Methods"). The clustering dendrograms associated with all phenotypes can be found in additional file 3.

#### Endospores

Endospores are dormant resistance forms produced by some gram-positive bacteria by the formation of a thick internal wall that encloses the DNA and a part of the cytoplasm. For reasons of brevity, table 4 only shows the 10 most discriminative domain families associated with the phenotype category "Endospores", a list with the 50 most discriminative domains can be found in additional file 2. Of the top 10 (50) domains, 4 (12) have the terms "spore" or "sporulation" in their description line. Additional domains are directly involved in sporulation, e.g. the Cell wall hydrolase, the Coat F domain, the Germination protease, or the YabG peptidase U57 domain, which is present in a sporulation-specific protease of several spore coat proteins [18]. Other domains such as YabP have been associated with the sporulation process through mutagenesis analyses. Thus, the discriminative domain set for prokaryotes associated with the term "Endospore formation" is enriched in domains involved in the sporulation process.

**Table 4:** Discriminative domain families for phenotype category "Endospores"

rank	weight	# groups	Pfam-ID	Pfam description
1	0.008	3	PF03419	Sporulation factor SpoIIGA

2	0.007	5	PF07486	Cell Wall Hydrolase

3	0.007	1	PF06686	Stage III sporulation protein AC (SpoIIIAC)

4	0.007	2	PF00269	Small, acid-soluble spore proteins, alpha/beta type

5	0.007	1	PF07873	YabP family

6	0.007	1	PF09555	Stage III sporulation protein AD (spore_III_AD)

7	0.007	3	PF00407	Pathogenesis-related protein Bet v I family

8	0.007	4	PF00876	Innexin

9	0.007	6	PF04672	Protein of unknown function (DUF574)

10	0.006	1	PF04647	Accessory gene regulator B

List of the 10 most (positive) discriminative domain families associated with the RLSC model for the phenotype category "Endospores" (see also section "Methods"). The first column indicates the rank, the second column shows the discriminative model weight. The third column denotes the phylogenetic width of a particular domain family, i.e. the number of taxonomic groups at phylum level in which the family occurs. The fourth and fifth column correspond to the Pfam ID and family description associated with a particular domain family. The table with the 50 most positively and negatively discriminative domain families can be found in additional file 2.

**Figure 2:**
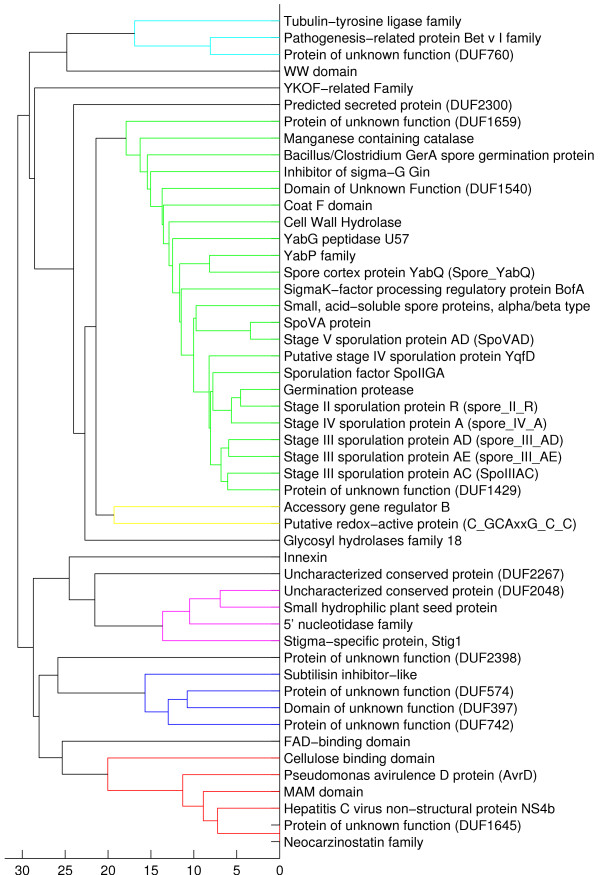
**Clustering dendrogram for phenotype category "Endospores"**. Phylogenetic clustering dendrogram of the 50 most discriminative positive domains associated with the phenotype category "Endospores" (see also section "Methods"). Clusters with a maximum linkage branch length of 70% (Matlab^® ^'dendrogram' default threshold) are assigned unique colors. In the dendrogram a cluster of protein families associated with sporulation can directly be identified (colored green). This cluster also contains several uncharacterized domain families ("DUFs").

Figure 2 shows the dendrogram resulting from phylogenetic clustering of the 50 most discriminative domain families. In the upper part, a cluster of Pfam domains associated with sporulation can easily be identified (colored green). Interestingly, also three domains of unknown function ("DUFs") are localized in that cluster (DUF1659, DUF1540, DUF1429). This indicates that the proteins associated with these families may directly be involved in the sporulation process or act together with sporulation proteins in the same pathway. In fact, the domain family DUF1429 (PF07241) located at the lower part of the cluster is considered a "dead" family in the current version (24.0) of the Pfam database and has been merged into the family YabP (PF07873), which is associated with sporulation.

#### Motility

Of the top 10 (50) domains associated with the phenotype "Motility" (see table 5 and additional file 2), 6 (24) are known components of the flagellar machinery, the macromolecular complex responsible for bacterial motility, including SPOA and SAF domains. Other groups of domains associated with this phenotype are related to the secretory pathway specifically devoted to export components of the flagellum, these include FHIPEP, FlhB, FliF, FliP, and Bacterial export families 1 and 3. Additional three domains are associated to the process of translation and it could be postulated that they are specific factors for transcribing genes related to the domain families mentioned above. For instance, sigma-54 has recently been found to be involved in the flagellum in *E. coli *[19].

**Table 5:** Discriminative domain families for phenotype category "Motility"

rank	weight	# groups	Pfam-ID	Pfam description
1	0.008	18	PF00015	Methyl-accepting chemotaxis protein (MCP) signaling domain

2	0.007	20	PF00672	HAMP domain

3	0.007	14	PF00460	Flagella basal body rod protein

4	0.006	12	PF08345	Flagellar M-ring protein C-terminal

5	0.006	15	PF06429	Domain of unknown function (DUF1078)

6	0.006	14	PF00700	Bacterial flagellin C-terminus

7	0.006	16	PF01312	FlhB HrpN YscU SpaS Family

8	0.006	11	PF02120	Flagellar hook-length control protein

9	0.006	14	PF02049	Flagellar hook-basal body complex protein FliE

10	0.006	14	PF00669	Bacterial flagellin N-terminus

List of the 10 most (positive) discriminative domain families associated with the RLSC model for the phenotype category "Motility" (see also section "Methods"). The first column indicates the rank, the second column shows the discriminative model weight. The third column denotes the phylogenetic width of a particular domain family. The fourth and fifth column correspond to the Pfam ID and family description associated with a particular domain family. The table with the 50 most positively and negatively discriminative domain families can be found in additional file 2.

Another group of domains that is highly represented in this set is the one related to chemotaxis, the mechanism by which cells can direct their movement in response to certain stimuli. These include MCP, CheW and CheC domains, the Cache and P2 response regulator domains, the signal transducing histidine kinase and its related HAMP domain. It is obvious that all bacteria able of chemotaxis do need a motility system and thus this association is to be expected.

The clustering dendrogram associated with the motility phenotype (see additional file 3) is dominated by a cluster that groups together the domain families known to be involved in the flagellar structure and protein families associated with chemotaxis (colored green). This cluster also includes a domain of unknown function (DUF1078, PF06429), indicating that this DUF is highly correlated to flagellar basal-body rod and flagellar hook proteins. Indeed, the Pfam annotation of DUF1078 suggests a relation to these families and contains the gene ontology (GO) cellular component category "flagellum". The DUF1078 domain family has also been identified to be correlated with motility by Liu et al. [5].

### Detecting annotation errors

A possible use of phenotype prediction approaches is the completion and correction of existing annotation. In order to identify erroneous NCBI annotation associated with the organisms, we compared our prediction results with the phenotype annotation and verified the candidates via literature search. We found several cases of possible annotation errors for different categories by verifying putatively false predictions on the test set that do not agree with the existing annotation. For instance, for the "Motility" category we identified two organisms (*Burkholderia glumae BGR1, Bacillus cereus 03BB102*) with negative annotation but evidence for being motile [20,21]. Similarly, for the category "Gram stain" we found another two possible erroneous annotations (*Eubacterium eligens ATCC 27750, Geodermatophilus obscurus DSM 43160*) with phenotype annotation in the literature differing from that of the NCBI [22,23]. Finally, we identified two organisms that most likely have been assigned to the wrong respiratory subcategory (*Dethiosulfovibrio peptidovorans DSM 11002, Acidimicrobium ferrooxidans DSM 10331 *[24,25]). In order to encourage the systematic correction of erroneous existing annotation, we provide phenotype-specific lists with prediction results from our approach for organisms in the independent test in additional file 4.

### Robustness and speed

Our validation (test) data set consists of 795 (106) finished and 237 (337) unfinished genomes, respectively. The total number of examples used for model training and validation is substantially higher as compared to related work of e.g. Slonim et al. [6] (59 genomes) or Kastenmüller et al. [8] (266 genomes). We think that the robustness of models, which is indicated by the good generalization performance, can partly be attributed to the unprecedented size of the data set. Furthermore, the high prediction accurracy on the test set, which contains many yet unfinished genomes, suggests that even in early stages of genome projects reliable statements about an organism's phenotype can be derived from the predicted domain profile.

In contrast to the approach presented in [8], our method does not involve an intermediate mapping of proteins to pathways. Instead, the phenotype prediction is directly performed on the basis of estimated Pfam domain profiles. The computation time that is necessary for inference of the domain profiles depends on the domain detection method and the size of an organism's proteome and ranges from a few seconds to several hours [15]. The actual phenotype prediction can be efficiently performed by means of the dot product of the precomputed domain profile and the discriminative weight vector in domain profile space. This only requires storage of the 10797-dimensional discriminant and ≈ 0.05 milliseconds for calculation of the prediction score.

## Conclusions

In this work we presented a discriminative machine learning approach that allows to accurately predict the phenotype of a prokaryotic organism directly from its protein domain frequencies. Our results indicate that the domain-based method achieves a better prediction accuracy than the pathway-based approach of Kastenmüller et al. [8], while being simpler to implement. Furthermore, we showed that the learned models can be interpreted within a biologically meaningful context in terms of discriminative protein domain families associated with a particular phenotype. By means of a cluster analysis of these families we also identified phenotype-specific functional modules that contain yet uncharacterized protein domain families ("DUFs") and thus may be used to generate hypotheses about the functions of these domains. As an alternative to protein domains, we showed that clusters of orthologous genes (COGs, [17]) may also be used as genomic features for discriminative learning. Similar to our analysis of discriminative protein domains, this might be valuable for interpretation of the resulting phenotype-specific genes. The incorporation of gene context features such as protein domain architecture is possible with our approach and shall be investigated in the future.

In combination with a fast domain detection method, our approach can be used to rapidly predict the phenotype of an organism solely based on its genomic data. As we showed in this work, this can be achieved in very early stages even before genome assembly. In principle, our method can also be used to verify and complete the phenotype annotation in public databases, e.g. within the NCBI prokaryotic genome project. Future work will include the estimation of the reliability of the existing annotation based on differences between phenotype predictions and literature information. By this means, we hope to gradually improve the data underlying our method and thus the accurracy of the resulting models. Furthermore, the framework shall be extended to deal with more detailed and diverse phenotype description, e.g. as provided by the expected growth of metadata associated with sequenced genomes.

## Methods

### Dataset

We downloaded all prokaryotic proteomes from the integr8 FTP site ftp://ftp.ebi.ac.uk/pub/databases/integr8/fasta/proteomes/ on May 12, 2009 and selected all NCBI annotated organisms which could be uniquely associated with an integr8 proteome file via the integr8 proteome report ftp://ftp.ebi.ac.uk/pub/databases/integr8/proteome_report.txt. The annotation of these 1032 organisms for the phenotypes "Endospores", "Gram stain", "Motility" and "Oxygen Requirement" was extracted from the respective columns of the table on the NCBI prokaryotic genome project web site ftp://ftp.ncbi.nlm.nih.gov/genomes/genomeprj/lproks_0.txt. While these proteomes were used for the validation procedure (see below), we downloaded additional 443 proteomes and the associated annotation from the abovementioned websites on October 10, 2009. The complete list of organisms and their phenotype annotation can be found in additional file 1.

### Construction of Pfam domain and COG profiles

In this work, the Pfam domain profile associated with a particular organism is a *d *= 10797 dimensional domain occurrence vector according to the maximum family index of PF10797 in version 23.0, section "A" of the Pfam database [13]. This vector has nonzero entries only for dimensions associated with the Pfam domains that occur in the organism's genome. To calculate the occurrences, we applied a Pfam domain detection to each protein in each genome using the UFO method [15]. The resulting vector **x **of absolute domain occurrence counts is normalized to relative domain frequencies such that $x_{f}\in {\left[0,1\right]}^{d},\sum_{i=1}^{d}x_{i}=1$

Furthermore, each dimension of the domain feature space with a standard deviation different from zero is normalized to unit standard deviation. The data matrices with UFO counts for all organisms of the validation and test set along with the organism names and relevant NCBI phenotype annotation in comma separated value (CSV) format can be found in additional file 5.

For comparison, we also calculated organism-specific profiles of full gene frequencies in terms of clusters of orthologous genes (COGs, [17]) detected in the organisms' protein sequences. For COG detection, we downloaded the COG database in RPS-BLAST (Reverse PSI-BLAST, [26]) format from ftp://ftp.ncbi.nih.gov/pub/mmdb/cdd/little_endian/Cog_LE.tar.gz and evaluated the organisms' proteomes against this database using the default e-value threshold of 0.01. The 5665 COG clusters give rise to 5665-dimensional organism-specific feature vectors, which we normalized as described above.

### Phenotype prediction on domain profiles

To predict microbial phenotypes from organism-specific domain profiles, we use a supervised classification approach. For this purpose, we divide the task of predicting four phenotype categories into four binary classification problems according to the discrimination of different phenotype realizations. Here, organisms that are annotated as "yes" ("Endospores"), "+" ("Gram stain"), "motile" ("Motility") or "aerobic" ("Oxygen Requirement") are considered as positive examples and organisms that are annotated "no"/"-"/"non-motile"/"anaerobic" are used as negative examples of the respective two-class problem. Domain profiles of organisms that do not have any annotation for a particular phenotype realization are not considered for evaluation. Furthermore, organisms with an identical UFO Pfam domain profile were reduced to a single representative organism. Thus, the total number of examples as well as the number of positive and negative examples vary across the different phenotype categories. An overview of the number of examples associated with each phenotype and the phylogenetic distributions of all phenotypes in terms of histograms can be found in additional file 6.

For learning of discriminative phenotype prediction models we use so-called regularized least-squares classifiers (RLSC, [27]). RLS classifiers are closely related to widely-used Support Vector Machines (SVM) and have been shown to provide similar classification performance, while being simpler to implement [28]. Furthermore, the RLSC method is computationally efficient and the learned discriminative weight vector can be interpreted in terms of underlying features. To take into account the imbalanced number of positive and negative examples in each category, we apply a "balanced" implementation of RLSC [29]. The balanced RLSC error function for *N *examples and a regularization parameter *λ *can be written as

(1) $$E(w)=\sum_{i=1}^{N}b_{i}{\left\|y_{i}-w^{t}x_{i}\right\|}^{2}+\lambda {\left\|w\right\|}^{2},$$

where **w **is the discriminative weight vector and *y_i _*∈ {-1, 1} is the class label of the *i*-th example. The vector **b **contains the example-specific balancing factors, whereby we use the inverse size of the class a particular example belongs to. For reasons of computational efficiency, we apply the RLSC method in a kernel-based manner using a linear kernel, whereby the kernel matrix **K **of all examples is computed by **K **= **X***^t^***X**. The matrix **X **corresponds to the matrix of all *N *organism-specific domain profiles **X **= [**x**_1_,..., **x***_N_*]. The abovementioned error function can now be minimized by

(2) $$w={\left(K+\lambda B\right)}^{-1}y,$$

where **B **is a matrix that contains the inverse elements of the vector **b **as diagonal elements. In case of multi-class learning problems with *M *classes, the label vector **y **has to be replaced by a matrix **Y **= [**z**_1_,..., **z***_M_*], *z_i, j _*∈ {0, 1}, where **z***_i _*is a class-specific vector with non-zero values for examples belonging to class *i*. The minimization then yields a matrix **W **= [**w**_1_,..., **w***_M_*] of *M *class-specific discriminative weight vectors.

To evaluate the influence of the regularization parameter *λ*, we randomly divided the data set into 20 partitions with 70% training and 30% validation examples, respectively. Using these partitions, we computed the average area under curve with respect to the precision-recall characteristics (PRC, [16]) over all partitions to determine the best parameter *λ *= {10*^m^*|*m *= -5, -4,..., 5}. Finally, we tested the prediction performance of our method in terms of the harmonic mean (also known as *F*_1_-measure) $harm=\frac{2*sens*spec}{sens+spec}$, which combines sensitivity $\left(sens=\frac{TP}{TP+FN}\right)$ and specificity $\left(spec=\frac{TP}{TP+FP}\right)$. To estimate the generalization performance of our approach on the set of 443 test genomes, we evaluated the discriminative model associated with the highest validation performance.

The kernel-based RLSC model associated with a phenotype classification problem is represented by a vector of *N *organism-specific weights. For fast prediction of phenotypes, the discriminant in the original feature space of Pfam protein domain profiles can be calculated by a linear combination of the learned organism-specific weights and the domain profiles in **X**. The phenotype prediction for a newly sequenced organism then only requires the construction of the organism's Pfam domain profile and the computation of the dot product of the feature space discriminant and the domain profile.

The feature space discriminant also allows to inspect the learned discriminative features in terms of phenotype-specific Pfam domain families. For each phenotype prediction problem, we assemble two ranked lists of domain families that are associated with the 50 largest positive and negative weights in the profile space discriminant, respectively. This allows to identify indicative and counter-indicative domain families directly.

For further interpretation of the abovementioned lists and for identification of functional modules, we also calculate clustering dendrograms for the 50 most discriminative domain families based on their phylogenetic profile, i.e. the absence/presence pattern of a particular domain across all organisms. To calculate the dendrograms, we apply the Matlab^® ^function 'dendrogram' to the domain phylogenetic profiles using average linkage (UPGMA) and the profile correlation as a distance measure. Dendrogram branch colors are calculated by the 'dendrogram' function using the default color threshold at 70% linkage branch length.

### Comparison with pathway-based prediction

For comparison to a method that can in principle be used for microbial phenotype prediction, we evaluate our approach using the dataset presented in [8]. Because of the different set of phenotypic traits used in the two works, only the two phenotypes "Gram stain" and "Oxygen Requirement" can be directly compared. As in [8], we selected the organisms associated with the phenotypical traits "aerobic"/"anaerobic" and "gram positive"/"gram negative" as the respective positive and negative examples of the phenotype category. In Kastenmüller et al. [8] the commercial Pedant database [30] has been used for collection of genome features and phenotype annotation. As a consequence, not all organisms present in the Pedant database can be found in the NCBI prokaryote genome project. For our evaluation, we used the data of all organisms whose taxonomy ID could be mapped to a NCBI taxonomy ID. In total, we used 166/92 out of 201/113 possible organisms for the phenotype categories "Gram stain"/"Oxygen Requirement", respectively. However, our inspection of the list of all organisms revealed that most of the missing organisms have identical species names when compared with the present organisms, thus the performance values we obtain here can be expected to provide lower bounds.

For direct comparison of the results, we measure the prediction performance in terms of the area under ROC curve [16] and the product of sensitivity and specificity as in [8]. In the original work, a ten-fold cross validation procedure has been performed. To get a reliable estimate of the prediction performance, we repeated the cross validation 100 times using random partitions. Furthermore, we compared our approach to the best of the four classification methods used in [8] as indicated by the classification quality diagrams in additional file 2 of the original work.

## Authors' contributions

TL carried out experiments and wrote the manuscript. SM carried out experiments and performed annotation check. TG performed biological interpretation of the results and wrote the manuscript. CN contributed conceptually and wrote the manuscript. PM designed the experiments, collected the data and wrote the manuscript. All authors read and approved the final manuscript.

## Supplementary Material

Additional file 1 **List of organisms for validation and test**. The file "organisms.pdf" contains the names of all 1032 (443) organisms that have been used for validation (test) of the classification methods. The table also shows the sequencing status and the phenotype annotation associated with the organisms. Click here for file

Additional file 2 **Lists of phenotype-specific discriminative domain families**. The archive "discDomains.zip" contains lists of the 50 most discriminative (indicative and counterindicative) Pfam domain families associated with the four phenotype categories "Endospores","Gram stain", "Motility" and "Oxygen Requirement" in HTML format. Click here for file

Additional file 3 **Clustering dendrograms of discriminative domain families**. The file "Dendrograms.pdf" contains the phenotype-specific phylogenetic clustering dendrograms for the 50 most discriminative domain families. For the phenotype categories "Gram stain" and "Oxygen Requirement" dendrograms for positive and negative discriminative domains are shown, for the "Endospores" and "Motility" phenotype categories only the dendrogram associated with positive discriminative domains is shown. Click here for file

Additional file 4 **Comparison of predicted phenotypes to NCBI annotation**. The file "DeviationNCBI.pdf" contains the prediction results of the domain-based approach for the set of 443 test organisms separated according to NCBI phenotype categories for the four phenotypes used in this study. The lists contain the predicted labels as well as the original label according to the NCBI annotation; label differences are highlighted using a red cell background. Furthermore, organisms that have been identified as possibly containing a wrong NCBI phenoype annotation in section "Quality of annotation" are highlighted using a bold face type. Click here for file

Additional file 5 **Protein domain profile data**. The file "EvaluationData.zip" consists of two comma separated value (CSV) files containing the data matrices with UFO counts associated with all organisms of the validation and test set, respectively. Here, each column corresponds to one organism and each row corresponds to one of the 10797 Pfam-A (version 23.0) families PF00001,..., PF10797. In addition, two CSV files contain the list of organism names and the associated NCBI phenotype annotation for the categories used in this study. Click here for file

Additional file 6 **Histograms of phenotype-specific phylogenetic distribution of example organisms**. The file "histoGroups.pdf" contains phylum-level histogram plots of the phenotype-specific number of positive and negative examples. Click here for file
