# Supplementary material for: Predicting phenotypic traits of prokaryotes from protein domain frequencies
Source: BMC Bioinformatics. 2010 Sep 24;11:481. doi: 10.1186/1471-2105-11-481 (PMC2955703; doi:10.1186/1471-2105-11-481)
Supplement: Additional file 1 — List of organisms for validation and test. The file "organisms.pdf" contains the names of all 1032 (443) organisms that have been used for validation (test) of the classification methods. The table also shows the sequencing status and the phenotype annotation associated with the organisms. [file 1471-2105-11-481-S1.PDF]

Organisms used for validation set

				phenotype categories		
name	*sequence status*	*taxonomic group*	*Endospores*	*Gram Stain*	*Motility*	*Oxygene Req.*
Acaryochloris marina MBIC11017	complete	Cyanobacteria		_		Aerobic
Acholeplasma laidlawii PG-8A	complete	Firmicutes		_		Facultative
Acidiphilium cryptum JF-5	complete	Alphaproteobacteria		-		Aerobic
Acidithiobacillus ferrooxidans ATCC 23270	complete	Gammaproteobacteria		-	Yes	Facultative
Acidithiobacillus ferrooxidans ATCC 53993	complete	Other Bacteria	No	-	Yes	Aerobic
Acidobacteria bacterium Ellin345	complete	Acidobacteria		_		
Acidothermus cellulolyticus 11B	complete	Actinobacteria	Yes	+		Aerobic
Acidovorax citrulli AAC00-1	complete	Betaproteobacteria	No	-	Yes	Aerobic
Acidovorax sp. JS42	complete	Betaproteobacteria	No	-	Yes	Aerobic
Acinetobacter baumannii	unfinished	Gammaproteobacteria		-		Aerobic
Acinetobacter baumannii AB0057	complete	Gammaproteobacteria	No	-	No	Aerobic
Acinetobacter baumannii AB307-0294	complete	Gammaproteobacteria		-		Aerobic
Acinetobacter baumannii ACICU	complete	Gammaproteobacteria		-		Aerobic
Acinetobacter baumannii ATCC 17978	complete	Gammaproteobacteria	No	-	No	Aerobic
Acinetobacter baumannii SDF	complete	Gammaproteobacteria		-		Aerobic
Acinetobacter sp. ADP1	complete	Gammaproteobacteria	No	-	No	Aerobic
Actinobacillus pleuropneumoniae L20	complete	Gammaproteobacteria		-		Facultative
Actinobacillus pleuropneumoniae serovar 3 str. JL03	complete	Gammaproteobacteria		-		Facultative
Actinobacillus pleuropneumoniae serovar 7 str. AP76	complete	Gammaproteobacteria		-		Facultative
Actinobacillus succinogenes 130Z	complete	Gammaproteobacteria		-		
Aeromonas hydrophila subsp. hydrophila ATCC 7966	complete	Gammaproteobacteria		-	Yes	Facultative
Aeromonas salmonicida subsp. salmonicida A449	complete	Gammaproteobacteria		-	Yes	Facultative
Aeropyrum pernix K1	complete	Crenarchaeota	No	_	Yes	Aerobic
Agrobacterium radiobacter K84	complete	Alphaproteobacteria		-	Yes	Aerobic
Agrobacterium tumefaciens str. C58	complete	Alphaproteobacteria		-	Yes	Aerobic
Agrobacterium vitis S4	complete	Alphaproteobacteria		-	Yes	Aerobic
Akkermansia muciniphila ATCC BAA-835	complete	Other Bacteria	No	-	No	Anaerobic
Alcanivorax borkumensis SK2	complete	Gammaproteobacteria		-	No	Aerobic
Algoriphagus sp. PR1	assembly	Bacteroidetes/Chlorobi		_		
Alicyclobacillus acidocaldarius LAA1	assembly	Firmicutes	Yes	+		Aerobic
Aliivibrio salmonicida LFI1238	complete	Gammaproteobacteria	No	-	Yes	Facultative
Alkalilimnicola ehrlichei MLHE-1	complete	Gammaproteobacteria		_		Facultative
Alkaliphilus metalliredigens QYMF	complete	Firmicutes		_		
Alkaliphilus oremlandii OhILAs	complete	Firmicutes	Yes	+		Anaerobic
alpha proteobacterium BAL199	assembly	Alphaproteobacteria		_		
Alteromonadales bacterium TW-7	assembly	Gammaproteobacteria		_		
Alteromonas macleodii 'Deep ecotype'	complete	Gammaproteobacteria		-	Yes	Aerobic
Anabaena variabilis ATCC 29413	complete	Cyanobacteria		_	Yes	Aerobic
Anaerocellum thermophilum DSM 6725	complete	Firmicutes		_		Anaerobic
Anaerococcus hydrogenalis DSM 7454	assembly	Firmicutes		_		
Anaeromyxobacter dehalogenans 2CP-1	complete	Deltaproteobacteria	Yes	-	Yes	Facultative
Anaeromyxobacter dehalogenans 2CP-C	complete	Deltaproteobacteria	Yes	-	Yes	Facultative
Anaeromyxobacter sp. Fw109-5	complete	Deltaproteobacteria	Yes	_	Yes	Anaerobic
Anaeromyxobacter sp. K	complete	Deltaproteobacteria	Yes	_	Yes	Anaerobic
Anaplasma marginale str. Florida	complete	Alphaproteobacteria		_		Aerobic
Anaplasma marginale str. St. Maries	complete	Alphaproteobacteria		_		Aerobic
Anaplasma phagocytophilum HZ	complete	Alphaproteobacteria		-		Aerobic
Anoxybacillus flavithermus WK1	complete	Firmicutes	Yes	+	Yes	Facultative
Aquifex aeolicus VF5	complete	Aquificae		-		Aerobic
Archaeoglobus fulgidus DSM 4304	complete	Euryarchaeota		_	Yes	Anaerobic
Arcobacter butzleri RM4018	complete	Epsilonproteobacteria	No	-	Yes	Aerobic
Aromatoleum aromaticum EbN1	complete	Betaproteobacteria		-	Yes	Facultative
Arthrobacter aurescens TC1	complete	Actinobacteria	No	+	Yes	Aerobic
Arthrobacter chlorophenolicus A6	complete	Actinobacteria	No	+	Yes	Aerobic
Arthrobacter sp. FB24	complete	Actinobacteria		+		
Arthrospira maxima CS-328	assembly	Cyanobacteria		_		Facultative
Aster yellows witches'-broom phytoplasma AYWB	complete	Firmicutes		_		Aerobic
Azoarcus sp. BH72	complete	Betaproteobacteria		-	Yes	Microaerophilic
Azorhizobium caulinodans ORS 571	complete	Alphaproteobacteria		_		
Bacillus amyloliquefaciens FZB42	complete	Firmicutes	Yes	+		Aerobic
Bacillus anthracis str. 'Ames Ancestor'	complete	Firmicutes	Yes	+	Yes	Facultative
Bacillus anthracis str. A0174	assembly	Firmicutes	Yes	+	Yes	Facultative
Bacillus anthracis str. A0193	assembly	Firmicutes	Yes	+	Yes	Facultative
Bacillus anthracis str. A0389	assembly	Firmicutes	Yes	+	Yes	Facultative
Bacillus anthracis str. A0442	assembly	Firmicutes	Yes	+	Yes	Facultative
Bacillus anthracis str. A0465	assembly	Firmicutes	Yes	+	Yes	Facultative
Bacillus anthracis str. A0488	assembly	Firmicutes	Yes	+	Yes	Facultative
Bacillus anthracis str. Ames	complete	Firmicutes	Yes	+	Yes	Facultative
Bacillus anthracis str. Sterne	complete	Firmicutes	Yes	+	Yes	Facultative
Bacillus anthracis Tsiankovskii-I	assembly	Firmicutes	Yes	+	Yes	Facultative
Bacillus cereus 03BB108	assembly	Firmicutes	Yes	+	Yes	Aerobic
Bacillus cereus AH1134	assembly	Firmicutes	Yes	+	Yes	Aerobic
Bacillus cereus AH187	complete	Firmicutes	Yes	+	Yes	Aerobic
Bacillus cereus AH820	complete	Firmicutes	Yes	+	Yes	Aerobic
Bacillus cereus ATCC 10987	complete	Firmicutes	Yes	+	Yes	Aerobic
Bacillus cereus ATCC 14579	complete	Firmicutes	Yes	+	Yes	Aerobic
Bacillus cereus B4264	complete	Firmicutes	Yes	+	Yes	Aerobic
Bacillus cereus E33L	complete	Firmicutes	Yes	+	Yes	Aerobic
Bacillus cereus G9241	assembly	Firmicutes	Yes	+	Yes	Aerobic
Bacillus cereus G9842	complete	Firmicutes	Yes	+	Yes	Aerobic
Bacillus cereus NVH0597-99	assembly	Firmicutes	Yes	+	Yes	Aerobic
Bacillus cereus Q1	complete	Firmicutes	Yes	+	Yes	Aerobic
Bacillus cereus subsp. cytotoxis NVH 391-98	complete	Firmicutes	Yes	+	Yes	Aerobic
Bacillus cereus W	assembly	Firmicutes	Yes	+	Yes	Aerobic
Bacillus clausii KSM-K16	complete	Firmicutes		_		
Bacillus coagulans 36D1	assembly	Firmicutes	Yes	+	Yes	Facultative
Bacillus halodurans C-125	complete	Firmicutes	Yes	+	Yes	Facultative
Bacillus licheniformis ATCC 14580	complete	Firmicutes	Yes	+	Yes	Facultative
Bacillus pumilus ATCC 7061	assembly	Firmicutes	Yes	+	Yes	Aerobic
Bacillus pumilus SAFR-032	complete	Firmicutes	Yes	+	Yes	Aerobic
Bacillus selenitireducens MLS10	assembly	Firmicutes	No	+	No	Facultative
Bacillus sp. B14905	assembly	Firmicutes	Yes	+		Aerobic
Bacillus sp. SG-1	assembly	Firmicutes	Yes	+		Aerobic
Bacillus subtilis subsp. subtilis str. 168	assembly	Firmicutes		_		

			phenotype categories			
name	*sequence status*	*taxonomic group*	*Endospores*	*Gram Stain*	*Motility*	*Oxygene Req.*
Bacillus thuringiensis serovar israelensis ATCC 35646	assembly	Firmicutes	Yes	+	Yes	Facultative
Bacillus thuringiensis serovar konkukian str. 97-27	complete	Firmicutes	Yes	+	Yes	Facultative
Bacillus thuringiensis str. Al Hakam	complete	Firmicutes	Yes	+	Yes	Facultative
Bacillus weihenstephanensis KBAB4	complete	Firmicutes		+		Aerobic
Bacteroides caccae ATCC 43185	assembly	Bacteroidetes/Chlorobi		-		Anaerobic
Bacteroides fragilis NCTC 9343	complete	Bacteroidetes/Chlorobi		-		Anaerobic
Bacteroides fragilis YCH46	complete	Bacteroidetes/Chlorobi		-		Anaerobic
Bacteroides thetaiotaomicron VPI-5482	complete	Bacteroidetes/Chlorobi		-		Anaerobic
Bacteroides vulgatus ATCC 8482	complete	Bacteroidetes/Chlorobi		+		Anaerobic
Bartonella bacilliformis KC583	complete	Alphaproteobacteria	No	-	Yes	Aerobic
Bartonella quintana str. Toulouse	complete	Alphaproteobacteria	No	-		Aerobic
Bartonella tribocorum CIP 105476	complete	Alphaproteobacteria	No	-	Yes	Aerobic
Baumannia cicadellinicola str. Hc (Homalodisca coagulata)	complete	Gammaproteobacteria		_		
Beggiatoa sp. PS	assembly	Gammaproteobacteria		_		
Beggiatoa sp. SS	assembly	Gammaproteobacteria		_		
Beijerinckia indica subsp. indica ATCC 9039	complete	Alphaproteobacteria		-	No	Aerobic
Bifidobacterium adolescentis ATCC 15703	complete	Actinobacteria	No	+	No	Anaerobic
Bifidobacterium animalis subsp. lactis AD011	complete	Actinobacteria	No	+	No	Anaerobic
Bifidobacterium animalis subsp. lactis HN019	assembly	Actinobacteria		+	No	Anaerobic
Bifidobacterium catenulatum DSM 16992	assembly	Actinobacteria		_		
Bifidobacterium longum DJO10A	complete	Actinobacteria	No	+	No	Anaerobic
Bifidobacterium longum NCC2705	complete	Actinobacteria	No	+	No	Anaerobic
Bifidobacterium longum subsp. infantis ATCC 15697	complete	Actinobacteria	No	+	No	Anaerobic
Bordetella avium 197N	complete	Betaproteobacteria		-	Yes	Aerobic
Bordetella parapertussis 12822	complete	Betaproteobacteria		-		Aerobic
Borrelia afzelii PKo	complete	Spirochaetes	No	-	Yes	Aerobic
Borrelia burgdorferi B31	complete	Spirochaetes	No	_	Yes	Microaerophilic
Borrelia burgdorferi ZS7	complete	Spirochaetes	No	_	Yes	Microaerophilic
Borrelia duttonii Ly	complete	Spirochaetes	No	_	Yes	Aerobic
Borrelia garinii PBi	complete	Spirochaetes	No	_	Yes	
Borrelia hermsii DAH	complete	Spirochaetes	No	_	Yes	Aerobic
Borrelia recurrentis A1	complete	Spirochaetes	No	-	Yes	Aerobic
Borrelia turicatae 91E135	complete	Spirochaetes	No	_	Yes	Aerobic
Brachyspira hyodysenteriae WA1	complete	Spirochaetes	No	-	Yes	Facultative
Bradyrhizobium japonicum USDA 110	complete	Alphaproteobacteria		-	Yes	Aerobic
Bradyrhizobium sp. BTAi1	complete	Alphaproteobacteria		-	Yes	Aerobic
Bradyrhizobium sp. ORS278	complete	Alphaproteobacteria		_		
Brucella abortus bv. 1 str. 9-941	complete	Alphaproteobacteria		-		Facultative
Brucella abortus S19	complete	Alphaproteobacteria		-		Facultative
Brucella canis ATCC 23365	complete	Alphaproteobacteria		-		
Brucella melitensis 16M	unfinished	Alphaproteobacteria		_		
Brucella melitensis biovar Abortus 2308	complete	Alphaproteobacteria		-		Facultative
Brucella ovis ATCC 25840	complete	Alphaproteobacteria	No	-	No	Facultative
Brucella suis 1330	complete	Alphaproteobacteria		-		Aerobic
Brucella suis ATCC 23445	complete	Alphaproteobacteria		-		Aerobic
Buchnera aphidicola str. 5A (Acyrthosiphon pisum)	complete	Gammaproteobacteria		-		
Buchnera aphidicola str. APS (Acyrthosiphon pisum)	complete	Gammaproteobacteria		-		
Buchnera aphidicola str. Bp (Baizongia pistaciae)	complete	Gammaproteobacteria		-		
Buchnera aphidicola str. Cc (Cinara cedri)	complete	Gammaproteobacteria		-		
Buchnera aphidicola str. Sg (Schizaphis graminum)	complete	Gammaproteobacteria		-		
Buchnera aphidicola str. Tuc7 (Acyrthosiphon pisum)	complete	Gammaproteobacteria		-		
Burkholderia ambifaria AMMD	complete	Betaproteobacteria	No	-	Yes	Facultative
Burkholderia ambifaria IOP40-10	assembly	Betaproteobacteria	No	-	Yes	Aerobic
Burkholderia ambifaria MC40-6	complete	Betaproteobacteria		-		
Burkholderia ambifaria MEX-5	assembly	Betaproteobacteria	No	-	Yes	Aerobic
Burkholderia cenocepacia AU 1054	complete	Betaproteobacteria		_		
Burkholderia cenocepacia HI2424	complete	Betaproteobacteria		_		
Burkholderia cenocepacia J2315	complete	Betaproteobacteria		-	Yes	Facultative
Burkholderia cenocepacia MC0-3	complete	Betaproteobacteria	No	-	Yes	Facultative
Burkholderia graminis C4D1M	assembly	Betaproteobacteria	No	-	Yes	Aerobic
Burkholderia mallei ATCC 23344	complete	Betaproteobacteria		-	No	
Burkholderia mallei NCTC 10229	complete	Betaproteobacteria		-	No	
Burkholderia mallei NCTC 10247	complete	Betaproteobacteria		-	No	
Burkholderia mallei SAVP1	complete	Betaproteobacteria		-	No	
Burkholderia multivorans ATCC 17616	complete	Betaproteobacteria		-		Aerobic
Burkholderia multivorans CGD1	assembly	Betaproteobacteria		-		Aerobic
Burkholderia multivorans CGD2	assembly	Betaproteobacteria		-		Aerobic
Burkholderia multivorans CGD2M	assembly	Betaproteobacteria		-		Aerobic
Burkholderia phymatum STM815	complete	Betaproteobacteria	No	-	Yes	
Burkholderia phytofirmans PsJN	complete	Betaproteobacteria		-	Yes	Aerobic
Burkholderia pseudomallei 1106a	complete	Betaproteobacteria		-	Yes	Aerobic
Burkholderia pseudomallei 1710b	complete	Betaproteobacteria		-	Yes	Aerobic
Burkholderia pseudomallei 305	assembly	Betaproteobacteria		-	Yes	Aerobic
Burkholderia pseudomallei 576	assembly	Betaproteobacteria		-	Yes	Aerobic
Burkholderia pseudomallei 668	complete	Betaproteobacteria		-	Yes	Aerobic
Burkholderia pseudomallei K96243	complete	Betaproteobacteria		-	Yes	Aerobic
Burkholderia sp. 383	complete	Betaproteobacteria		-	Yes	Facultative
Burkholderia sp. H160	assembly	Betaproteobacteria	No	-	Yes	Aerobic
Burkholderia thailandensis E264	complete	Betaproteobacteria		-	Yes	Aerobic
Burkholderia vietnamiensis G4	complete	Betaproteobacteria		-	Yes	Facultative
Burkholderia xenovorans LB400	complete	Betaproteobacteria	No	-	Yes	Aerobic
Caldicellulosiruptor saccharolyticus DSM 8903	complete	Other Bacteria		+		Anaerobic
Caldivirga maquilingensis IC-167	complete	Crenarchaeota	No	_	No	Microaerophilic
Caminibacter mediatlanticus TB-2	assembly	Epsilonproteobacteria		-	Yes	Microaerophilic
Campylobacter coli RM2228	assembly	Epsilonproteobacteria	No	-	Yes	Microaerophilic
Campylobacter concisus 13826	complete	Epsilonproteobacteria	No	-	Yes	Microaerophilic
Campylobacter curvus 525.92	complete	Epsilonproteobacteria	No	-	Yes	Microaerophilic
Campylobacter fetus subsp. fetus 82-40	complete	Epsilonproteobacteria		-	Yes	Microaerophilic
Campylobacter hominis ATCC BAA-381	complete	Epsilonproteobacteria	No	-	No	Anaerobic
Campylobacter jejuni RM1221	complete	Epsilonproteobacteria	No	-	Yes	Microaerophilic
Campylobacter jejuni subsp. doylei 269.97	complete	Epsilonproteobacteria	No	-	Yes	Microaerophilic
Campylobacter jejuni subsp. jejuni 260.94	assembly	Epsilonproteobacteria	No	-	Yes	
Campylobacter jejuni subsp. jejuni 81-176	assembly	Epsilonproteobacteria	No	-	Yes	Microaerophilic
Campylobacter jejuni subsp. jejuni 81116	complete	Epsilonproteobacteria	No	-	Yes	Microaerophilic
Campylobacter jejuni subsp. jejuni 84-25	assembly	Epsilonproteobacteria	No	-	Yes	

			phenotype categories			
name	*sequence status*	*taxonomic group*	*Endospores*	*Gram Stain*	*Motility*	*Oxygene Req.*
Campylobacter jejuni subsp. jejuni CF93-6	assembly	Epsilonproteobacteria		-	Yes	
Campylobacter jejuni subsp. jejuni CG8421	assembly	Epsilonproteobacteria	No	-	Yes	Microaerophilic
Campylobacter jejuni subsp. jejuni CG8486	assembly	Epsilonproteobacteria	No	-	Yes	Microaerophilic
Campylobacter jejuni subsp. jejuni HB93-13	assembly	Epsilonproteobacteria	No	-	Yes	
Campylobacter jejuni subsp. jejuni NCTC 11168	complete	Epsilonproteobacteria	No	-	Yes	Microaerophilic
Campylobacter lari RM2100	complete	Epsilonproteobacteria	No	-	Yes	Microaerophilic
Campylobacter rectus RM3267	assembly	Epsilonproteobacteria		-	Motile	
Campylobacter upsaliensis RM3195	assembly	Epsilonproteobacteria	No	-	Yes	
Candidatus Amoebophilus asiaticus 5a2	complete	Bacteroidetes/Chlorobi		-		
Candidatus Azobacteroides pseudotrichonymphae genomovar. CFP2	complete	Bacteroidetes/Chlorobi		_		
Candidatus Blochmannia floridanus	complete	Gammaproteobacteria		-		
Candidatus Blochmannia pennsylvanicus str. BPEN	complete	Gammaproteobacteria		_		
Candidatus Carsonella ruddii PV	complete	Gammaproteobacteria		_		
Candidatus Desulforudis audaxviator MP104C	complete	Firmicutes		+	Yes	
Candidatus Korarchaeum cryptofilum OPF8	complete	Other Archaea		_		
Candidatus Methanoregula boonei 6A8	complete	Euryarchaeota		_		Anaerobic
Candidatus Methanosphaerula palustris E1-9c	complete	Euryarchaeota		_	No	Anaerobic
Candidatus Pelagibacter ubique HTCC1062	complete	Alphaproteobacteria		-	No	Aerobic
Candidatus Phytoplasma australiense	complete	Firmicutes		_		Aerobic
Candidatus Phytoplasma mali	complete	Other Bacteria		_		Aerobic
Candidatus Protochlamydia amoebophila UWE25	complete	Chlamydiae/Verrucomicrobia		-		
Candidatus Ruthia magnifica str. Cm (Calyptogena magnifica)	complete	Gammaproteobacteria		_		
Candidatus Sulcia muelleri GWSS	complete	Bacteroidetes/Chlorobi		_		
Candidatus Vesicomyosocius okutanii HA	complete	Gammaproteobacteria		_		Anaerobic
Carboxydothermus hydrogenoformans Z-2901	complete	Firmicutes		+		Anaerobic
Carnobacterium sp. AT7	assembly	Firmicutes		+		
Caulobacter crescentus CB15	complete	Alphaproteobacteria		-	Yes	Aerobic
Caulobacter crescentus NA1000	complete	Alphaproteobacteria		-	yes	Aerobic
Caulobacter sp. K31	complete	Alphaproteobacteria		-	Yes	Aerobic
Cellvibrio japonicus Ueda107	complete	Gammaproteobacteria		-	Yes	Aerobic
Chlamydia muridarum Nigg	complete	Chlamydiae/Verrucomicrobia		-		
Chlamydia trachomatis 434/Bu	complete	Chlamydiae/Verrucomicrobia		-		
Chlamydia trachomatis A/HAR-13	complete	Chlamydiae/Verrucomicrobia		-		
Chlamydia trachomatis D/UW-3/CX	complete	Chlamydiae/Verrucomicrobia		-		
Chlamydia trachomatis L2b/UCH-1/proctitis	complete	Chlamydiae/Verrucomicrobia		-		
Chlamydophila abortus S26/3	complete	Chlamydiae/Verrucomicrobia		-		
Chlamydophila caviae GPIC	complete	Chlamydiae/Verrucomicrobia		-		
Chlamydophila felis Fe/C-56	complete	Chlamydiae/Verrucomicrobia		-		
Chlamydophila pneumoniae AR39	complete	Chlamydiae/Verrucomicrobia		-		
Chlamydophila pneumoniae CWL029	complete	Chlamydiae/Verrucomicrobia		-		
Chlamydophila pneumoniae J138	complete	Chlamydiae/Verrucomicrobia		-		
Chlamydophila pneumoniae TW-183	complete	Chlamydiae/Verrucomicrobia		-		
Chlorobaculum parvum NCIB 8327	complete	Bacteroidetes/Chlorobi		_		Facultative
Chlorobium chlorochromatii CaD3	complete	Bacteroidetes/Chlorobi		-	No	Anaerobic
Chlorobium ferrooxidans DSM 13031	assembly	Bacteroidetes/Chlorobi		-	No	Anaerobic
Chlorobium limicola DSM 245	complete	Bacteroidetes/Chlorobi		-	No	Anaerobic
Chlorobium phaeobacteroides BS1	complete	Bacteroidetes/Chlorobi	No	-	No	
Chlorobium phaeobacteroides DSM 266	complete	Bacteroidetes/Chlorobi	No	-	No	Facultative
Chlorobium phaeovibrioides DSM 265	complete	Bacteroidetes/Chlorobi	No	-	No	Facultative
Chlorobium tepidum TLS	complete	Bacteroidetes/Chlorobi		_		Anaerobic
Chloroflexus aggregans DSM 9485	complete	Chloroflexi		_	Yes	Facultative
Chloroflexus aurantiacus J-10-fl	complete	Chloroflexi		_	Yes	Anaerobic
Chloroflexus sp. Y-400-fl	complete	Chloroflexi		_		
Chloroherpeton thalassium ATCC 35110	complete	Bacteroidetes/Chlorobi		-	Yes	Facultative
Chromobacterium violaceum ATCC 12472	complete	Betaproteobacteria		-	Yes	Facultative
Chromohalobacter salexigens DSM 3043	unfinished	Gammaproteobacteria		-	Yes	Facultative
Chthoniobacter flavus Ellin428	assembly	Other Bacteria		-	No	Aerobic
Citrobacter koseri ATCC BAA-895	complete	Gammaproteobacteria		-		
Clavibacter michiganensis subsp. michiganensis NCPPB 382	complete	Actinobacteria	No	+	No	Aerobic
Clavibacter michiganensis subsp. sepedonicus	complete	Actinobacteria	No	+	No	Aerobic
Clostridium acetobutylicum ATCC 824	complete	Firmicutes	Yes	+	Yes	Anaerobic
Clostridium beijerinckii NCIMB 8052	complete	Firmicutes	Yes	+	Yes	
Clostridium botulinum A str. ATCC 19397	complete	Firmicutes	Yes	+	Yes	Anaerobic
Clostridium botulinum A str. ATCC 3502	complete	Firmicutes	Yes	+	Yes	Anaerobic
Clostridium botulinum A str. Hall	complete	Firmicutes	Yes	+	Yes	Anaerobic
Clostridium botulinum A3 str. Loch Maree	complete	Firmicutes	Yes	+	Yes	Anaerobic
Clostridium botulinum B str. Eklund 17B	complete	Firmicutes	Yes	+	Yes	Anaerobic
Clostridium botulinum B1 str. Okra	complete	Firmicutes	Yes	+	Yes	Anaerobic
Clostridium botulinum Bf	assembly	Firmicutes	Yes	+	Yes	Anaerobic
Clostridium botulinum C str. Eklund	assembly	Firmicutes	Yes	+	Yes	Anaerobic
Clostridium botulinum E3 str. Alaska E43	complete	Firmicutes	Yes	+	Yes	Anaerobic
Clostridium botulinum F str. Langeland	complete	Firmicutes	Yes	+	Yes	Anaerobic
Clostridium botulinum NCTC 2916	assembly	Firmicutes	Yes	+	Yes	Anaerobic
Clostridium butyricum 5521	assembly	Firmicutes	Yes	+	Yes	Anaerobic
Clostridium cellulolyticum H10	complete	Firmicutes	Yes	+	Yes	Anaerobic
Clostridium difficile 630	complete	Firmicutes	Yes	+	Yes	Anaerobic
Clostridium kluyveri DSM 555	complete	Firmicutes	Yes	+	Yes	Anaerobic
Clostridium novyi NT	complete	Firmicutes	Yes	+	Yes	Anaerobic
Clostridium perfringens ATCC 13124	complete	Firmicutes	Yes	+	No	Anaerobic
Clostridium perfringens B str. ATCC 3626	assembly	Firmicutes	Yes	+	No	Anaerobic
Clostridium perfringens C str. JGS1495	assembly	Firmicutes	Yes	+	No	Anaerobic
Clostridium perfringens CPE str. F4969	assembly	Firmicutes	Yes	+	No	Anaerobic
Clostridium perfringens D str. JGS1721	assembly	Firmicutes	Yes	+	Yes	Anaerobic
Clostridium perfringens E str. JGS1987	assembly	Firmicutes	Yes	+	No	Anaerobic
Clostridium perfringens NCTC 8239	assembly	Firmicutes	Yes	+	No	Anaerobic
Clostridium perfringens SM101	complete	Firmicutes	Yes	+	No	Anaerobic
Clostridium perfringens str. 13	complete	Firmicutes	Yes	+	No	Anaerobic
Clostridium phytofermentans ISDg	complete	Firmicutes	Yes	+	Yes	Anaerobic
Clostridium tetani E88	complete	Firmicutes	No	+	Yes	Anaerobic
Clostridium thermocellum ATCC 27405	complete	Firmicutes	Yes	+	Yes	Anaerobic
Clostridium thermocellum DSM 4150	assembly	Firmicutes	Yes	+	Yes	Anaerobic
Collinsella aerofaciens ATCC 25986	assembly	Actinobacteria	No	+	No	Anaerobic
Colwellia psychrerythraea 34H	complete	Gammaproteobacteria		-	Yes	Facultative
Comamonas testosteroni KF-1	assembly	Betaproteobacteria	No	-	Yes	Aerobic
Coprothermobacter proteolyticus DSM 5265	complete	Firmicutes	No	-	No	Anaerobic

			phenotype categories			
name	*sequence status*	*taxonomic group*	*Endospores*	*Gram Stain*	*Motility*	*Oxygene Req.*
Corynebacterium diphtheriae NCTC 13129	complete	Actinobacteria	No	+	No	Aerobic
Corynebacterium efficiens YS-314	complete	Actinobacteria	No	+	No	Facultative
Corynebacterium glutamicum ATCC 13032	complete	Actinobacteria	No	+	No	Facultative
Corynebacterium glutamicum R	complete	Actinobacteria	No	+	No	Facultative
Corynebacterium jeikeium K411	complete	Actinobacteria	No	+	No	Facultative
Corynebacterium urealyticum DSM 7109	complete	Actinobacteria	No	+	No	Aerobic
Coxiella burnetii CbuG_Q212	complete	Gammaproteobacteria		-		Facultative
Coxiella burnetii CbuK_Q154	complete	Gammaproteobacteria		-		Facultative
Coxiella burnetii Dugway 5J108-111	complete	Gammaproteobacteria		-		Facultative
Coxiella burnetii RSA 331	complete	Gammaproteobacteria		-		Facultative
Coxiella burnetii RSA 334	assembly	Gammaproteobacteria		-		Facultative
Coxiella burnetii RSA 493	complete	Gammaproteobacteria		-		Facultative
Crocosphaera watsonii WH 8501	assembly	Cyanobacteria		_		
Cronobacter sakazakii ATCC BAA-894	complete	Gammaproteobacteria	No	-	Yes	Anaerobic
Cupriavidus taiwanensis	complete	Betaproteobacteria	No	-	Yes	Facultative
Cyanothece sp. ATCC 51142	complete	Cyanobacteria		_		Facultative
Cyanothece sp. CCY 0110	assembly	Cyanobacteria		_		Facultative
Cyanothece sp. PCC 7424	complete	Cyanobacteria		_	No	Facultative
Cyanothece sp. PCC 7425	complete	Cyanobacteria		_	No	Facultative
Cyanothece sp. PCC 7822	assembly	Cyanobacteria		_	No	Anaerobic
Cyanothece sp. PCC 8801	complete	Cyanobacteria		_		Facultative
Cyanothece sp. PCC 8802	assembly	Cyanobacteria		_	No	Facultative
Cytophaga hutchinsonii ATCC 33406	complete	Bacteroidetes/Chlorobi		-	Yes	Aerobic
Dechloromonas aromatica RCB	complete	Betaproteobacteria	No	-	Yes	Facultative
Dehalococcoides ethenogenes 195	complete	Chloroflexi	No	+	Yes	Anaerobic
Dehalococcoides sp. BAV1	complete	Chloroflexi		_		Anaerobic
Dehalococcoides sp. CBDB1	complete	Chloroflexi	No	+	No	Anaerobic
Dehalococcoides sp. VS	assembly	Chloroflexi		_		Anaerobic
Deinococcus geothermalis DSM 11300	complete	Deinococcus-Thermus		+		Aerobic
Deinococcus radiodurans R1	complete	Deinococcus-Thermus		+		Aerobic
Delftia acidovorans SPH-1	complete	Betaproteobacteria		-		Aerobic
delta proteobacterium MLMS-1	assembly	Deltaproteobacteria		-	Yes	
Desulfatibacillum alkenivorans AK-01	complete	Deltaproteobacteria	No	-	No	Anaerobic
Desulfitobacterium hafniense DCB-2	complete	Firmicutes	Yes	-	Yes	Anaerobic
Desulfitobacterium hafniense Y51	complete	Firmicutes	Yes	-	Yes	Anaerobic
Desulfobacterium autotrophicum HRM2	complete	Deltaproteobacteria		-	Yes	Anaerobic
Desulfococcus oleovorans Hxd3	complete	Deltaproteobacteria		-		Anaerobic
Desulfonatronospira thiodismutans ASO3-1	assembly	Deltaproteobacteria	No	-	Yes	Anaerobic
Desulfotalea psychrophila LSv54	complete	Deltaproteobacteria		-		Anaerobic
Desulfotomaculum reducens MI-1	complete	Firmicutes	Yes	+	Yes	Anaerobic
Desulfovibrio desulfuricans subsp. desulfuricans str. ATCC 27774	complete	Deltaproteobacteria	No	-	Yes	Anaerobic
Desulfovibrio desulfuricans subsp. desulfuricans str. G20	complete	Deltaproteobacteria		-	Yes	Anaerobic
Desulfovibrio salexigens DSM 2638	assembly	Deltaproteobacteria	No	-		Anaerobic
Desulfovibrio vulgaris DP4	complete	Deltaproteobacteria		-		Anaerobic
Desulfovibrio vulgaris str. 'Miyazaki F'	complete	Deltaproteobacteria		-		Anaerobic
Desulfovibrio vulgaris str. Hildenborough	complete	Deltaproteobacteria		-	Yes	Anaerobic
Desulfurococcus kamchatkensis 1221n	complete	Crenarchaeota		_		Anaerobic
Desulfuromonas acetoxidans DSM 684	assembly	Deltaproteobacteria		-	Yes	Anaerobic
Dethiobacter alkaliphilus AHT 1	assembly	Firmicutes	Yes	_	Yes	Anaerobic
Diaphorobacter sp. TPSY	complete	Betaproteobacteria		-	Yes	Facultative
Dichelobacter nodosus VCS1703A	complete	Gammaproteobacteria		-		Anaerobic
Dictyoglomus thermophilum H-6-12	complete	Other Bacteria	No	-	No	Anaerobic
Dictyoglomus turgidum DSM 6724	complete	Other Bacteria		+		Anaerobic
Dinoroseobacter shibae DFL 12	complete	Alphaproteobacteria		-	Yes	Aerobic
Ehrlichia canis str. Jake	complete	Alphaproteobacteria		-		
Ehrlichia chaffeensis str. Arkansas	complete	Alphaproteobacteria		_		
Ehrlichia chaffeensis str. Sapulpa	assembly	Alphaproteobacteria		_		
Ehrlichia ruminantium str. Gardel	complete	Alphaproteobacteria		-		
Ehrlichia ruminantium str. Welgevonden	complete	Alphaproteobacteria		-		
Elusimicrobium minutum Pei191	complete	Other Bacteria		_		Anaerobic
Enterobacter sp. 638	complete	Gammaproteobacteria		_		
Enterococcus faecalis V583	complete	Firmicutes		+		Facultative
Enterococcus faecium DO	assembly	Firmicutes		+		Facultative
Erythrobacter litoralis HTCC2594	complete	Alphaproteobacteria	No	-	Yes	Aerobic
Erythrobacter sp. SD-21	assembly	Alphaproteobacteria		-	Yes	Aerobic
Escherichia coli 101-1	assembly	Gammaproteobacteria		-	Yes	Facultative
Escherichia coli 536	complete	Gammaproteobacteria		-	Yes	Facultative
Escherichia coli APEC O1	complete	Gammaproteobacteria		-	Yes	Facultative
Escherichia coli ATCC 8739	complete	Gammaproteobacteria		-		Facultative
Escherichia coli B7A	assembly	Gammaproteobacteria		-	Yes	Facultative
Escherichia coli E110019	assembly	Gammaproteobacteria		-	Yes	Facultative
Escherichia coli E22	assembly	Gammaproteobacteria		-	Yes	Facultative
Escherichia coli E24377A	complete	Gammaproteobacteria		-	Yes	Facultative
Escherichia coli F11	assembly	Gammaproteobacteria		-	Yes	Facultative
Escherichia coli HS	complete	Gammaproteobacteria		-	Yes	Facultative
Escherichia coli O127:H6 str. E2348/69	complete	Gammaproteobacteria	No	-	Yes	Facultative
Escherichia coli O157:H7 EDL933	complete	Gammaproteobacteria		-	Yes	Facultative
Escherichia coli O157:H7 str. EC4076	assembly	Gammaproteobacteria	No	-	Yes	Facultative
Escherichia coli O157:H7 str. EC4113	assembly	Gammaproteobacteria	No	-	Yes	Facultative
Escherichia coli O157:H7 str. EC4115	complete	Gammaproteobacteria	No	-	Yes	Facultative
Escherichia coli O157:H7 str. EC4196	assembly	Gammaproteobacteria	No	-	Yes	Facultative
Escherichia coli O157:H7 str. EC4401	assembly	Gammaproteobacteria	No	-	Yes	Facultative
Escherichia coli O157:H7 str. EC4486	assembly	Gammaproteobacteria	No	-	Yes	Facultative
Escherichia coli O157:H7 str. EC4501	assembly	Gammaproteobacteria	No	-	Yes	Facultative
Escherichia coli O157:H7 str. EC508	assembly	Gammaproteobacteria	No	-	Yes	Facultative
Escherichia coli O157:H7 str. EC869	assembly	Gammaproteobacteria	No	-	Yes	Facultative
Escherichia coli O157:H7 str. Sakai	complete	Gammaproteobacteria		-	Yes	Facultative
Escherichia coli SE11	complete	Gammaproteobacteria		-		Facultative
Escherichia coli SMS-3-5	complete	Gammaproteobacteria	No	-	Yes	Facultative
Escherichia coli str. K-12 substr. DH10B	complete	Gammaproteobacteria		-	Yes	Facultative
Escherichia coli str. K-12 substr. MG1655	complete	Gammaproteobacteria		-	Yes	Facultative
Escherichia coli str. K-12 substr. W3110	complete	Gammaproteobacteria		-	Yes	Facultative
Escherichia coli UTI89	complete	Gammaproteobacteria		-	Yes	Facultative
Escherichia fergusonii ATCC 35469	complete	Gammaproteobacteria	No	-	Yes	Facultative
Eubacterium hallii DSM 3353	assembly	Firmicutes		+		Anaerobic

				phenotype categories		
name	sequence status	taxonomic group	Endospores	Gram Stain	Motility	Oxygene Req.
Exiguobacterium sibiricum 255-15	complete	Firmicutes	No	+		Facultative
Exiguobacterium sp. AT1b	assembly	Firmicutes		+		
Fervidobacterium nodosum Rt17-B1	complete	Thermotogae	No	-	Yes	Anaerobic
Finegoldia magna ATCC 29328	complete	Firmicutes		+		Anaerobic
Flavobacteria bacterium BAL38	assembly	Bacteroidetes/Chlorobi		_	Yes	Aerobic
Flavobacteria bacterium MS024-2A	assembly	Bacteroidetes/Chlorobi		_		
Flavobacteriales bacterium ALC-1	assembly	Bacteroidetes/Chlorobi		_		
Flavobacterium johnsoniae UW101	complete	Bacteroidetes/Chlorobi	No	-	Yes	Aerobic
Flavobacterium psychrophilum JIP02/86	complete	Bacteroidetes/Chlorobi	No	-	Yes	Aerobic
Francisella novicida U112	complete	Gammaproteobacteria		-	No	Aerobic
Francisella philomiragia subsp. philomiragia ATCC 25017	complete	Gammaproteobacteria	No	-	No	Aerobic
Francisella tularensis subsp. holarctica FTNF002-00	complete	Gammaproteobacteria		-	No	Aerobic
Francisella tularensis subsp. holarctica OSU18	complete	Gammaproteobacteria		-	No	Aerobic
Francisella tularensis subsp. mediasiatica FSC147	complete	Gammaproteobacteria		-	No	Aerobic
Francisella tularensis subsp. tularensis FSC198	complete	Gammaproteobacteria		-	No	Aerobic
Francisella tularensis subsp. tularensis SCHU S4	complete	Gammaproteobacteria		-	No	Aerobic
Francisella tularensis subsp. tularensis WY96-3418	complete	Gammaproteobacteria		-	No	Aerobic
Frankia alni ACN14a	complete	Actinobacteria		+		
Frankia sp. CcI3	complete	Actinobacteria		+		Aerobic
Frankia sp. EAN1pec	complete	Actinobacteria		+		Aerobic
Fusobacterium nucleatum subsp. nucleatum ATCC 25586	complete	Fusobacteria	No	-	No	Anaerobic
Fusobacterium nucleatum subsp. polymorphum ATCC 10953	assembly	Fusobacteria	No	-	No	Anaerobic
Fusobacterium nucleatum subsp. vincentii ATCC 49256	assembly	Fusobacteria	No	-	No	Anaerobic
Geobacillus kaustophilus HTA426	complete	Firmicutes	Yes	+	No	Aerobic
Geobacillus sp. G11MC16	assembly	Firmicutes	Yes	+	Yes	Facultative
Geobacillus sp. WCH70	assembly	Firmicutes	Yes	+	Yes	Facultative
Geobacillus sp. Y412MC10	assembly	Firmicutes	Yes	+	Yes	Facultative
Geobacillus sp. Y412MC61	assembly	Firmicutes	Yes	+	Yes	Facultative
Geobacillus thermodenitrificans NG80-2	complete	Firmicutes	Yes	+	Yes	Facultative
Geobacter bemidjiensis Bem	complete	Deltaproteobacteria		-	No	Anaerobic
Geobacter lovleyi SZ	complete	Deltaproteobacteria	No	-	Yes	Anaerobic
Geobacter metallireducens GS-15	complete	Deltaproteobacteria		-	Yes	Anaerobic
Geobacter sp. FRC-32	complete	Deltaproteobacteria	No	-		Anaerobic
Geobacter sp. M21	assembly	Deltaproteobacteria		-	Yes	Anaerobic
Geobacter sulfurreducens PCA	complete	Deltaproteobacteria		-	Yes	Anaerobic
Geobacter uraniireducens Rf4	complete	Deltaproteobacteria	No	-		Microaerophilic
Gloeobacter violaceus PCC 7421	complete	Cyanobacteria		_		
Gluconacetobacter diazotrophicus PAl 5	complete	Alphaproteobacteria	No	-	Yes	Aerobic
Gluconobacter oxydans 621H	complete	Alphaproteobacteria		-	Yes	Aerobic
Gramella forsetii KT0803	complete	Bacteroidetes/Chlorobi		-		Aerobic
Granulibacter bethesdensis CGDNIH1	complete	Alphaproteobacteria		-		
Haemophilus ducreyi 35000HP	complete	Gammaproteobacteria		-		Anaerobic
Haemophilus influenzae 22.1-21	assembly	Gammaproteobacteria		-		Facultative
Haemophilus influenzae 22.4-21	assembly	Gammaproteobacteria		-		Facultative
Haemophilus influenzae 3655	assembly	Gammaproteobacteria		-		Facultative
Haemophilus influenzae 86-028NP	complete	Gammaproteobacteria		-		Facultative
Haemophilus influenzae PittAA	assembly	Gammaproteobacteria		-		Facultative
Haemophilus influenzae PittEE	complete	Gammaproteobacteria		-		Facultative
Haemophilus influenzae PittGG	complete	Gammaproteobacteria		-		Facultative
Haemophilus influenzae PittHH	assembly	Gammaproteobacteria		-		Facultative
Haemophilus influenzae PittII	assembly	Gammaproteobacteria		-		Facultative
Haemophilus influenzae R3021	assembly	Gammaproteobacteria		-		Facultative
Haemophilus influenzae Rd KW20	complete	Gammaproteobacteria		-	No	Facultative
Haemophilus parasuis 29755	assembly	Gammaproteobacteria		-	No	Facultative
Haemophilus parasuis SH0165	complete	Gammaproteobacteria		-	No	Facultative
Haemophilus somnus 129PT	complete	Gammaproteobacteria		-		Facultative
Haemophilus somnus 2336	complete	Gammaproteobacteria		-		Facultative
Hahella chejuensis KCTC 2396	complete	Gammaproteobacteria		-	Yes	Facultative
Haloarcula marismortui ATCC 43049	complete	Euryarchaeota	No	_	Yes	Aerobic
Halobacterium salinarum R1	complete	Euryarchaeota	No	_	Yes	Anaerobic
Halobacterium sp. NRC-1	complete	Euryarchaeota	No	-	Yes	Facultative
Haloquadratum walsbyi DSM 16790	complete	Euryarchaeota		_		
Halorhodospira halophila SL1	complete	Gammaproteobacteria		-	Yes	
Halorubrum lacusprofundi ATCC 49239	complete	Euryarchaeota		_	Yes	Aerobic
Halothermothrix orenii H 168	complete	Firmicutes		-		Anaerobic
Halothiobacillus neapolitanus c2	assembly	Gammaproteobacteria		-	Yes	Aerobic
Helicobacter acinonychis str. Sheeba	complete	Epsilonproteobacteria		-	Yes	Microaerophilic
Helicobacter hepaticus ATCC 51449	complete	Epsilonproteobacteria		-	Yes	Aerobic
Helicobacter pylori 26695	complete	Epsilonproteobacteria		-	Yes	Aerobic
Helicobacter pylori 98-10	assembly	Epsilonproteobacteria	No	-	Yes	Microaerophilic
Helicobacter pylori B128	assembly	Epsilonproteobacteria	No	-	Yes	Microaerophilic
Helicobacter pylori G27	complete	Epsilonproteobacteria	No	-	Yes	Microaerophilic
Helicobacter pylori HPAG1	complete	Epsilonproteobacteria		-	Yes	Aerobic
Helicobacter pylori J99	complete	Epsilonproteobacteria		-	Yes	Aerobic
Helicobacter pylori P12	complete	Epsilonproteobacteria	No	-	Yes	Microaerophilic
Helicobacter pylori Shi470	complete	Epsilonproteobacteria		-	Yes	Aerobic
Heliobacterium modesticaldum Ice1	complete	Firmicutes	Yes	-	Yes	Anaerobic
Herminiimonas arsenicoxydans	complete	Betaproteobacteria		_		Anaerobic
Herpetosiphon aurantiacus ATCC 23779	complete	Chloroflexi		-	Yes	Aerobic
Hydrogenivirga sp. 128-5-R1-1	assembly	Aquificae		-	Yes	Anaerobic
Hydrogenobaculum sp. Y04AAS1	complete	Aquificae	No	-	Yes	Aerobic
Hyperthermus butylicus DSM 5456	complete	Crenarchaeota	No	_	Yes	Anaerobic
Hyphomonas neptunium ATCC 15444	complete	Alphaproteobacteria		-	Yes	Aerobic
Idiomarina loihiensis L2TR	complete	Gammaproteobacteria		-	Yes	Aerobic
Ignicoccus hospitalis KIN4/I	complete	Crenarchaeota	No	-	Yes	Anaerobic
Jannaschia sp. CCS1	complete	Alphaproteobacteria	No	-	No	Aerobic
Janthinobacterium sp. Marseille	complete	Betaproteobacteria		_	Yes	
Kineococcus radiotolerans SRS30216	complete	Actinobacteria	No	+	Yes	Aerobic
Klebsiella pneumoniae 342	complete	Gammaproteobacteria		-	Yes	Facultative
Klebsiella pneumoniae subsp. pneumoniae MGH 78578	complete	Gammaproteobacteria		-	Yes	Facultative
Kocuria rhizophila DC2201	complete	Actinobacteria		+	No	Aerobic
Labrenzia aggregata IAM 12614	assembly	Alphaproteobacteria		-	Yes	
Lactobacillus acidophilus NCFM	complete	Firmicutes		+		Facultative
Lactobacillus brevis ATCC 367	complete	Firmicutes		+		Facultative
Lactobacillus casei ATCC 334	complete	Firmicutes		+		Facultative

			phenotype categories			
name	*sequence status*	*taxonomic group*	*Endospores*	*Gram Stain*	*Motility*	*Oxygene Req.*
Lactobacillus casei BL23	complete	Firmicutes		+		Facultative
Lactobacillus delbrueckii subsp. bulgaricus ATCC 11842	complete	Firmicutes		+		Facultative
Lactobacillus delbrueckii subsp. bulgaricus ATCC BAA-365	complete	Firmicutes		+		Facultative
Lactobacillus fermentum IFO 3956	complete	Firmicutes		+		Facultative
Lactobacillus gasseri ATCC 33323	complete	Firmicutes		+		Facultative
Lactobacillus helveticus DPC 4571	complete	Firmicutes		+		Facultative
Lactobacillus johnsonii NCC 533	complete	Firmicutes		+		Facultative
Lactobacillus plantarum WCFS1	complete	Firmicutes		+		Facultative
Lactobacillus reuteri 100-23	assembly	Firmicutes	No	+		Facultative
Lactobacillus sakei subsp. sakei 23K	complete	Firmicutes		+		Facultative
Lactobacillus salivarius UCC118	complete	Firmicutes	No	+		Facultative
Lactococcus lactis subsp. cremoris MG1363	complete	Firmicutes		+		Facultative
Lactococcus lactis subsp. cremoris SK11	complete	Firmicutes		+		Facultative
Lactococcus lactis subsp. lactis Il1403	complete	Firmicutes		+		Facultative
Lawsonia intracellularis PHE/MN1-00	complete	Deltaproteobacteria		-		Facultative
Legionella pneumophila str. Corby	complete	Gammaproteobacteria		-	Yes	Aerobic
Legionella pneumophila str. Lens	complete	Gammaproteobacteria		-	Yes	Aerobic
Legionella pneumophila str. Paris	complete	Gammaproteobacteria		-	Yes	Aerobic
Legionella pneumophila subsp. pneumophila str. Philadelphia 1	complete	Gammaproteobacteria		-	Yes	Aerobic
Leifsonia xyli subsp. xyli str. CTCB07	complete	Actinobacteria	No	-	No	Aerobic
Lentisphaera araneosa HTCC2155	assembly	Other Bacteria	No	-	No	
Leptospira biflexa serovar Patoc strain 'Patoc 1 (Ames)'	complete	Spirochaetes	No	-	Yes	Aerobic
Leptospira biflexa serovar Patoc strain 'Patoc 1 (Paris)'	complete	Spirochaetes	No	-	Yes	Aerobic
Leptospira borgpetersenii serovar Hardjo-bovis JB197	complete	Spirochaetes	No	-	Yes	Aerobic
Leptospira borgpetersenii serovar Hardjo-bovis L550	complete	Spirochaetes	No	-	Yes	Aerobic
Leptospira interrogans serovar Copenhageni str. Fiocruz L1-130	complete	Spirochaetes	No	_	Yes	Aerobic
Leptospira interrogans serovar Lai str. 56601	complete	Spirochaetes	No	_	Yes	Aerobic
Leptothrix cholodnii SP-6	complete	Betaproteobacteria		_	No	Aerobic
Leuconostoc citreum KM20	complete	Firmicutes		+	No	Facultative
Leuconostoc mesenteroides subsp. mesenteroides ATCC 8293	complete	Firmicutes		+		Facultative
Limnobacter sp. MED105	assembly	Betaproteobacteria		-	Yes	Aerobic
Listeria innocua Clip11262	complete	Firmicutes		+	Yes	Facultative
Listeria monocytogenes EGD-e	complete	Firmicutes		+	Yes	Facultative
Listeria monocytogenes HCC23	complete	Firmicutes		+	Yes	Facultative
Listeria monocytogenes str. 1/2a F6854	assembly	Firmicutes		+	Yes	Facultative
Listeria monocytogenes str. 4b F2365	complete	Firmicutes		+	Yes	Facultative
Listeria monocytogenes str. 4b H7858	assembly	Firmicutes		+	Yes	Facultative
Listeria welshimeri serovar 6b str. SLCC5334	complete	Firmicutes	No	+	Yes	Facultative
Lutiella nitroferrum 2002	assembly	Betaproteobacteria		_		
Lyngbya sp. PCC 8106	assembly	Cyanobacteria		-		
Lysinibacillus sphaericus C3-41	complete	Firmicutes	Yes	+	Yes	Aerobic
Magnetococcus sp. MC-1	complete	Other Bacteria		-	Yes	Facultative
Magnetospirillum magneticum AMB-1	complete	Alphaproteobacteria		-	Yes	Microaerophilic
Mannheimia succiniciproducens MBEL55E	complete	Gammaproteobacteria	No	-	No	Anaerobic
Maricaulis maris MCS10	complete	Alphaproteobacteria	No	-	Yes	Facultative
marine gamma proteobacterium HTCC2080	assembly	Gammaproteobacteria		_		
marine gamma proteobacterium HTCC2143	assembly	Gammaproteobacteria		_		
Marinobacter algicola DG893	assembly	Gammaproteobacteria	No	-	Yes	Facultative
Marinobacter aquaeolei VT8	complete	Gammaproteobacteria		-	Yes	Facultative
Marinobacter sp. ELB17	assembly	Gammaproteobacteria		-		Facultative
Marinomonas sp. MWYL1	complete	Gammaproteobacteria		-	Yes	Aerobic
Mesoplasma florum L1	complete	Firmicutes	No	-	No	Facultative
Mesorhizobium loti MAFF303099	complete	Alphaproteobacteria		-		Aerobic
Mesorhizobium sp. BNC1	complete	Alphaproteobacteria		-	Yes	Aerobic
Metallosphaera sedula DSM 5348	complete	Crenarchaeota	No	-	No	Aerobic
Methanobrevibacter smithii ATCC 35061	complete	Euryarchaeota		+	No	Anaerobic
Methanocaldococcus jannaschii DSM 2661	complete	Euryarchaeota	No	_	Yes	Anaerobic
Methanococcoides burtonii DSM 6242	complete	Euryarchaeota	No	_	Yes	Anaerobic
Methanococcus aeolicus Nankai-3	complete	Euryarchaeota	No	_	Yes	Anaerobic
Methanococcus maripaludis C5	complete	Euryarchaeota	No	_	Yes	Anaerobic
Methanococcus maripaludis C6	complete	Euryarchaeota	No	_	Yes	Anaerobic
Methanococcus maripaludis C7	complete	Euryarchaeota	No	_	Yes	Anaerobic
Methanococcus vannielii SB	complete	Euryarchaeota	No	-	Yes	Anaerobic
Methanococcus voltae A3	assembly	Euryarchaeota		_	Yes	Anaerobic
Methanocorpusculum labreanum Z	complete	Euryarchaeota	No	_	No	Anaerobic
Methanoculleus marisnigri JR1	complete	Euryarchaeota	No	-	Yes	Anaerobic
Methanopyrus kandleri AV19	complete	Euryarchaeota	No	_		Anaerobic
Methanosaeta thermophila PT	complete	Euryarchaeota	No	_	No	Anaerobic
Methanosarcina acetivorans C2A	complete	Euryarchaeota	No	_	No	Anaerobic
Methanosarcina barkeri str. Fusaro	complete	Euryarchaeota	No	_	No	Anaerobic
Methanosarcina mazei Go1	complete	Euryarchaeota	No	_	No	Anaerobic
Methanosphaera stadtmanae DSM 3091	complete	Euryarchaeota		_	No	Anaerobic
Methanospirillum hungatei JF-1	complete	Euryarchaeota	No	_	Yes	Anaerobic
Methanothermobacter thermautotrophicus str. Delta H	complete	Euryarchaeota	No	_	No	Anaerobic
Methylacidiphilum infernorum V4	complete	Other Bacteria		_		Aerobic
Methylibium petroleiphilum PM1	complete	Betaproteobacteria		-	Yes	Facultative
Methylobacillus flagellatus KT	complete	Betaproteobacteria		-	Yes	Aerobic
Methylobacterium chloromethanicum CM4	complete	Alphaproteobacteria		-	Yes	Aerobic
Methylobacterium extorquens PA1	complete	Alphaproteobacteria		-	Yes	Facultative
Methylobacterium nodulans ORS 2060	complete	Alphaproteobacteria	No	-	Yes	Aerobic
Methylobacterium populi BJ001	complete	Alphaproteobacteria	No	-		Aerobic
Methylobacterium radiotolerans JCM 2831	complete	Alphaproteobacteria		_		Aerobic
Methylobacterium sp. 4-46	complete	Alphaproteobacteria		-	Yes	Facultative
Methylocella silvestris BL2	complete	Alphaproteobacteria		-	No	Aerobic
Methylococcus capsulatus str. Bath	complete	Gammaproteobacteria		-		Aerobic
Micrococcus luteus NCTC 2665	assembly	Actinobacteria	No	+		Aerobic
Microcystis aeruginosa NIES-843	complete	Cyanobacteria		_	No	Aerobic
Microscilla marina ATCC 23134	assembly	Bacteroidetes/Chlorobi		-	Yes	Aerobic
Moorella thermoacetica ATCC 39073	complete	Firmicutes	Yes	+		Anaerobic
Moritella sp. PE36	assembly	Gammaproteobacteria		-	Yes	Facultative
Mycobacterium abscessus	complete	Actinobacteria	No	+	No	Aerobic
Mycobacterium avium 104	complete	Actinobacteria	No	+	No	Aerobic
Mycobacterium avium subsp. paratuberculosis K-10	complete	Actinobacteria	No	+	No	Aerobic
Mycobacterium bovis AF2122/97	complete	Actinobacteria	No	+	No	Aerobic
Mycobacterium bovis BCG str. Pasteur 1173P2	complete	Actinobacteria	No	+	No	Aerobic

			phenotype categories			
name	*sequence status*	*taxonomic group*	*Endospores*	*Gram Stain*	*Motility*	*Oxygene Req.*
Mycobacterium gilvum PYR-GCK	complete	Actinobacteria	No	+	No	
Mycobacterium leprae Br4923	complete	Actinobacteria	No	+	No	Aerobic
Mycobacterium leprae TN	complete	Actinobacteria	No	+	No	Aerobic
Mycobacterium marinum M	complete	Actinobacteria	No	+	No	Aerobic
Mycobacterium smegmatis str. MC2 155	complete	Actinobacteria	No	+	No	Aerobic
Mycobacterium sp. JLS	complete	Actinobacteria		+		
Mycobacterium sp. KMS	complete	Actinobacteria		+		
Mycobacterium sp. MCS	complete	Actinobacteria	No	+	No	
Mycobacterium tuberculosis CDC1551	complete	Actinobacteria	No	+	No	Aerobic
Mycobacterium tuberculosis H37Ra	assembly	Actinobacteria	No	+	No	Aerobic
Mycobacterium tuberculosis H37Rv	complete	Actinobacteria	No	+	No	Aerobic
Mycobacterium ulcerans Agy99	complete	Actinobacteria	No	+	No	Aerobic
Mycobacterium vanbaalenii PYR-1	complete	Actinobacteria	No	+	No	Aerobic
Mycoplasma arthritidis 158L3-1	complete	Firmicutes	No	-	No	Facultative
Mycoplasma capricolum subsp. capricolum ATCC 27343	complete	Firmicutes	No	-	No	Facultative
Mycoplasma gallisepticum R	complete	Firmicutes	No	-	Yes	Facultative
Mycoplasma genitalium G37	assembly	Firmicutes	No	-	Yes	Facultative
Mycoplasma hyopneumoniae 232	complete	Firmicutes	No	-	No	Facultative
Mycoplasma hyopneumoniae 7448	complete	Firmicutes	No	-	No	Facultative
Mycoplasma hyopneumoniae J	complete	Firmicutes	No	-	No	Facultative
Mycoplasma mobile 163K	complete	Firmicutes	No	-	Yes	Facultative
Mycoplasma mycoides subsp. mycoides SC str. PG1	complete	Firmicutes	No	-	No	Facultative
Mycoplasma penetrans HF-2	complete	Firmicutes	No	-	No	Facultative
Mycoplasma pneumoniae M129	complete	Firmicutes	No	-	Yes	Facultative
Mycoplasma pulmonis UAB CTIP	complete	Firmicutes	No	-	Yes	Facultative
Mycoplasma synoviae 53	complete	Firmicutes	No	-	No	Facultative
Myxococcus xanthus DK 1622	complete	Deltaproteobacteria	Yes	-	Yes	Aerobic
Nanoarchaeum equitans Kin4-M	complete	Nanoarchaeota	No	_		Anaerobic
Natranaerobius thermophilus JW/NM-WN-LF	complete	Firmicutes	No	+	No	
Natrialba magadii ATCC 43099	assembly	Euryarchaeota		-	Yes	Aerobic
Natronomonas pharaonis DSM 2160	complete	Euryarchaeota		_		Aerobic
Nautilia profundicola AmH	complete	Epsilonproteobacteria		-	Yes	Anaerobic
Neisseria cinerea ATCC 14685	assembly	Betaproteobacteria	No	-	No	Aerobic
Neisseria flavescens NRL30031/H210	assembly	Betaproteobacteria	No	-	No	Aerobic
Neisseria gonorrhoeae FA 1090	complete	Betaproteobacteria		-		Aerobic
Neisseria gonorrhoeae NCCP11945	complete	Betaproteobacteria		-		Aerobic
Neisseria lactamica ATCC 23970	assembly	Betaproteobacteria	No	-	No	Aerobic
Neisseria meningitidis 053442	complete	Betaproteobacteria		-		Aerobic
Neisseria meningitidis FAM18	complete	Betaproteobacteria		-		Aerobic
Neisseria meningitidis MC58	complete	Betaproteobacteria		-		Aerobic
Neisseria meningitidis Z2491	complete	Betaproteobacteria		-		Aerobic
Neorickettsia sennetsu str. Miyayama	complete	Alphaproteobacteria		_		
Nitratiruptor sp. SB155-2	complete	Epsilonproteobacteria		-	No	Anaerobic
Nitrobacter hamburgensis X14	complete	Alphaproteobacteria		-	Yes	Aerobic
Nitrobacter winogradskyi Nb-255	complete	Alphaproteobacteria		-	Yes	Facultative
Nitrosococcus oceani ATCC 19707	complete	Gammaproteobacteria		_	Yes	
Nitrosomonas europaea ATCC 19718	complete	Betaproteobacteria		-	Yes	Aerobic
Nitrosomonas eutropha C91	complete	Betaproteobacteria		-	Yes	
Nitrosopumilus maritimus SCM1	complete	Crenarchaeota		_	No	Aerobic
Nitrosospira multiformis ATCC 25196	complete	Betaproteobacteria		-	Yes	Aerobic
Nocardia farcinica IFM 10152	complete	Actinobacteria	Yes	+	No	Aerobic
Nocardioides sp. JS614	complete	Actinobacteria	No	+	No	Aerobic
Nodularia spumigena CCY 9414	assembly	Cyanobacteria		_		Aerobic
Nostoc azollae 0708	assembly	Cyanobacteria		_	Yes	Aerobic
Nostoc punctiforme PCC 73102	complete	Cyanobacteria		_	Yes	Aerobic
Nostoc sp. PCC 7120	complete	Cyanobacteria		_	Yes	Aerobic
Novosphingobium aromaticivorans DSM 12444	complete	Alphaproteobacteria		-	Yes	Aerobic
Oceanibulbus indolifex HEL-45	assembly	Alphaproteobacteria		-	No	Aerobic
Oceanobacillus iheyensis HTE831	complete	Firmicutes	Yes	+	Yes	Aerobic
Ochrobactrum anthropi ATCC 49188	complete	Alphaproteobacteria		_		
Oenococcus oeni ATCC BAA-1163	assembly	Firmicutes	No	+	No	Facultative
Oenococcus oeni PSU-1	complete	Firmicutes		+		Facultative
Oligotropha carboxidovorans OM5	complete	Alphaproteobacteria		-		
Onion yellows phytoplasma OY-M	complete	Firmicutes		_		Aerobic
Opitutaceae bacterium TAV2	assembly	Other Bacteria		_		Facultative
Opitutus terrae PB90-1	complete	Other Bacteria	No	_	Yes	Anaerobic
Orientia tsutsugamushi str. Boryong	complete	Alphaproteobacteria		-		
Orientia tsutsugamushi str. Ikeda	complete	Alphaproteobacteria		-		
Paenibacillus sp. JDR-2	assembly	Firmicutes	Yes	+		Aerobic
Parabacteroides distasonis ATCC 8503	complete	Bacteroidetes/Chlorobi	No	+	No	Anaerobic
Paracoccus denitrificans PD1222	complete	Alphaproteobacteria	No	-	No	Aerobic
Parvibaculum lavamentivorans DS-1	complete	Alphaproteobacteria		-	Yes	Aerobic
Pasteurella multocida subsp. multocida str. Pm70	complete	Gammaproteobacteria		-		Facultative
Pectobacterium atrosepticum SCRI1043	complete	Gammaproteobacteria		-	Yes	Facultative
Pediococcus pentosaceus ATCC 25745	complete	Firmicutes	No	+	No	Facultative
Pedobacter sp. BAL39	assembly	Bacteroidetes/Chlorobi	No	-	No	
Pelobacter carbinolicus DSM 2380	complete	Deltaproteobacteria		-		Anaerobic
Pelobacter propionicus DSM 2379	complete	Deltaproteobacteria	No	-	No	Anaerobic
Pelodictyon luteolum DSM 273	complete	Bacteroidetes/Chlorobi	No	-	No	Anaerobic
Pelodictyon phaeoclathratiforme BU-1	complete	Bacteroidetes/Chlorobi	No	-	No	Anaerobic
Pelotomaculum thermopropionicum SI	complete	Firmicutes	Yes	_		Anaerobic
Persephonella marina EX-H1	complete	Aquificae	No	-	Yes	Microaerophilic
Petrotoga mobilis SJ95	complete	Thermotogae	No	-	Yes	Anaerobic
Phenylobacterium zucineum HLK1	complete	Alphaproteobacteria		-	Yes	Aerobic
Photorhabdus luminescens subsp. laumondii TTO1	complete	Gammaproteobacteria		-	Yes	Facultative
Picrophilus torridus DSM 9790	complete	Euryarchaeota		_		Aerobic
Planctomyces maris DSM 8797	assembly	Planctomycetes		-	Yes	Aerobic
Plesiocystis pacifica SIR-1	assembly	Deltaproteobacteria		-	Yes	Aerobic
Polaromonas naphthalenivorans CJ2	complete	Betaproteobacteria		-	No	Aerobic
Polaromonas sp. JS666	complete	Betaproteobacteria		-	No	Aerobic
Polynucleobacter necessarius subsp. asymbioticus QLW-P1DMWA-1	complete	Betaproteobacteria		-	No	Aerobic
Polynucleobacter necessarius subsp. necessarius STIR1	complete	Betaproteobacteria		-	No	Aerobic
Porphyromonas gingivalis ATCC 33277	complete	Bacteroidetes/Chlorobi	No	-	No	Anaerobic
Porphyromonas gingivalis W83	complete	Bacteroidetes/Chlorobi	No	-	No	Anaerobic
Prochlorococcus marinus str. AS9601	complete	Cyanobacteria		-		

			phenotype categories			
name	*sequence status*	*taxonomic group*	*Endospores*	*Gram Stain*	*Motility*	*Oxygene Req.*
Prochlorococcus marinus str. MIT 9211	complete	Cyanobacteria		-		
Prochlorococcus marinus str. MIT 9215	complete	Cyanobacteria		-		
Prochlorococcus marinus str. MIT 9301	complete	Cyanobacteria		_		
Prochlorococcus marinus str. MIT 9303	complete	Cyanobacteria		-	No	
Prochlorococcus marinus str. MIT 9312	complete	Cyanobacteria		-	No	
Prochlorococcus marinus str. MIT 9313	complete	Cyanobacteria		_	No	
Prochlorococcus marinus str. MIT 9515	complete	Cyanobacteria		_		
Prochlorococcus marinus str. NATL1A	complete	Cyanobacteria		-		
Prochlorococcus marinus str. NATL2A	complete	Cyanobacteria		-	No	
Prochlorococcus marinus subsp. marinus str. CCMP1375	complete	Cyanobacteria		_	No	
Prochlorococcus marinus subsp. pastoris str. CCMP1986	complete	Cyanobacteria		_	No	
Propionibacterium acnes KPA171202	complete	Actinobacteria	No	+	No	Anaerobic
Prosthecochloris aestuarii DSM 271	complete	Bacteroidetes/Chlorobi		-	No	
Pseudoalteromonas atlantica T6c	complete	Gammaproteobacteria	No	-	Yes	Aerobic
Pseudoalteromonas haloplanktis TAC125	complete	Gammaproteobacteria	No	-	Yes	Aerobic
Pseudomonas aeruginosa LESB58	complete	Gammaproteobacteria		-	Yes	Aerobic
Pseudomonas aeruginosa PA7	complete	Gammaproteobacteria		-	Yes	Aerobic
Pseudomonas aeruginosa PAO1	complete	Gammaproteobacteria		-	Yes	Aerobic
Pseudomonas aeruginosa UCBPP-PA14	complete	Gammaproteobacteria		-	Yes	Aerobic
Pseudomonas entomophila L48	complete	Gammaproteobacteria		_		
Pseudomonas fluorescens Pf-5	complete	Gammaproteobacteria		-	Yes	Aerobic
Pseudomonas fluorescens Pf0-1	complete	Gammaproteobacteria		-	Yes	Aerobic
Pseudomonas mendocina ymp	complete	Gammaproteobacteria		-	Yes	Aerobic
Pseudomonas putida F1	complete	Gammaproteobacteria		-	Yes	Aerobic
Pseudomonas putida GB-1	complete	Gammaproteobacteria		-	Yes	Aerobic
Pseudomonas putida KT2440	complete	Gammaproteobacteria		-	Yes	Aerobic
Pseudomonas putida W619	complete	Gammaproteobacteria		-	Yes	Aerobic
Pseudomonas stutzeri A1501	complete	Gammaproteobacteria	No	-	Yes	Aerobic
Pseudomonas syringae pv. phaseolicola 1448A	complete	Gammaproteobacteria		-	Yes	Aerobic
Pseudomonas syringae pv. syringae B728a	complete	Gammaproteobacteria		-	Yes	Aerobic
Pseudomonas syringae pv. tomato str. DC3000	complete	Gammaproteobacteria		-	Yes	Aerobic
Psychrobacter arcticus 273-4	complete	Gammaproteobacteria		-	No	
Psychrobacter cryohalolentis K5	complete	Gammaproteobacteria		_	No	
Psychrobacter sp. PRwf-1	complete	Gammaproteobacteria		-	No	Aerobic
Psychromonas ingrahamii 37	complete	Gammaproteobacteria	No	-	No	Anaerobic
Pyrobaculum aerophilum str. IM2	complete	Crenarchaeota	No	_	Yes	Facultative
Pyrobaculum arsenaticum DSM 13514	complete	Crenarchaeota	No	_	Yes	Anaerobic
Pyrobaculum calidifontis JCM 11548	complete	Crenarchaeota	No	_	Yes	Facultative
Pyrobaculum islandicum DSM 4184	complete	Crenarchaeota	No	_	Yes	Anaerobic
Pyrococcus abyssi GE5	complete	Euryarchaeota	No	_	Yes	Anaerobic
Pyrococcus furiosus DSM 3638	complete	Euryarchaeota	No	_	Yes	Anaerobic
Pyrococcus horikoshii OT3	complete	Euryarchaeota	No	_	Yes	Anaerobic
Ralstonia eutropha H16	complete	Betaproteobacteria		-	Yes	Facultative
Ralstonia eutropha JMP134	complete	Betaproteobacteria		_	Yes	Facultative
Ralstonia metallidurans CH34	complete	Betaproteobacteria		-		Facultative
Ralstonia pickettii 12D	assembly	Betaproteobacteria		-		Aerobic
Ralstonia pickettii 12J	complete	Betaproteobacteria		-		Aerobic
Ralstonia solanacearum GMI1000	complete	Betaproteobacteria		_		Aerobic
Ralstonia solanacearum UW551	assembly	Betaproteobacteria		_		Aerobic
Renibacterium salmoninarum ATCC 33209	complete	Actinobacteria	No	+	No	Facultative
Rhizobium etli CFN 42	complete	Alphaproteobacteria		-		Aerobic
Rhizobium etli CIAT 652	complete	Alphaproteobacteria	No	-	Yes	Aerobic
Rhizobium leguminosarum bv. trifolii WSM1325	assembly	Alphaproteobacteria		-	Yes	Aerobic
Rhizobium leguminosarum bv. trifolii WSM2304	complete	Alphaproteobacteria		-	Yes	Aerobic
Rhizobium leguminosarum bv. viciae 3841	complete	Alphaproteobacteria		-	Yes	Aerobic
Rhodobacter sphaeroides 2.4.1	unfinished	Alphaproteobacteria		_		
Rhodobacter sphaeroides ATCC 17025	complete	Alphaproteobacteria		-	Yes	Facultative
Rhodobacter sphaeroides ATCC 17029	complete	Alphaproteobacteria		-	Yes	Facultative
Rhodobacter sphaeroides KD131	complete	Alphaproteobacteria		-	Yes	Facultative
Rhodobacterales bacterium HTCC2150	assembly	Alphaproteobacteria		_		
Rhodococcus jostii RHA1	complete	Actinobacteria	No	+	No	Aerobic
Rhodoferax ferrireducens T118	complete	Betaproteobacteria		-	Yes	Facultative
Rhodopseudomonas palustris BisA53	complete	Alphaproteobacteria		-	Yes	Facultative
Rhodopseudomonas palustris BisB18	complete	Alphaproteobacteria		-	Yes	Facultative
Rhodopseudomonas palustris BisB5	complete	Alphaproteobacteria		-	Yes	Facultative
Rhodopseudomonas palustris CGA009	complete	Alphaproteobacteria		-	Yes	Facultative
Rhodopseudomonas palustris HaA2	complete	Alphaproteobacteria		-	Yes	Facultative
Rhodopseudomonas palustris TIE-1	complete	Alphaproteobacteria		-	Yes	Facultative
Rhodospirillum centenum SW	complete	Alphaproteobacteria		-	Yes	Facultative
Rhodospirillum rubrum ATCC 11170	complete	Alphaproteobacteria		-	Yes	Facultative
Rickettsia akari str. Hartford	complete	Alphaproteobacteria		-		Aerobic
Rickettsia bellii OSU 85-389	complete	Alphaproteobacteria		_		
Rickettsia bellii RML369-C	complete	Alphaproteobacteria		-		
Rickettsia canadensis str. McKiel	complete	Alphaproteobacteria		-		Aerobic
Rickettsia conorii str. Malish 7	complete	Alphaproteobacteria		-		Aerobic
Rickettsia felis URRWXCal2	complete	Alphaproteobacteria		-		
Rickettsia massiliae MTU5	complete	Alphaproteobacteria		-		Aerobic
Rickettsia prowazekii str. Madrid E	complete	Alphaproteobacteria		-		Aerobic
Rickettsia rickettsii str. 'Sheila Smith'	complete	Alphaproteobacteria		-		Aerobic
Rickettsia rickettsii str. Iowa	complete	Alphaproteobacteria		-		Aerobic
Rickettsia sibirica 246	assembly	Alphaproteobacteria		-		Aerobic
Rickettsia typhi str. Wilmington	complete	Alphaproteobacteria		-		Aerobic
Rickettsiella grylli	assembly	Gammaproteobacteria		-	No	Facultative
Roseiflexus castenholzii DSM 13941	complete	Chloroflexi		_	Yes	Facultative
Roseiflexus sp. RS-1	complete	Chloroflexi	No	-	Yes	Facultative
Roseobacter denitrificans OCh 114	complete	Alphaproteobacteria		-	Yes	
Roseobacter sp. AzwK-3b	assembly	Alphaproteobacteria		_		
Roseobacter sp. CCS2	assembly	Alphaproteobacteria		_		Anaerobic
Roseobacter sp. SK209-2-6	assembly	Alphaproteobacteria		_		
Roseovarius sp. TM1035	assembly	Alphaproteobacteria		_		
Rubrobacter xylanophilus DSM 9941	complete	Actinobacteria	No	+	No	Aerobic
Ruegeria pomeroyi DSS-3	complete	Alphaproteobacteria		-	Yes	Aerobic
Ruegeria sp. TM1040	complete	Alphaproteobacteria		-	Yes	
Saccharophagus degradans 2-40	complete	Gammaproteobacteria		-	Yes	Aerobic

name	sequence status	taxonomic group	phenotype categories			
			Endospores	Gram Stain	Motility	Oxygene Req.
Saccharopolyspora erythraea NRRL 2338	unfinished	Actinobacteria	Yes	+		Aerobic
Sagittula stellata E-37	assembly	Alphaproteobacteria	No	-	No	Aerobic
Salinibacter ruber DSM 13855	complete	Bacteroidetes/Chlorobi		-	Yes	Aerobic
Salinispora arenicola CNS-205	complete	Actinobacteria	Yes	+	No	Aerobic
Salinispora tropica CNB-440	complete	Actinobacteria	Yes	+	No	Aerobic
Salmonella enterica subsp. arizonae serovar 62:z4,z23:--	complete	Gammaproteobacteria		-	Yes	Facultative
Salmonella enterica subsp. enterica serovar 4,[5],12:i:- str. CVM23701	assembly	Gammaproteobacteria		-	Yes	Facultative
Salmonella enterica subsp. enterica serovar Agona str. SL483	complete	Gammaproteobacteria		-	Yes	Facultative
Salmonella enterica subsp. enterica serovar Choleraesuis str. SC-B67	complete	Gammaproteobacteria		-	Yes	Facultative
Salmonella enterica subsp. enterica serovar Dublin str. CT_02021853	complete	Gammaproteobacteria		-	Yes	Facultative
Salmonella enterica subsp. enterica serovar Enteritidis str. P125109	complete	Gammaproteobacteria	No	-	Yes	Facultative
Salmonella enterica subsp. enterica serovar Gallinarum str. 287/91	complete	Gammaproteobacteria	No	-	Yes	Facultative
Salmonella enterica subsp. enterica serovar Hadar str. RI_05P066	assembly	Gammaproteobacteria		-	Yes	Facultative
Salmonella enterica subsp. enterica serovar Heidelberg str. SL476	complete	Gammaproteobacteria		-	Yes	Facultative
Salmonella enterica subsp. enterica serovar Heidelberg str. SL486	assembly	Gammaproteobacteria		-	Yes	Facultative
Salmonella enterica subsp. enterica serovar Javiana str. GA_MM04042433	assembly	Gammaproteobacteria		-	Yes	Facultative
Salmonella enterica subsp. enterica serovar Kentucky str. CDC 191	assembly	Gammaproteobacteria		-	Yes	Facultative
Salmonella enterica subsp. enterica serovar Newport str. SL254	complete	Gammaproteobacteria		-	Yes	Facultative
Salmonella enterica subsp. enterica serovar Newport str. SL317	assembly	Gammaproteobacteria		-	Yes	Facultative
Salmonella enterica subsp. enterica serovar Paratyphi A str. AKU_12601	complete	Gammaproteobacteria	No	-	Yes	Facultative
Salmonella enterica subsp. enterica serovar Paratyphi A str. ATCC 9150	complete	Gammaproteobacteria		-	Yes	Facultative
Salmonella enterica subsp. enterica serovar Paratyphi B str. SPB7	complete	Gammaproteobacteria	No	-	Yes	Facultative
Salmonella enterica subsp. enterica serovar Paratyphi C strain RKS4594	complete	Gammaproteobacteria		-	Yes	Facultative
Salmonella enterica subsp. enterica serovar Saintpaul str. SARA23	assembly	Gammaproteobacteria		-	Yes	Facultative
Salmonella enterica subsp. enterica serovar Saintpaul str. SARA29	assembly	Gammaproteobacteria		-	Yes	Facultative
Salmonella enterica subsp. enterica serovar Schwarzengrund str. CVM19633	complete	Gammaproteobacteria		-	Yes	Facultative
Salmonella enterica subsp. enterica serovar Schwarzengrund str. SL480	assembly	Gammaproteobacteria		-	Yes	Facultative
Salmonella enterica subsp. enterica serovar Typhi str. Ty2	complete	Gammaproteobacteria		-	Yes	Facultative
Salmonella enterica subsp. enterica serovar Typhimurium str. LT2	complete	Gammaproteobacteria		-	Yes	Facultative
Salmonella enterica subsp. enterica serovar Weltevreden str. HI_N05-537	assembly	Gammaproteobacteria		-	Yes	Facultative
Serratia proteamaculans 568	complete	Gammaproteobacteria		_	Yes	Facultative
Shewanella amazonensis SB2B	complete	Gammaproteobacteria		-		Facultative
Shewanella baltica OS155	complete	Gammaproteobacteria		-	Yes	Facultative
Shewanella baltica OS185	complete	Gammaproteobacteria		-	Yes	Facultative
Shewanella baltica OS195	complete	Gammaproteobacteria		-	Yes	Facultative
Shewanella baltica OS223	complete	Gammaproteobacteria		-	Yes	Facultative
Shewanella benthica KT99	assembly	Gammaproteobacteria		-	No	Facultative
Shewanella denitrificans OS217	complete	Gammaproteobacteria		-	Yes	Facultative
Shewanella frigidimarina NCIMB 400	complete	Gammaproteobacteria		-	Yes	Facultative
Shewanella halifaxensis HAW-EB4	complete	Gammaproteobacteria	No	-	Yes	Facultative
Shewanella loihica PV-4	complete	Gammaproteobacteria		-	Yes	Facultative
Shewanella oneidensis MR-1	complete	Gammaproteobacteria		-	Yes	Facultative
Shewanella pealeana ATCC 700345	complete	Gammaproteobacteria		-	Yes	Facultative
Shewanella piezotolerans WP3	complete	Gammaproteobacteria		-	Yes	Facultative
Shewanella putrefaciens 200	assembly	Gammaproteobacteria		-	Yes	Facultative
Shewanella putrefaciens CN-32	complete	Gammaproteobacteria		-	Yes	Facultative
Shewanella sediminis HAW-EB3	complete	Gammaproteobacteria	No	-	Yes	Facultative
Shewanella sp. ANA-3	complete	Gammaproteobacteria		-	Yes	Facultative
Shewanella sp. MR-4	complete	Gammaproteobacteria		-	Yes	Facultative
Shewanella sp. MR-7	complete	Gammaproteobacteria		-	Yes	Facultative
Shewanella sp. W3-18-1	complete	Gammaproteobacteria		-	Yes	Facultative
Shewanella woodyi ATCC 51908	complete	Gammaproteobacteria		-	Yes	Facultative
Shigella boydii CDC 3083-94	complete	Gammaproteobacteria	No	-	Yes	Facultative
Shigella boydii Sb227	complete	Gammaproteobacteria	No	-	Yes	Facultative
Shigella dysenteriae 1012	assembly	Gammaproteobacteria		-		Facultative
Shigella dysenteriae Sd197	complete	Gammaproteobacteria	No	-	Yes	Facultative
Shigella flexneri 2a str. 2457T	complete	Gammaproteobacteria		-		Facultative
Shigella flexneri 2a str. 301	complete	Gammaproteobacteria		-		Facultative
Shigella flexneri 5 str. 8401	complete	Gammaproteobacteria		-		Facultative
Shigella sonnei Ss046	complete	Gammaproteobacteria	No	-	Yes	Facultative
Sinorhizobium medicae WSM419	complete	Alphaproteobacteria	No	-		Aerobic
Sinorhizobium meliloti 1021	complete	Alphaproteobacteria		-	Yes	Aerobic
Sodalis glossinidius str. 'morsitans'	complete	Gammaproteobacteria	No	-	No	Microaerophilic
Solibacter usitatus Ellin6076	complete	Acidobacteria		-	No	Aerobic
Sorangium cellulosum 'So ce 56'	complete	Deltaproteobacteria		-	Yes	Aerobic
Sphingomonas wittichii RW1	complete	Alphaproteobacteria		-	Yes	Aerobic
Sphingopyxis alaskensis RB2256	complete	Alphaproteobacteria	No	-		Aerobic
Staphylococcus aureus RF122	complete	Firmicutes	No	+	No	Facultative
Staphylococcus aureus subsp. aureus COL	complete	Firmicutes	No	+	No	Facultative
Staphylococcus aureus subsp. aureus JH1	complete	Firmicutes	No	+	No	Facultative
Staphylococcus aureus subsp. aureus JH9	complete	Firmicutes	No	+	No	Facultative
Staphylococcus aureus subsp. aureus MRSA252	complete	Firmicutes	No	+	No	Facultative
Staphylococcus aureus subsp. aureus MSSA476	complete	Firmicutes	No	+	No	Facultative
Staphylococcus aureus subsp. aureus Mu3	complete	Firmicutes	No	+	No	Facultative
Staphylococcus aureus subsp. aureus Mu50	unfinished	Firmicutes		_		
Staphylococcus aureus subsp. aureus MW2	complete	Firmicutes	No	+	No	Facultative
Staphylococcus aureus subsp. aureus N315	complete	Firmicutes	No	+	No	Facultative
Staphylococcus aureus subsp. aureus NCTC 8325	complete	Firmicutes	No	+	No	Facultative
Staphylococcus aureus subsp. aureus str. Newman	complete	Firmicutes	No	+	No	
Staphylococcus aureus subsp. aureus USA300_FPR3757	complete	Firmicutes	No	+	No	Facultative
Staphylococcus aureus subsp. aureus USA300_TCH1516	unfinished	Firmicutes		_		
Staphylococcus capitis SK14	assembly	Firmicutes		_		
Staphylococcus carnosus subsp. carnosus TM300	complete	Firmicutes		_		
Staphylococcus epidermidis ATCC 12228	complete	Firmicutes	No	+	No	Facultative
Staphylococcus epidermidis RP62A	complete	Firmicutes	No	+	No	Facultative
Staphylococcus haemolyticus JCSC1435	complete	Firmicutes	No	+	No	Facultative
Staphylococcus saprophyticus subsp. saprophyticus ATCC 15305	complete	Firmicutes	No	+	No	Aerobic
Staphylothermus marinus F1	complete	Crenarchaeota	No	_	No	Anaerobic
Stenotrophomonas maltophilia K279a	complete	Gammaproteobacteria		-		Aerobic
Stenotrophomonas maltophilia R551-3	complete	Gammaproteobacteria		-		Aerobic
Streptococcus agalactiae 18RS21	assembly	Firmicutes	No	+	No	Facultative
Streptococcus agalactiae 2603V/R	complete	Firmicutes	No	+	No	Facultative
Streptococcus agalactiae 515	assembly	Firmicutes	No	+	No	Facultative
Streptococcus agalactiae A909	complete	Firmicutes	No	+	No	Facultative
Streptococcus agalactiae CJB111	assembly	Firmicutes	No	+	No	Facultative

			phenotype categories			
name	*sequence status*	*taxonomic group*	*Endospores*	*Gram Stain*	*Motility*	*Oxygene Req.*
Streptococcus agalactiae COH1	assembly	Firmicutes	No	+	No	Facultative
Streptococcus agalactiae H36B	assembly	Firmicutes	No	+	No	Facultative
Streptococcus agalactiae NEM316	complete	Firmicutes	No	+	No	Facultative
Streptococcus equi subsp. equi 4047	complete	Firmicutes	No	+	No	Facultative
Streptococcus equi subsp. zooepidemicus	complete	Firmicutes	No	+	No	Facultative
Streptococcus equi subsp. zooepidemicus MGCS10565	complete	Firmicutes	No	+	No	Facultative
Streptococcus gordonii str. Challis substr. CH1	complete	Firmicutes	No	+	No	Facultative
Streptococcus mutans UA159	complete	Firmicutes	No	+	No	Facultative
Streptococcus pneumoniae ATCC 700669	complete	Firmicutes	No	+	No	Facultative
Streptococcus pneumoniae CDC0288-04	assembly	Firmicutes	No	+	No	Facultative
Streptococcus pneumoniae CDC1087-00	assembly	Firmicutes	No	+	No	Facultative
Streptococcus pneumoniae CDC1873-00	assembly	Firmicutes	No	+	No	Facultative
Streptococcus pneumoniae CDC3059-06	assembly	Firmicutes	No	+	No	Facultative
Streptococcus pneumoniae CGSP14	complete	Firmicutes	No	+	No	Facultative
Streptococcus pneumoniae D39	complete	Firmicutes	No	+	No	Facultative
Streptococcus pneumoniae G54	complete	Firmicutes	No	+	No	Facultative
Streptococcus pneumoniae Hungary19A-6	complete	Firmicutes	No	+	No	Facultative
Streptococcus pneumoniae MLV-016	assembly	Firmicutes	No	+	No	Facultative
Streptococcus pneumoniae R6	complete	Firmicutes	No	+	No	Facultative
Streptococcus pneumoniae SP11-BS70	assembly	Firmicutes	No	+	No	Facultative
Streptococcus pneumoniae SP14-BS69	assembly	Firmicutes	No	+	No	Facultative
Streptococcus pneumoniae SP18-BS74	assembly	Firmicutes	No	+	No	Facultative
Streptococcus pneumoniae SP19-BS75	assembly	Firmicutes	No	+	No	Facultative
Streptococcus pneumoniae SP195	assembly	Firmicutes	No	+	No	Facultative
Streptococcus pneumoniae SP23-BS72	assembly	Firmicutes	No	+	No	Facultative
Streptococcus pneumoniae SP3-BS71	assembly	Firmicutes	No	+	No	Facultative
Streptococcus pneumoniae SP6-BS73	assembly	Firmicutes	No	+	No	Facultative
Streptococcus pneumoniae SP9-BS68	assembly	Firmicutes	No	+	No	Facultative
Streptococcus pneumoniae TIGR4	assembly	Firmicutes	No	+	No	Facultative
Streptococcus pyogenes M1 GAS	complete	Firmicutes	No	+	No	Facultative
Streptococcus pyogenes MGAS10270	complete	Firmicutes	No	+	No	Facultative
Streptococcus pyogenes MGAS10394	complete	Firmicutes	No	+	No	Facultative
Streptococcus pyogenes MGAS10750	complete	Firmicutes	No	+	No	Facultative
Streptococcus pyogenes MGAS2096	complete	Firmicutes	No	+	No	Facultative
Streptococcus pyogenes MGAS315	complete	Firmicutes	No	+	No	Facultative
Streptococcus pyogenes MGAS5005	complete	Firmicutes	No	+	No	Facultative
Streptococcus pyogenes MGAS6180	complete	Firmicutes	No	+	No	Facultative
Streptococcus pyogenes MGAS8232	complete	Firmicutes	No	+	No	Facultative
Streptococcus pyogenes MGAS9429	complete	Firmicutes	No	+	No	Facultative
Streptococcus pyogenes NZ131	complete	Firmicutes	No	+	No	Facultative
Streptococcus pyogenes SSI-1	complete	Firmicutes	No	+	No	Facultative
Streptococcus pyogenes str. Manfredo	complete	Firmicutes	No	+	No	Facultative
Streptococcus sanguinis SK36	complete	Firmicutes	No	+	No	Facultative
Streptococcus suis 05ZYH33	complete	Firmicutes	No	+	No	Facultative
Streptococcus suis 98HAH33	complete	Firmicutes	No	+	No	Facultative
Streptococcus thermophilus CNRZ1066	complete	Firmicutes	No	+	No	Anaerobic
Streptococcus thermophilus LMD-9	complete	Firmicutes	No	+	No	Facultative
Streptococcus thermophilus LMG 18311	complete	Firmicutes	No	+	No	Anaerobic
Streptococcus uberis 0140J	complete	Firmicutes	No	+	No	Facultative
Streptomyces avermitilis MA-4680	complete	Actinobacteria	Yes	+	No	Aerobic
Streptomyces coelicolor A3(2)	complete	Actinobacteria	Yes	+	No	Aerobic
Streptomyces griseus subsp. griseus NBRC 13350	complete	Actinobacteria	Yes	+	No	Aerobic
Sulfolobus acidocaldarius DSM 639	complete	Crenarchaeota	No	_	No	Aerobic
Sulfolobus solfataricus P2	complete	Crenarchaeota	No	_	No	Aerobic
Sulfolobus tokodaii str. 7	complete	Crenarchaeota	No	_	No	Aerobic
Sulfurihydrogenibium sp. YO3AOP1	complete	Aquificae		-	Yes	Facultative
Sulfurimonas denitrificans DSM 1251	complete	Epsilonproteobacteria	No	-	No	Anaerobic
Sulfurovum sp. NBC37-1	complete	Epsilonproteobacteria		-	No	Facultative
Symbiobacterium thermophilum IAM 14863	complete	Firmicutes	No	+	No	Microaerophilic
Synechococcus elongatus PCC 6301	complete	Cyanobacteria		-	No	
Synechococcus elongatus PCC 7942	complete	Cyanobacteria		_		
Synechococcus sp. CC9311	complete	Cyanobacteria		_	Yes	
Synechococcus sp. CC9605	complete	Cyanobacteria	No	_	Yes	
Synechococcus sp. CC9902	complete	Cyanobacteria		_	Yes	
Synechococcus sp. JA-2-3B'a(2-13)	complete	Cyanobacteria		-		Facultative
Synechococcus sp. JA-3-3Ab	complete	Cyanobacteria		-		Facultative
Synechococcus sp. PCC 7002	complete	Cyanobacteria		_	No	
Synechococcus sp. RCC307	complete	Cyanobacteria		_	Yes	
Synechococcus sp. WH 7803	complete	Cyanobacteria		_	Yes	
Synechococcus sp. WH 8102	complete	Cyanobacteria		_	Yes	
Synechocystis sp. PCC 6803	complete	Cyanobacteria		_		
Syntrophobacter fumaroxidans MPOB	complete	Deltaproteobacteria	No	-	No	Anaerobic
Syntrophomonas wolfei subsp. wolfei str. Goettingen	complete	Other Bacteria	No	-	Yes	Anaerobic
Syntrophus aciditrophicus SB	complete	Deltaproteobacteria	No	-	No	Anaerobic
Thermoanaerobacter pseudethanolicus ATCC 33223	complete	Firmicutes	Yes	+		Anaerobic
Thermoanaerobacter sp. X514	complete	Firmicutes		+		Anaerobic
Thermoanaerobacter tengcongensis MB4	complete	Firmicutes		-	Yes	Anaerobic
Thermobifida fusca YX	complete	Actinobacteria	Yes	+	No	Aerobic
Thermococcus kodakarensis KOD1	complete	Euryarchaeota		_	Yes	Anaerobic
Thermococcus onnurineus NA1	complete	Euryarchaeota	No	_	Yes	Anaerobic
Thermodesulfovibrio yellowstonii DSM 11347	complete	Other Bacteria		-	Yes	
Thermofilum pendens Hrk 5	complete	Crenarchaeota	No	_	No	Anaerobic
Thermomicrobium roseum DSM 5159	complete	Chloroflexi	No	-	No	Aerobic
Thermoplasma acidophilum DSM 1728	complete	Euryarchaeota	No	_	Yes	Facultative
Thermoplasma volcanium GSS1	complete	Euryarchaeota	No	_	Yes	Facultative
Thermoproteus neutrophilus V24Sta	complete	Crenarchaeota	No	_	No	Anaerobic
Thermosinus carboxydivorans Nor1	assembly	Firmicutes	No	-	Yes	Anaerobic
Thermosipho africanus TCF52B	complete	Thermotogae		-		Anaerobic
Thermosipho melanesiensis BI429	complete	Thermotogae		-		Anaerobic
Thermotoga lettingae TMO	complete	Thermotogae	No	-	Yes	Anaerobic
Thermotoga maritima MSB8	complete	Thermotogae	No	-	Yes	Anaerobic
Thermotoga neapolitana DSM 4359	unfinished	Thermotogae	No	-		Microaerophilic
Thermotoga petrophila RKU-1	complete	Thermotogae	No	-	Yes	Anaerobic
Thermotoga sp. RQ2	complete	Thermotogae	No	-	Yes	Anaerobic
Thermotogales bacterium TBF 19.5.1	assembly	Thermotogae		-	Yes	

			phenotype categories			
name	*sequence status*	*taxonomic group*	*Endospores*	*Gram Stain*	*Motility*	*Oxygene Req.*
Thermus aquaticus Y51MC23	assembly	Deinococcus-Thermus		-	No	Aerobic
Thermus thermophilus HB27	complete	Deinococcus-Thermus		_		Aerobic
Thermus thermophilus HB8	complete	Deinococcus-Thermus		_		Aerobic
Thioalkalivibrio sp. HL-EbGR7	complete	Gammaproteobacteria	No	-	Yes	Aerobic
Thioalkalivibrio sp. K90mix	assembly	Gammaproteobacteria		-	Yes	
Thiobacillus denitrificans ATCC 25259	complete	Betaproteobacteria	No	-	Yes	Facultative
Thiomicrospira crunogena XCL-2	complete	Gammaproteobacteria	No	-	Yes	Microaerophilic
Treponema denticola ATCC 35405	complete	Spirochaetes	No	_	Yes	Anaerobic
Treponema pallidum subsp. pallidum SS14	complete	Spirochaetes	No	_	Yes	Anaerobic
Treponema pallidum subsp. pallidum str. Nichols	unfinished	Spirochaetes	No	-	Yes	Anaerobic
Trichodesmium erythraeum IMS101	complete	Cyanobacteria		_	Yes	Aerobic
Tropheryma whipplei str. Twist	complete	Actinobacteria	No	+	No	Aerobic
Tropheryma whipplei TW08/27	complete	Actinobacteria	No	+	No	Aerobic
uncultured Termite group 1 bacterium phylotype Rs-D17	complete	Other Bacteria		_		
unidentified eubacterium SCB49	assembly	Bacteroidetes/Chlorobi		_		
Ureaplasma parvum serovar 1 str. ATCC 27813	assembly	Firmicutes		_		Facultative
Ureaplasma parvum serovar 14 str. ATCC 33697	assembly	Firmicutes		_		Facultative
Ureaplasma parvum serovar 3 str. ATCC 27815	complete	Firmicutes		_		Facultative
Ureaplasma parvum serovar 3 str. ATCC 700970	complete	Firmicutes		_		Facultative
Ureaplasma parvum serovar 6 str. ATCC 27818	assembly	Firmicutes		_		Facultative
Ureaplasma urealyticum serovar 10 str. ATCC 33699	complete	Other Bacteria		+		Facultative
Ureaplasma urealyticum serovar 12 str. ATCC 33696	assembly	Other Bacteria		+		Facultative
Ureaplasma urealyticum serovar 13 str. ATCC 33698	assembly	Firmicutes		+		Facultative
Ureaplasma urealyticum serovar 2 str. ATCC 27814	assembly	Other Bacteria		+		Facultative
Ureaplasma urealyticum serovar 5 str. ATCC 27817	assembly	Firmicutes		+		Facultative
Ureaplasma urealyticum serovar 9 str. ATCC 33175	assembly	Other Bacteria		+		Facultative
Verminephrobacter eiseniae EF01-2	complete	Betaproteobacteria		-		
Vibrio campbellii AND4	assembly	Gammaproteobacteria		-	Yes	Facultative
Vibrio cholerae 1587	assembly	Gammaproteobacteria	No	-	Yes	Facultative
Vibrio cholerae 2740-80	assembly	Gammaproteobacteria		-	Yes	Facultative
Vibrio cholerae MAK 757	assembly	Gammaproteobacteria	No	-	Yes	Facultative
Vibrio cholerae MZO-3	assembly	Gammaproteobacteria		-	Yes	Facultative
Vibrio cholerae O1 biovar El Tor str. N16961	complete	Gammaproteobacteria		-	Yes	Facultative
Vibrio cholerae O395	complete	Gammaproteobacteria	No	-	Yes	Facultative
Vibrio cholerae V52	assembly	Gammaproteobacteria		-	Yes	Facultative
Vibrio fischeri ES114	complete	Gammaproteobacteria	No	-	Yes	Facultative
Vibrio fischeri MJ11	complete	Gammaproteobacteria	No	-	Yes	Facultative
Vibrio harveyi ATCC BAA-1116	unfinished	Gammaproteobacteria		-	Yes	Facultative
Vibrio parahaemolyticus RIMD 2210633	complete	Gammaproteobacteria		-	Yes	Facultative
Vibrio shilonii AK1	assembly	Gammaproteobacteria	No	-	Yes	Facultative
Vibrio splendidus LGP32	complete	Gammaproteobacteria	No	-	Yes	Facultative
Vibrio vulnificus CMCP6	complete	Gammaproteobacteria		-	Yes	Facultative
Vibrio vulnificus YJ016	complete	Gammaproteobacteria		-	Yes	Facultative
Vibrionales bacterium SWAT-3	assembly	Gammaproteobacteria		_	No	
Victivallis vadensis ATCC BAA-548	assembly	Other Bacteria		-	No	Anaerobic
Wigglesworthia glossinidia endosymbiont of Glossina brevipalpis	complete	Gammaproteobacteria		-		
Wolbachia endosymbiont of Culex quinquefasciatus Pel	complete	Alphaproteobacteria		-		Aerobic
Wolbachia endosymbiont of Drosophila ananassae	assembly	Alphaproteobacteria		-		
Wolbachia endosymbiont of Drosophila simulans	assembly	Alphaproteobacteria		-		
Wolbachia endosymbiont strain TRS of Brugia malayi	complete	Alphaproteobacteria	No	-	No	
Wolbachia sp. wRi	complete	Alphaproteobacteria		_		
Wolinella succinogenes DSM 1740	complete	Epsilonproteobacteria		-	Yes	Microaerophilic
Xanthobacter autotrophicus Py2	complete	Alphaproteobacteria		-	Yes	Facultative
Xanthomonas axonopodis pv. citri str. 306	complete	Gammaproteobacteria		-	Yes	Aerobic
Xanthomonas campestris pv. campestris str. 8004	complete	Gammaproteobacteria		-	Yes	Aerobic
Xanthomonas campestris pv. campestris str. ATCC 33913	complete	Gammaproteobacteria		-	Yes	Aerobic
Xanthomonas oryzae pv. oryzae KACC10331	complete	Gammaproteobacteria		-		Aerobic
Xanthomonas oryzae pv. oryzae MAFF 311018	complete	Gammaproteobacteria		-	Yes	Aerobic
Xanthomonas oryzae pv. oryzae PXO99A	complete	Gammaproteobacteria		-	Yes	Aerobic
Xylella fastidiosa 9a5c	complete	Gammaproteobacteria		-		Aerobic
Xylella fastidiosa Ann-1	assembly	Gammaproteobacteria		-		Aerobic
Xylella fastidiosa Dixon	assembly	Gammaproteobacteria		-		Aerobic
Xylella fastidiosa M12	complete	Gammaproteobacteria		-		Aerobic
Xylella fastidiosa M23	complete	Gammaproteobacteria		-		Aerobic
Xylella fastidiosa Temecula1	complete	Gammaproteobacteria		_		Aerobic
Yersinia enterocolitica subsp. enterocolitica 8081	complete	Gammaproteobacteria		-	Yes	Facultative
Yersinia pestis Angola	complete	Gammaproteobacteria		-		Facultative
Yersinia pestis Antiqua	complete	Gammaproteobacteria		-		Facultative
Yersinia pestis biovar Antiqua str. B42003004	assembly	Gammaproteobacteria		-		Facultative
Yersinia pestis biovar Antiqua str. E1979001	assembly	Gammaproteobacteria		-		Facultative
Yersinia pestis biovar Antiqua str. UG05-0454	assembly	Gammaproteobacteria		-		Facultative
Yersinia pestis biovar Mediaevalis str. K1973002	assembly	Gammaproteobacteria		-		Facultative
Yersinia pestis biovar Microtus str. 91001	complete	Gammaproteobacteria		-		Facultative
Yersinia pestis biovar Orientalis str. F1991016	assembly	Gammaproteobacteria		-		Facultative
Yersinia pestis biovar Orientalis str. MG05-1020	assembly	Gammaproteobacteria		-		Facultative
Yersinia pestis CO92	complete	Gammaproteobacteria		-		Facultative
Yersinia pestis KIM	complete	Gammaproteobacteria		-		Facultative
Yersinia pestis Nepal516	assembly	Gammaproteobacteria		-		Facultative
Yersinia pestis Pestoides F	complete	Gammaproteobacteria		-		Facultative
Yersinia pseudotuberculosis IP 31758	complete	Gammaproteobacteria		-	Yes	Facultative
Yersinia pseudotuberculosis IP 32953	complete	Gammaproteobacteria		-	Yes	Facultative
Yersinia pseudotuberculosis PB1/+	complete	Gammaproteobacteria		-	Yes	Facultative
Yersinia pseudotuberculosis YPIII	complete	Gammaproteobacteria		-	Yes	Facultative
Zymomonas mobilis subsp. mobilis ZM4	unfinished	Alphaproteobacteria		_		

Total: 1032
complete/assembly/unfinished: 795/226/11

Organisms used for test set

			phenotype categories			
name	*sequence status*	*taxonomic group*	*Endospores*	*Gram Stain*	*Motility*	*Oxygene Req.*
Acetobacter pasteurianus IFO 3283-01	complete	Alphaproteobacteria		-		Aerobic
Acetobacter pasteurianus IFO 3283-01-42C	unfinished	Alphaproteobacteria		-		Aerobic
Acetobacter pasteurianus IFO 3283-03	unfinished	Alphaproteobacteria		-		Aerobic
Acetobacter pasteurianus IFO 3283-07	unfinished	Alphaproteobacteria		-		Aerobic

			phenotype categories			
name	*sequence status*	*taxonomic group*	*Endospores*	*Gram Stain*	*Motility*	*Oxygene Req.*
Acetobacter pasteurianus IFO 3283-12	unfinished	Alphaproteobacteria				
Acetobacter pasteurianus IFO 3283-22	unfinished	Alphaproteobacteria		-		Aerobic
Acetobacter pasteurianus IFO 3283-26	unfinished	Alphaproteobacteria		-		Aerobic
Acetobacter pasteurianus IFO 3283-32	unfinished	Alphaproteobacteria		-		Aerobic
Acidaminococcus sp. D21	assembly	Firmicutes				
Acidimicrobium ferrooxidans DSM 10331	complete	Actinobacteria	No	+		Anaerobic
Acidobacterium capsulatum ATCC 51196	complete	Acidobacteria	No	-	No	Aerobic
Acidovorax delafieldii 2AN	assembly	Betaproteobacteria	No	-	Yes	Aerobic
Acinetobacter radioresistens SK82	assembly	Gammaproteobacteria				
Acinetobacter sp. ATCC 27244	assembly	Gammaproteobacteria				
Actinobacillus minor 202	assembly	Gammaproteobacteria		-		
Actinomyces odontolyticus ATCC 17982	assembly	Actinobacteria	No	+	No	Facultative
Actinomyces urogenitalis DSM 15434	assembly	Actinobacteria				
Actinosynnema mirum DSM 43827	complete	Actinobacteria		+		
Aggregatibacter aphrophilus NJ8700	complete	Gammaproteobacteria				
Alistipes putredinis DSM 17216	assembly	Bacteroidetes/Chlorobi	No	-	No	Anaerobic
Anaerococcus prevotii DSM 20548	complete	Firmicutes	No	+	No	Anaerobic
Anaerococcus tetradius ATCC 35098	assembly	Firmicutes				
Anaerofustis stercorihominis DSM 17244	assembly	Firmicutes	No	+		Anaerobic
Anaerostipes caccae DSM 14662	assembly	Firmicutes	No			Anaerobic
Anaerotruncus colihominis DSM 17241	assembly	Firmicutes		+	No	Anaerobic
Asticcacaulis excentricus CB 48	assembly	Alphaproteobacteria	No	-	Yes	Aerobic
Azotobacter vinelandii DJ	complete	Gammaproteobacteria		-	Yes	Aerobic
Bacillus anthracis str. A0248	complete	Firmicutes	Yes	+	Yes	Facultative
Bacillus anthracis str. CDC 684	complete	Firmicutes				
Bacillus cereus 03BB102	complete	Firmicutes	Yes	+	No	Aerobic
Bacillus cereus 172560W	assembly	Firmicutes	Yes	+	Yes	Aerobic
Bacillus cereus 95/8201	assembly	Firmicutes	Yes	+	Yes	Aerobic
Bacillus cereus AH1271	assembly	Firmicutes	Yes	+	Yes	Aerobic
Bacillus cereus AH1272	assembly	Firmicutes	Yes	+	Yes	Aerobic
Bacillus cereus AH1273	assembly	Firmicutes	Yes	+	Yes	Aerobic
Bacillus cereus AH603	assembly	Firmicutes	Yes	+	Yes	Aerobic
Bacillus cereus AH621	assembly	Firmicutes	Yes	+	Yes	Aerobic
Bacillus cereus AH676	assembly	Firmicutes	Yes	+	Yes	Aerobic
Bacillus cereus ATCC 10876	assembly	Firmicutes	Yes	+	Yes	Aerobic
Bacillus cereus ATCC 4342	assembly	Firmicutes	Yes	+	Yes	Aerobic
Bacillus cereus BDRD-Cer4	assembly	Firmicutes	Yes	+	Yes	Aerobic
Bacillus cereus BDRD-ST196	assembly	Firmicutes	Yes	+	Yes	Aerobic
Bacillus cereus BDRD-ST24	assembly	Firmicutes	Yes	+	Yes	Aerobic
Bacillus cereus BDRD-ST26	assembly	Firmicutes	Yes	+	Yes	Aerobic
Bacillus cereus F65185	assembly	Firmicutes	Yes	+	Yes	Aerobic
Bacillus cereus H3081.97	assembly	Firmicutes	Yes	+	Yes	Aerobic
Bacillus cereus m1293	assembly	Firmicutes	Yes	+	Yes	Aerobic
Bacillus cereus m1550	assembly	Firmicutes	Yes	+	Yes	Aerobic
Bacillus cereus MM3	assembly	Firmicutes	Yes	+	Yes	Aerobic
Bacillus cereus R309803	assembly	Firmicutes	Yes	+	Yes	Aerobic
Bacillus cereus Rock3-28	assembly	Firmicutes	Yes	+	Yes	Aerobic
Bacillus cereus Rock3-29	assembly	Firmicutes	Yes	+	Yes	Aerobic
Bacillus cereus Rock3-44	assembly	Firmicutes	Yes	+	Yes	Aerobic
Bacillus cereus Rock4-18	assembly	Firmicutes	Yes	+	Yes	Aerobic
Bacillus mycoides DSM 2048	assembly	Firmicutes	Yes	+	No	
Bacillus mycoides Rock1-4	assembly	Firmicutes	Yes	+	No	Aerobic
Bacillus mycoides Rock3-17	assembly	Firmicutes	Yes	+	No	Aerobic
Bacillus pseudomycoides DSM 12442	assembly	Firmicutes	Yes	+	No	Aerobic
Bacillus sp. NRRL B-14911	assembly	Firmicutes	Yes	+		
Bacillus thuringiensis IBL 200	assembly	Firmicutes	Yes	+	Yes	Aerobic
Bacillus thuringiensis IBL 4222	assembly	Firmicutes	Yes	+	Yes	Aerobic
Bacillus thuringiensis serovar andalousiensis BGSC 4AW1	assembly	Firmicutes	Yes	+	Yes	Aerobic
Bacillus thuringiensis serovar huazhongensis BGSC 4BD1	assembly	Firmicutes	Yes	+	Yes	Aerobic
Bacillus thuringiensis serovar kurstaki str. T03a001	assembly	Firmicutes	Yes	+	Yes	Aerobic
Bacillus thuringiensis serovar pakistani str. T13001	assembly	Firmicutes	Yes	+	Yes	Aerobic
Bacillus thuringiensis serovar pondicheriensis BGSC 4BA1	assembly	Firmicutes	Yes	+	Yes	Aerobic
Bacillus thuringiensis serovar pulsiensis BGSC 4CC1	assembly	Firmicutes	Yes	+	Yes	Aerobic
Bacillus thuringiensis serovar sotto str. T04001	assembly	Firmicutes	Yes	+	Yes	Aerobic
Bacillus thuringiensis serovar thuringiensis str. T01001	assembly	Firmicutes	Yes	+	Yes	Aerobic
Bacillus thuringiensis serovar tochigiensis BGSC 4Y1	assembly	Firmicutes	Yes	+	Yes	Aerobic
Bacteroides capillosus ATCC 29799	assembly	Bacteroidetes/Chlorobi		-		Anaerobic
Bacteroides coprocola DSM 17136	assembly	Bacteroidetes/Chlorobi	No	-		Anaerobic
Bacteroides dorei DSM 17855	assembly	Bacteroidetes/Chlorobi	No	-	No	Anaerobic
Bacteroides eggerthii DSM 20697	assembly	Bacteroidetes/Chlorobi	No	-	No	Anaerobic
Bacteroides intestinalis DSM 17393	assembly	Bacteroidetes/Chlorobi	No	-		Anaerobic
Bacteroides ovatus ATCC 8483	assembly	Bacteroidetes/Chlorobi	No	-		Anaerobic
Bacteroides pectinophilus ATCC 43243	assembly	Bacteroidetes/Chlorobi	No	-	No	Anaerobic
Bacteroides plebeius DSM 17135	assembly	Bacteroidetes/Chlorobi	No	-	No	Anaerobic
Bacteroides stercoris ATCC 43183	assembly	Bacteroidetes/Chlorobi	No	-		Anaerobic
Bacteroides uniformis ATCC 8492	assembly	Bacteroidetes/Chlorobi	No	-		Anaerobic
Bartonella grahamii as4aup	complete	Alphaproteobacteria		-		Aerobic
Bermanella marisrubri	assembly	Gammaproteobacteria	No	-	Yes	Aerobic
Beutenbergia cavernae DSM 12333	complete	Actinobacteria	No	+	No	Aerobic
Bifidobacterium adolescentis L2-32	assembly	Actinobacteria	No	+	No	Anaerobic
Bifidobacterium angulatum DSM 20098	assembly	Actinobacteria	No	+	No	Anaerobic
Bifidobacterium animalis subsp. lactis Bl-04	complete	Actinobacteria	No	+	No	Anaerobic
Bifidobacterium animalis subsp. lactis DSM 10140	complete	Actinobacteria	No	+	No	Anaerobic
Bifidobacterium breve DSM 20213	assembly	Actinobacteria	No	+	No	Anaerobic
Bifidobacterium dentium ATCC 27678	assembly	Actinobacteria	Yes	+		Anaerobic
Bifidobacterium longum subsp. infantis ATCC 55813	assembly	Actinobacteria				
Blastopirellula marina DSM 3645	assembly	Planctomycetes				
Blautia hydrogenotrophica DSM 10507	assembly	Firmicutes	No	+		Anaerobic
Borrelia burgdorferi 118a	assembly	Spirochaetes	No		Yes	Microaerophilic
Borrelia burgdorferi 29805	assembly	Spirochaetes			Yes	Microaerophilic
Borrelia burgdorferi 64b	assembly	Spirochaetes			Yes	Microaerophilic
Borrelia burgdorferi 72a	assembly	Spirochaetes	No		Yes	Microaerophilic
Borrelia burgdorferi 94a	assembly	Spirochaetes	No		Yes	Microaerophilic
Borrelia burgdorferi Bol26	assembly	Spirochaetes	No		Yes	Microaerophilic
Borrelia burgdorferi WI91-23	assembly	Spirochaetes			Yes	Microaerophilic
Borrelia garinii Far04	assembly	Spirochaetes			Yes	Microaerophilic

			phenotype categories			
name	*sequence status*	*taxonomic group*	*Endospores*	*Gram Stain*	*Motility*	*Oxygene Req.*
Borrelia garinii PBr	assembly	Spirochaetes			Yes	Microaerophilic
Borrelia sp. SV1	assembly	Spirochaetes			Yes	Microaerophilic
Borrelia spielmanii A14S	assembly	Spirochaetes			Yes	Microaerophilic
Brachybacterium faecium DSM 4810	complete	Actinobacteria		+	No	Aerobic
Brachyspira murdochii DSM 12563	assembly	Spirochaetes	No	-	Yes	Facultative
Brevibacillus brevis NBRC 100599	complete	Firmicutes		+	Yes	Aerobic
Brucella ceti str. Cudo	assembly	Alphaproteobacteria		-		Aerobic
Brucella melitensis ATCC 23457	complete	Alphaproteobacteria		-		Facultative
Brucella microti CCM 4915	complete	Alphaproteobacteria				
Bryantella formatexigens DSM 14469	assembly	Firmicutes	No	+	No	Anaerobic
Burkholderia glumae BGR1	complete	Betaproteobacteria		-	No	Aerobic
Burkholderia mallei GB8 horse 4	assembly	Betaproteobacteria		-	No	
Burkholderia mallei PRL-20	assembly	Betaproteobacteria		-	No	Aerobic
Burkholderia pseudomallei MSHR346	assembly	Betaproteobacteria		-	Yes	Aerobic
Campylobacter showae RM3277	assembly	Epsilonproteobacteria		-		
Candidatus Accumulibacter phosphatis clade IIA str. UW-1	complete	Betaproteobacteria		-		
Candidatus Hamiltonella defensa 5AT (Acyrthosiphon pisum)	complete	Gammaproteobacteria				
Candidatus Liberibacter asiaticus str. psy62	complete	Alphaproteobacteria				
Candidatus Pelagibacter ubique HTCC1002	assembly	Alphaproteobacteria		-		Aerobic
Candidatus Sulcia muelleri SMDSEM	complete	Bacteroidetes/Chlorobi				
Capnocytophaga gingivalis ATCC 33624	assembly	Bacteroidetes/Chlorobi				
Capnocytophaga ochracea DSM 7271	complete	Bacteroidetes/Chlorobi	No	-	Yes	Facultative
Catenulispora acidiphila DSM 44928	complete	Actinobacteria	Yes	+		Aerobic
Cellulomonas flavigena DSM 20109	assembly	Actinobacteria		+	Yes	Facultative
Chitinophaga pinensis DSM 2588	complete	Bacteroidetes/Chlorobi	No	-	Yes	Aerobic
Chlamydia trachomatis B/Jali20/OT	unfinished	Chlamydiae/Verrucomicrobia				
Chlamydia trachomatis B/Jali20/OT	complete	Chlamydiae/Verrucomicrobia		-		
Chryseobacterium gleum ATCC 35910	assembly	Bacteroidetes/Chlorobi				
Citrobacter youngae ATCC 29220	assembly	Gammaproteobacteria		-		Anaerobic
Clostridium asparagiforme DSM 15981	assembly	Firmicutes	Yes	+		Anaerobic
Clostridium bartlettii DSM 16795	assembly	Firmicutes	Yes	+		Anaerobic
Clostridium bolteae ATCC BAA-613	assembly	Firmicutes	Yes	+	Yes	Anaerobic
Clostridium botulinum A2 str. Kyoto	complete	Firmicutes	Yes	+	Yes	Anaerobic
Clostridium botulinum Ba4 str. 657	complete	Firmicutes	Yes	_	Yes	Anaerobic
Clostridium botulinum D str. 1873	assembly	Firmicutes	Yes	+	Yes	Anaerobic
Clostridium butyricum E4 str. BoNT E BL5262	assembly	Firmicutes				
Clostridium cellulovorans 743B	assembly	Firmicutes	Yes	-	No	Anaerobic
Clostridium cellulovorans 743B	unfinished	Firmicutes				
Clostridium hiranonis DSM 13275	assembly	Firmicutes	Yes	+	No	Anaerobic
Clostridium hylemonae DSM 15053	assembly	Firmicutes		+	No	Anaerobic
Clostridium kluyveri NBRC 12016	complete	Firmicutes	Yes	+	Yes	Anaerobic
Clostridium leptum DSM 753	assembly	Firmicutes	Yes	+	Yes	Anaerobic
Clostridium nexile DSM 1787	assembly	Firmicutes	Yes	+		Anaerobic
Clostridium papyrosolvens DSM 2782	assembly	Firmicutes	Yes	-		
Clostridium ramosum DSM 1402	assembly	Firmicutes	Yes	+		Anaerobic
Clostridium scindens ATCC 35704	assembly	Firmicutes	Yes	+	Yes	Anaerobic
Clostridium sp. L2-50	assembly	Firmicutes	Yes	+		Anaerobic
Clostridium sp. M62/1	assembly	Firmicutes		+		Anaerobic
Clostridium sp. SS2/1	assembly	Firmicutes		+		Anaerobic
Clostridium spiroforme DSM 1552	assembly	Firmicutes	Yes	+	No	Anaerobic
Collinsella stercoris DSM 13279	assembly	Actinobacteria	No	+	No	Anaerobic
Congregibacter litoralis KT71	assembly	Gammaproteobacteria				
Coprococcus comes ATCC 27758	assembly	Firmicutes	No	+	No	Anaerobic
Coprococcus eutactus ATCC 27759	assembly	Firmicutes	No	+	No	Anaerobic
Corynebacterium accolens ATCC 49725	assembly	Actinobacteria				
Corynebacterium aurimucosum ATCC 700975	complete	Actinobacteria	No	+	No	Facultative
Corynebacterium aurimucosum ATCC 700975	assembly	Actinobacteria	N	+	N	Facultative
Corynebacterium glucuronolyticum ATCC 51866	assembly	Actinobacteria				
Corynebacterium glucuronolyticum ATCC 51867	assembly	Actinobacteria				
Corynebacterium kroppenstedtii DSM 44385	complete	Actinobacteria	No	+	-	Facultative
Corynebacterium lipophiloflavum DSM 44291	assembly	Actinobacteria				
Corynebacterium matruchotii ATCC 14266	assembly	Actinobacteria		+		
Corynebacterium matruchotii ATCC 33806	assembly	Actinobacteria				
Corynebacterium pseudogenitalium ATCC 33035	assembly	Actinobacteria				
Corynebacterium striatum ATCC 6940	assembly	Actinobacteria				
Corynebacterium tuberculostearicum SK141	assembly	Actinobacteria		+	Non-motile	
Croceibacter atlanticus HTCC2559	assembly	Bacteroidetes/Chlorobi		-	No	Aerobic
Cryptobacterium curtum DSM 15641	complete	Actinobacteria	No	+	No	Anaerobic
Deinococcus deserti VCD115	complete	Deinococcus-Thermus	No	-	No	Aerobic
Denitrovibrio acetiphilus DSM 12809	assembly	Other Bacteria	No	-	Yes	Anaerobic
Desulfomicrobium baculatum DSM 4028	complete	Deltaproteobacteria		-		Anaerobic
Desulfovibrio magneticus RS-1	complete	Deltaproteobacteria				
Desulfovibrio piger ATCC 29098	assembly	Deltaproteobacteria	No	-	Yes	Anaerobic
Desulfovibrio vulgaris RCH1	assembly	Deltaproteobacteria				
Dethiosulfovibrio peptidovorans DSM 11002	assembly	Other Bacteria	No	-	Yes	Aerobic
Dickeya dadantii Ech703	complete	Gammaproteobacteria	No	-	Yes	Facultative
Dickeya zeae Ech1591	complete	Gammaproteobacteria				
Dokdonia donghaensis MED134	assembly	Bacteroidetes/Chlorobi				
Dorea formicigenerans ATCC 27755	assembly	Firmicutes	No	+		Anaerobic
Dorea longicatena DSM 13814	assembly	Firmicutes	No	+		Anaerobic
Dyadobacter fermentans DSM 18053	complete	Bacteroidetes/Chlorobi	No	-	No	Aerobic
Edwardsiella ictaluri 93-146	complete	Gammaproteobacteria	No	-	Yes	Facultative
Eikenella corrodens ATCC 23834	assembly	Betaproteobacteria	No	-	Yes	Anaerobic
Enterobacter cancerogenus ATCC 35316	assembly	Gammaproteobacteria		-		Facultative
Enterococcus faecalis ATCC 29200	assembly	Firmicutes				
Enterococcus faecalis HH22	assembly	Firmicutes				
Enterococcus faecalis TUSoD Ef11	assembly	Firmicutes				
Enterococcus faecalis TX0104	assembly	Firmicutes		+		Facultative
Enterococcus faecalis TX1322	assembly	Firmicutes				
Enterococcus faecium TX1330	assembly	Firmicutes				
Erythrobacter sp. NAP1	assembly	Alphaproteobacteria	No	-	Yes	Aerobic
Escherichia albertii TW07627	assembly	Gammaproteobacteria		-	No	Facultative
Escherichia coli 53638	assembly	Gammaproteobacteria	No	-	Yes	Facultative
Escherichia coli 83972	assembly	Gammaproteobacteria				
Escherichia coli B str. REL606	complete	Gammaproteobacteria	No	-	Yes	Facultative
Escherichia coli B171	assembly	Gammaproteobacteria		-	Yes	
Escherichia coli BL21	complete	Gammaproteobacteria	No	-	Yes	Facultative

			phenotype categories			
name	*sequence status*	*taxonomic group*	*Endospores*	*Gram Stain*	*Motility*	*Oxygene Req.*
Escherichia coli BW2952	complete	Gammaproteobacteria	No	-	Yes	Facultative
Escherichia coli O157:H7 str. TW14359	complete	Gammaproteobacteria	No	-	Yes	Facultative
Escherichia coli O157:H7 str. TW14588	assembly	Gammaproteobacteria		-	Yes	Facultative
Eubacterium biforme DSM 3989	assembly	Firmicutes	No	+		Anaerobic
Eubacterium dolichum DSM 3991	assembly	Firmicutes		+		Anaerobic
Eubacterium eligens ATCC 27750	complete	Firmicutes		-		Anaerobic
Eubacterium rectale ATCC 33656	complete	Firmicutes	No	+		Anaerobic
Eubacterium siraeum DSM 15702	assembly	Firmicutes		+		Anaerobic
Eubacterium ventriosum ATCC 27560	assembly	Firmicutes		+		Anaerobic
Faecalibacterium prausnitzii A2-165	assembly	Firmicutes	No	-	No	Anaerobic
Faecalibacterium prausnitzii M21/2	assembly	Firmicutes	No	-	No	Anaerobic
Fibrobacter succinogenes subsp. succinogenes S85	unfinished	Other Bacteria	No	-	No	Anaerobic
Fibrobacter succinogenes subsp. succinogenes S85	assembly	Other Bacteria	No	-	No	Anaerobic
Finegoldia magna ATCC 53516	assembly	Firmicutes				
Flavobacteria bacterium BBFL7	assembly	Bacteroidetes/Chlorobi				
Flavobacteriaceae bacterium 3519-10	complete	Bacteroidetes/Chlorobi				
Flavobacteriales bacterium HTCC2170	assembly	Bacteroidetes/Chlorobi				
Francisella novicida FTE	unfinished	Gammaproteobacteria	No	-	No	Aerobic
Francisella novicida FTE	assembly	Gammaproteobacteria	No	-	No	Aerobic
Fulvimarina pelagi HTCC2506	assembly	Alphaproteobacteria		-		Aerobic
Gemmatimonas aurantiaca T-27	complete	Other Bacteria	No	-	Yes	Aerobic
Geobacillus sp. Y4.1MC1	assembly	Firmicutes		+		Aerobic
Geobacillus sp. Y412MC52	assembly	Firmicutes	Yes	+	Yes	Facultative
Geodermatophilus obscurus DSM 43160	assembly	Actinobacteria		-	No	Aerobic
Gordonia bronchialis DSM 43247	assembly	Actinobacteria		+	No	Aerobic
Haliangium ochraceum DSM 14365	assembly	Deltaproteobacteria		-	Yes	Aerobic
Halogeometricum borinquense DSM 11551	assembly	Euryarchaeota	No	-	Yes	Aerobic
Halomicrobium mukohataei DSM 12286	complete	Euryarchaeota			Yes	Facultative
Halorhabdus utahensis DSM 12940	complete	Euryarchaeota	No	-	Yes	Aerobic
Helicobacter pylori B38	complete	Epsilonproteobacteria		-	Yes	Aerobic
Hirschia baltica ATCC 49814	complete	Alphaproteobacteria	No		Yes	Aerobic
Hoeflea phototrophica DFL-43	assembly	Alphaproteobacteria		-	Yes	Microaerophilic
Holdemania filiformis DSM 12042	assembly	Firmicutes	No	+		Anaerobic
Hyphomicrobium denitrificans ATCC 51888	assembly	Alphaproteobacteria	No	-	Yes	Aerobic
Idiomarina baltica OS145	assembly	Gammaproteobacteria		-	Yes	Aerobic
Janibacter sp. HTCC2649	assembly	Actinobacteria		+		
Jonesia denitrificans DSM 20603	complete	Actinobacteria	No	+	Yes	Facultative
Kangiella koreensis DSM 16069	complete	Gammaproteobacteria	No	-	No	Facultative
Kordia algicida OT-1	assembly	Bacteroidetes/Chlorobi		-	No	Aerobic
Kribbella flavida DSM 17836	assembly	Actinobacteria	Yes	+		Aerobic
Kytococcus sedentarius DSM 20547	complete	Actinobacteria	No	+	No	Aerobic
Lactobacillus acidophilus ATCC 4796	assembly	Firmicutes				
Lactobacillus brevis subsp. gravesensis ATCC 27305	assembly	Firmicutes				
Lactobacillus buchneri ATCC 11577	assembly	Firmicutes				
Lactobacillus fermentum ATCC 14931	assembly	Firmicutes				
Lactobacillus gasseri JV-V03	assembly	Firmicutes				
Lactobacillus hilgardii ATCC 8290	assembly	Firmicutes				
Lactobacillus jensenii 269-3	assembly	Firmicutes				
Lactobacillus jensenii JV-V16	assembly	Firmicutes				
Lactobacillus johnsonii ATCC 33200	assembly	Firmicutes				
Lactobacillus paracasei subsp. paracasei ATCC 25302	assembly	Firmicutes				
Lactobacillus plantarum JDM1	complete	Firmicutes				
Lactobacillus plantarum subsp. plantarum ATCC 14917	assembly	Firmicutes				
Lactobacillus reuteri CF48-3A	assembly	Firmicutes				
Lactobacillus reuteri MM2-3	assembly	Firmicutes				
Lactobacillus reuteri MM4-1A	assembly	Firmicutes				
Lactobacillus rhamnosus GG	unfinished	Firmicutes				
Lactobacillus rhamnosus GG	complete	Firmicutes		+		Facultative
Lactobacillus rhamnosus HN001	assembly	Firmicutes		+		Facultative
Lactobacillus rhamnosus Lc 705	complete	Firmicutes		+		Facultative
Lactobacillus rhamnosus LMS2-1	assembly	Firmicutes				
Lactobacillus ruminis ATCC 25644	assembly	Firmicutes				
Lactobacillus salivarius ATCC 11741	assembly	Firmicutes				
Lactobacillus ultunensis DSM 16047	assembly	Firmicutes				
Lactobacillus vaginalis ATCC 49540	assembly	Firmicutes				
Laribacter hongkongensis HLHK9	complete	Betaproteobacteria	No	-	Yes	Anaerobic
Leeuwenhoekiella blandensis MED217	assembly	Bacteroidetes/Chlorobi		-		
Leptotrichia buccalis DSM 1135	complete	Fusobacteria		-	No	Anaerobic
Leuconostoc mesenteroides subsp. cremoris ATCC 19254	assembly	Firmicutes				
Listeria grayi DSM 20601	assembly	Firmicutes				
Listeria monocytogenes Clip80459	complete	Firmicutes				
Loktanella vestfoldensis SKA53	assembly	Alphaproteobacteria		-		Aerobic
Macrococcus caseolyticus JCSC5402	complete	Firmicutes	No	+	No	Facultative
Marinomonas sp. MED121	assembly	Gammaproteobacteria		-	Yes	Aerobic
Mariprofundus ferrooxydans PV-1	assembly	Other Bacteria				
Meiothermus ruber DSM 1279	assembly	Deinococcus-Thermus			No	Aerobic
Meiothermus silvanus DSM 9946	assembly	Deinococcus-Thermus			No	Aerobic
Methanobrevibacter smithii DSM 2375	assembly	Euryarchaeota		+	No	Anaerobic
Methanocaldococcus fervens AG86	complete	Euryarchaeota		-		Anaerobic
Methylobacterium extorquens AM1	complete	Alphaproteobacteria		-	Yes	Facultative
Methylobacterium extorquens AM1	unfinished	Alphaproteobacteria				
Methylobacterium extorquens DM4	complete	Alphaproteobacteria		-	Yes	Aerobic
Methylotenera mobilis JLW8	complete	Betaproteobacteria	No	-	Yes	Aerobic
Methylovorus sp. SIP3-4	complete	Betaproteobacteria	No	-	Yes	Aerobic
Mobiluncus mulieris ATCC 35243	assembly	Actinobacteria				
Mycobacterium bovis BCG str. Tokyo 172	complete	Actinobacteria	No	+	No	Aerobic
Mycobacterium tuberculosis KZN 1435	complete	Actinobacteria	No	+	Yes	Aerobic
Mycoplasma conjunctivae	complete	Other Bacteria	No		No	Microaerophilic
Mycoplasma mycoides subsp. capri str. GM12	complete	Firmicutes	No	-	No	Anaerobic
Mycoplasma mycoides subsp. capri str. GM12	complete	Firmicutes				
Neisseria flavescens SK114	assembly	Betaproteobacteria				
Neisseria sicca ATCC 29256	assembly	Betaproteobacteria	No	-	No	Aerobic
Neorickettsia risticii str. Illinois	complete	Alphaproteobacteria				
Neptuniibacter caesariensis	assembly	Gammaproteobacteria		-	Yes	Aerobic
Nitrobacter sp. Nb-311A	assembly	Alphaproteobacteria		-		Facultative
Nitrococcus mobilis Nb-231	assembly	Gammaproteobacteria			Yes	Aerobic
Nitrosomonas sp. AL212	assembly	Betaproteobacteria	No	-	Yes	

			phenotype categories			
name	*sequence status*	*taxonomic group*	*Endospores*	*Gram Stain*	*Motility*	*Oxygene Req.*
Nocardiopsis dassonvillei subsp. dassonvillei DSM 43111	assembly	Actinobacteria				
Oceanicaulis alexandrii HTCC2633	assembly	Alphaproteobacteria		-	Yes	Aerobic
Oceanicola batsensis HTCC2597	assembly	Alphaproteobacteria		-	No	Aerobic
Oceanicola granulosus HTCC2516	assembly	Alphaproteobacteria		-	No	Aerobic
Oribacterium sinus F0268	assembly	Firmicutes				
Parabacteroides johnsonii DSM 18315	assembly	Bacteroidetes/Chlorobi	No	-		Anaerobic
Parabacteroides merdae ATCC 43184	assembly	Bacteroidetes/Chlorobi	No	-		Anaerobic
Parvimonas micra ATCC 33270	assembly	Firmicutes		+		Anaerobic
Parvularcula bermudensis HTCC2503	assembly	Alphaproteobacteria	No	-	Yes	Aerobic
Pectobacterium carotovorum subsp. carotovorum PC1	complete	Gammaproteobacteria				
Pectobacterium wasabiae WPP163	assembly	Gammaproteobacteria				
Pedobacter heparinus DSM 2366	complete	Bacteroidetes/Chlorobi	No	-	Yes	Aerobic
Phaeobacter gallaeciensis 2.10	assembly	Alphaproteobacteria				
Phaeobacter gallaeciensis BS107	assembly	Alphaproteobacteria		-		
Photobacterium angustum S14	assembly	Gammaproteobacteria		-	Yes	Facultative
Photobacterium profundum 3TCK	assembly	Gammaproteobacteria		-	Yes	Facultative
Photobacterium sp. SKA34	assembly	Gammaproteobacteria		-		Facultative
Photorhabdus asymbiotica	complete	Gammaproteobacteria	No	-	Yes	Facultative
Planctomyces limnophilus DSM 3776	assembly	Planctomycetes		-	Yes	Aerobic
Polaribacter irgensii 23-P	assembly	Bacteroidetes/Chlorobi		-	Yes	Aerobic
Porphyromonas endodontalis ATCC 35406	assembly	Bacteroidetes/Chlorobi				
Porphyromonas uenonis 60-3	assembly	Bacteroidetes/Chlorobi				
Prevotella copri DSM 18205	assembly	Bacteroidetes/Chlorobi	No	-	No	Anaerobic
Prevotella melaninogenica ATCC 25845	assembly	Bacteroidetes/Chlorobi				
Proteus mirabilis ATCC 29906	assembly	Gammaproteobacteria				
Proteus penneri ATCC 35198	assembly	Gammaproteobacteria	No	-	Yes	Anaerobic
Providencia alcalifaciens DSM 30120	assembly	Gammaproteobacteria		-	Yes	Facultative
Providencia rustigianii DSM 4541	assembly	Gammaproteobacteria	No	-	Yes	Facultative
Providencia stuartii ATCC 25827	assembly	Gammaproteobacteria	No	-	Yes	Facultative
Pseudoalteromonas tunicata D2	assembly	Gammaproteobacteria		-	Yes	Facultative
Pseudomonas fluorescens SBW25	complete	Gammaproteobacteria		-	Yes	Aerobic
Psychroflexus torquis ATCC 700755	assembly	Bacteroidetes/Chlorobi			Yes	Aerobic
Psychromonas sp. CNPT3	assembly	Gammaproteobacteria		-	Yes	Facultative
Ralstonia solanacearum IPO1609	unfinished	Betaproteobacteria				Aerobic
Reinekea blandensis MED297	assembly	Gammaproteobacteria				
Rhizobium sp. NGR234	complete	Alphaproteobacteria		-	Yes	Aerobic
Rhodobacterales bacterium HTCC2654	assembly	Alphaproteobacteria				
Rhodococcus erythropolis PR4	complete	Actinobacteria		+		
Rhodococcus erythropolis SK121	assembly	Actinobacteria				
Rhodococcus opacus B4	complete	Actinobacteria				
Rhodococcus opacus B4	complete	Actinobacteria		+	No	Aerobic
Rhodothermus marinus DSM 4252	assembly	Bacteroidetes/Chlorobi		-		Aerobic
Rickettsia africae ESF-5	complete	Alphaproteobacteria		-		Aerobic
Rickettsia peacockii str. Rustic	complete	Alphaproteobacteria		-		Aerobic
Robiginitalea biformata HTCC2501	complete	Bacteroidetes/Chlorobi		-		Aerobic
Roseobacter litoralis Och 149	assembly	Alphaproteobacteria				
Roseobacter sp. MED193	assembly	Alphaproteobacteria		-	Yes	Aerobic
Roseovarius nubinhibens ISM	assembly	Alphaproteobacteria		-	Yes	Aerobic
Roseovarius sp. 217	assembly	Alphaproteobacteria		-		Aerobic
Roseovarius sp. HTCC2601	assembly	Alphaproteobacteria		-		Aerobic
Ruminococcus gnavus ATCC 29149	assembly	Firmicutes	No	+		Anaerobic
Ruminococcus lactaris ATCC 29176	assembly	Firmicutes	No	+	No	Anaerobic
Ruminococcus obeum ATCC 29174	assembly	Firmicutes	No	+	No	Anaerobic
Ruminococcus torques ATCC 27756	assembly	Firmicutes	No	+	No	Anaerobic
Saccharomonospora viridis DSM 43017	complete	Actinobacteria	Yes			
Salmonella enterica subsp. enterica serovar Kentucky str. CVM29188	assembly	Gammaproteobacteria		-	Yes	Facultative
Salmonella enterica subsp. enterica serovar Virchow str. SL491	assembly	Gammaproteobacteria		-	Yes	Facultative
Sebaldella termitidis ATCC 33386	assembly	Fusobacteria	No	-	No	Anaerobic
Selenomonas flueggei ATCC 43531	assembly	Firmicutes				
Shuttleworthia satelles DSM 14600	assembly	Firmicutes				
Sideroxydans lithotrophicus ES-1	assembly	Gammaproteobacteria				
Slackia heliotrinireducens DSM 20476	complete	Actinobacteria	No	+	No	Anaerobic
Sphaerobacter thermophilus DSM 20745	assembly	Chloroflexi	No	+		
Sphingobacterium spiritivorum ATCC 33300	assembly	Bacteroidetes/Chlorobi				
Sphingobacterium spiritivorum ATCC 33861	assembly	Bacteroidetes/Chlorobi				
Sphingomonas sp. SKA58	assembly	Alphaproteobacteria		-		Aerobic
Spirosoma linguale DSM 74	assembly	Bacteroidetes/Chlorobi				
Stackebrandtia nassauensis DSM 44728	assembly	Actinobacteria		+	No	Aerobic
Staphylococcus aureus subsp. aureus MN8	assembly	Firmicutes				
Staphylococcus aureus subsp. aureus TCH130	assembly	Firmicutes				
Staphylococcus aureus subsp. aureus TCH60	assembly	Firmicutes				
Staphylococcus aureus subsp. aureus TCH70	assembly	Firmicutes				
Staphylococcus aureus subsp. aureus USA300_TCH959	assembly	Firmicutes	No	+	No	Facultative
Staphylococcus epidermidis BCM-HMP0060	assembly	Firmicutes				
Staphylococcus epidermidis M23864:W1	assembly	Firmicutes				
Staphylococcus epidermidis W23144	assembly	Firmicutes				
Staphylococcus warneri L37603	assembly	Firmicutes				
Stigmatella aurantiaca DW4/3-1	assembly	Deltaproteobacteria	Yes	-	Yes	Aerobic
Streptococcus dysgalactiae subsp. equisimilis GGS_124	complete	Firmicutes	No	+	No	
Streptococcus infantarius subsp. infantarius ATCC BAA-102	assembly	Firmicutes	No	+	No	Facultative
Streptococcus mutans NN2025	unfinished	Firmicutes	No	+	No	Facultative
Streptococcus pneumoniae 70585	complete	Firmicutes	No	_	No	Facultative
Streptococcus pneumoniae JJA	complete	Firmicutes	No	+	No	Facultative
Streptococcus pneumoniae P1031	complete	Firmicutes	No	-	No	Facultative
Streptococcus pneumoniae Taiwan19F-14	complete	Firmicutes	No	+	No	Facultative
Streptococcus pneumoniae TCH8431/19A	assembly	Firmicutes				
Streptococcus salivarius SK126	assembly	Firmicutes				
Streptococcus suis 89/1591	assembly	Firmicutes	No	+	No	Facultative
Streptococcus suis BM407	complete	Firmicutes	No	+	No	Facultative
Streptococcus suis P1/7	complete	Firmicutes	No	+	No	Facultative
Streptococcus suis SC84	complete	Firmicutes	No	+	No	Facultative
Streptosporangium roseum DSM 43021	assembly	Actinobacteria	Yes	+		Aerobic
Subdoligranulum variabile DSM 15176	assembly	Firmicutes	No	-	No	Anaerobic
Sulfitobacter sp. EE-36	assembly	Alphaproteobacteria				
Sulfitobacter sp. NAS-14.1	assembly	Alphaproteobacteria		-	Yes	Aerobic
Sulfolobus islandicus L.S.2.15	complete	Crenarchaeota				Aerobic
Sulfolobus islandicus M.14.25	complete	Crenarchaeota				Aerobic

			phenotype categories			
name	*sequence status*	*taxonomic group*	*Endospores*	*Gram Stain*	*Motility*	*Oxygene Req.*
Sulfolobus islandicus M.16.27	complete	Crenarchaeota			No	Aerobic
Sulfolobus islandicus M.16.4	complete	Crenarchaeota	No		No	Aerobic
Sulfolobus islandicus Y.G.57.14	complete	Crenarchaeota				
Sulfolobus islandicus Y.N.15.51	complete	Crenarchaeota	No		No	
Sulfolobus solfataricus 98/2	assembly	Crenarchaeota	No		No	Aerobic
Sulfolobus solfataricus 98/2	assembly	Crenarchaeota				
Sulfurihydrogenibium azorense Az-Fu1	complete	Aquificae	No	-	Yes	Microaerophilic
Sulfurihydrogenibium yellowstonense SS-5	assembly	Aquificae	No	-	Yes	Aerobic
Sulfurospirillum deleyianum DSM 6946	assembly	Epsilonproteobacteria		-	Yes	Microaerophilic
Synechococcus sp. BL107	assembly	Cyanobacteria		-		
Synechococcus sp. RS9916	assembly	Cyanobacteria		-		
Synechococcus sp. RS9917	assembly	Cyanobacteria		-		
Synechococcus sp. WH 5701	assembly	Cyanobacteria		-		
Synechococcus sp. WH 7805	assembly	Cyanobacteria		-		
Teredinibacter turnerae T7901	complete	Gammaproteobacteria		-	Yes	Aerobic
Thermanaerovibrio acidaminovorans DSM 6589	assembly	Other Bacteria	No	-	Yes	Anaerobic
Thermoanaerobacter ethanolicus CCSD1	assembly	Firmicutes	Yes	+		Anaerobic
Thermoanaerobacter sp. X513	assembly	Firmicutes		+		Anaerobic
Thermoanaerobacter sp. X561	assembly	Firmicutes	Yes	+		Anaerobic
Thermoanaerobacterium thermosaccharolyticum DSM 571	assembly	Firmicutes	Yes	+		Anaerobic
Thermococcus gammatolerans EJ3	complete	Euryarchaeota	No		Yes	Anaerobic
Thermococcus sibiricus MM 739	complete	Euryarchaeota				
Thermomonospora curvata DSM 43183	assembly	Actinobacteria	Yes	+		Aerobic
Thermotoga naphthophila RKU-10	assembly	Thermotogae			Yes	Anaerobic
Thiomonas intermedia K12	assembly	Betaproteobacteria	No	-	Yes	Aerobic
Tolumonas auensis DSM 9187	complete	Gammaproteobacteria	No	-	No	Facultative
Tsukamurella paurometabola DSM 20162	assembly	Actinobacteria	No	+	No	
Veillonella dispar ATCC 17748	assembly	Firmicutes		-		Anaerobic
Veillonella parvula DSM 2008	assembly	Firmicutes	No	-	No	Anaerobic
Vibrio alginolyticus 12G01	assembly	Gammaproteobacteria		-	Yes	Facultative
Vibrio cholerae 12129(1)	assembly	Gammaproteobacteria	No	-	Yes	Facultative
Vibrio cholerae 623-39	assembly	Gammaproteobacteria		-	Yes	Facultative
Vibrio cholerae bv. albensis VL426	assembly	Gammaproteobacteria	No	-	Yes	Anaerobic
Vibrio cholerae BX 330286	assembly	Gammaproteobacteria	No	-	Yes	Facultative
Vibrio cholerae M66-2	complete	Gammaproteobacteria	No	-	Yes	Facultative
Vibrio cholerae MJ-1236	complete	Gammaproteobacteria	No	-	Yes	Facultative
Vibrio cholerae MZO-2	assembly	Gammaproteobacteria		-	Yes	Facultative
Vibrio cholerae NCTC 8457	assembly	Gammaproteobacteria	No	-	Yes	Facultative
Vibrio cholerae O395	complete	Gammaproteobacteria		-	Yes	Facultative
Vibrio cholerae O395	complete	Gammaproteobacteria	No	-	Yes	Facultative
Vibrio cholerae RC9	assembly	Gammaproteobacteria	No	-	Yes	Facultative
Vibrio cholerae TM 11079-80	assembly	Gammaproteobacteria	No	-	Yes	Facultative
Vibrio cholerae TMA 21	assembly	Gammaproteobacteria	No	-	Yes	Anaerobic
Vibrio harveyi HY01	assembly	Gammaproteobacteria		-	Yes	Facultative
Vibrio parahaemolyticus AQ3810	assembly	Gammaproteobacteria		-	Yes	Facultative
Vibrio sp. MED222	assembly	Gammaproteobacteria		-	Yes	Facultative
Vibrio splendidus 12B01	assembly	Gammaproteobacteria		-	Yes	Facultative
Weissella paramesenteroides ATCC 33313	assembly	Firmicutes				
Wolbachia endosymbiont of Culex quinquefasciatus JHB	assembly	Alphaproteobacteria				
Xylanimonas cellulosilytica DSM 15894	assembly	Actinobacteria		+	No	Aerobic
Yersinia aldovae ATCC 35236	assembly	Gammaproteobacteria	No	-	Yes	Facultative
Yersinia bercovieri ATCC 43970	assembly	Gammaproteobacteria		-	Yes	Facultative

Total: 443
complete/assembly/unfinished: 106/328/9
