# Supplementary material for: Predicting phenotypic traits of prokaryotes from protein domain frequencies
Source: BMC Bioinformatics. 2010 Sep 24;11:481. doi: 10.1186/1471-2105-11-481 (PMC2955703; doi:10.1186/1471-2105-11-481)
Supplement: Additional file 2 — Lists of phenotype-specific discriminative domain families. The archive "discDomains.zip" contains lists of the 50 most discriminative (indicative and counterindicative) Pfam domain families associated with the four phenotype categories "Endospores","Gram stain", "Motility" and "Oxygen Requirement" in HTML format. [file 1471-2105-11-481-S2.ZIP › DiscDomains_Endospores.html]

RLSC phenotype predicition


### Prediction performance for phenotype "Endospores":

  
Sens./Spec./Harmonic Mean: 0.949/0.945/0.946
  
auPRC/aucScore: 0.956/1.000
  
best parameter lambda: 1.000000e+03
  

### positive discriminative Pfam domains

  

| Rank | weight | # groups | Pfam-ID | Pfam description |
| --- | --- | --- | --- | --- |
| 1. | +0.008 | 3 | PF03419 | Sporulation factor SpoIIGA |
| 2. | +0.007 | 5 | PF07486 | Cell Wall Hydrolase |
| 3. | +0.007 | 1 | PF06686 | Stage III sporulation protein AC (SpoIIIAC) |
| 4. | +0.007 | 2 | PF00269 | Small, acid-soluble spore proteins, alpha/beta type |
| 5. | +0.007 | 1 | PF07873 | YabP family |
| 6. | +0.007 | 1 | PF09555 | Stage III sporulation protein AD (spore\_III\_AD) |
| 7. | +0.007 | 3 | PF00407 | Pathogenesis-related protein Bet v I family |
| 8. | +0.007 | 4 | PF00876 | Innexin |
| 9. | +0.007 | 6 | PF04672 | Protein of unknown function (DUF574) |
| 10. | +0.006 | 1 | PF04647 | Accessory gene regulator B |
| 11. | +0.006 | 2 | PF09752 | Uncharacterized conserved protein (DUF2048) |
| 12. | +0.006 | 1 | PF07875 | Coat F domain |
| 13. | +0.006 | 13 | PF05067 | Manganese containing catalase |
| 14. | +0.006 | 3 | PF05655 | Pseudomonas avirulence D protein (AvrD) |
| 15. | +0.006 | 2 | PF00477 | Small hydrophilic plant seed protein |
| 16. | +0.006 | 11 | PF00553 | Cellulose binding domain |
| 17. | +0.006 | 1 | PF07872 | Protein of unknown function (DUF1659) |
| 18. | +0.006 | 1 | PF00720 | Subtilisin inhibitor-like |
| 19. | +0.006 | 1 | PF04149 | Domain of unknown function (DUF397) |
| 20. | +0.006 | 1 | PF10764 | Inhibitor of sigma-G Gin |
| 21. | +0.006 | 2 | PF07441 | SigmaK-factor processing regulatory protein BofA |
| 22. | +0.006 | 4 | PF05761 | 5' nucleotidase family |
| 23. | +0.005 | 7 | PF03323 | Bacillus/Clostridium GerA spore germination protein |
| 24. | +0.005 | 11 | PF10025 | Uncharacterized conserved protein (DUF2267) |
| 25. | +0.005 | 1 | PF03418 | Germination protease |
| 26. | +0.005 | 3 | PF07561 | Domain of Unknown Function (DUF1540) |
| 27. | +0.005 | 1 | PF09551 | Stage II sporulation protein R (spore\_II\_R) |
| 28. | +0.005 | 1 | PF05582 | YabG peptidase U57 |
| 29. | +0.005 | 1 | PF09547 | Stage IV sporulation protein A (spore\_IV\_A) |
| 30. | +0.005 | 5 | PF06898 | Putative stage IV sporulation protein YqfD |
| 31. | +0.005 | 1 | PF09578 | Spore cortex protein YabQ (Spore\_YabQ) |
| 32. | +0.005 | 6 | PF03133 | Tubulin-tyrosine ligase family |
| 33. | +0.005 | 1 | PF05542 | Protein of unknown function (DUF760) |
| 34. | +0.005 | 6 | PF09661 | Protein of unknown function (DUF2398) |
| 35. | +0.005 | 1 | PF03862 | SpoVA protein |
| 36. | +0.005 | 3 | PF05331 | Protein of unknown function (DUF742) |
| 37. | +0.005 | 5 | PF00629 | MAM domain |
| 38. | +0.005 | 2 | PF09546 | Stage III sporulation protein AE (spore\_III\_AE) |
| 39. | +0.005 | 3 | PF07451 | Stage V sporulation protein AD (SpoVAD) |
| 40. | +0.005 | 2 | PF04885 | Stigma-specific protein, Stig1 |
| 41. | +0.005 | 3 | PF07615 | YKOF-related Family |
| 42. | +0.005 | 2 | PF01001 | Hepatitis C virus non-structural protein NS4b |
| 43. | +0.005 | 1 | PF07241 | Protein of unknown function (DUF1429) |
| 44. | +0.005 | 7 | PF10062 | Predicted secreted protein (DUF2300) |
| 45. | +0.005 | 4 | PF00397 | WW domain |
| 46. | +0.005 | 9 | PF09719 | Putative redox-active protein (C\_GCAxxG\_C\_C) |
| 47. | +0.005 | 14 | PF00704 | Glycosyl hydrolases family 18 |
| 48. | +0.005 | 7 | PF08022 | FAD-binding domain |
| 49. | +0.005 | 1 | PF07816 | Protein of unknown function (DUF1645) |
| 50. | +0.005 | 1 | PF00960 | Neocarzinostatin family |

### negative discriminative Pfam domains

  

| Rank | weight | # groups | Pfam-ID | Pfam description |
| --- | --- | --- | --- | --- |
| 1. | -0.009 | 1 | PF02966 | Mitosis protein DIM1 |
| 2. | -0.009 | 1 | PF06957 | Coatomer (COPI) alpha subunit C-terminus |
| 3. | -0.008 | 1 | PF05185 | PRMT5 arginine-N-methyltransferase |
| 4. | -0.008 | 1 | PF08767 | CRM1 C terminal |
| 5. | -0.006 | 4 | PF00429 | ENV polyprotein (coat polyprotein) |
| 6. | -0.006 | 2 | PF02718 | Herpesvirus UL31-like protein |
| 7. | -0.006 | 2 | PF04756 | OST3 / OST6 family |
| 8. | -0.006 | 1 | PF02489 | Herpesvirus glycoprotein H |
| 9. | -0.005 | 2 | PF04855 | SNF5 / SMARCB1 / INI1 |
| 10. | -0.005 | 9 | PF04303 | PrpF protein |
| 11. | -0.005 | 6 | PF08483 | IstB-like ATP binding N-terminal |
| 12. | -0.005 | 9 | PF04411 | Protein of unknown function (DUF524) |
| 13. | -0.004 | 2 | PF09738 | Double stranded RNA binding protein (DUF2051) |
| 14. | -0.004 | 8 | PF09936 | Uncharacterized protein conserved in bacteria (DUF2168) |
| 15. | -0.004 | 7 | PF06934 | Fatty acid cis/trans isomerase (CTI) |
| 16. | -0.004 | 6 | PF04165 | Protein of unknown function (DUF401) |
| 17. | -0.004 | 9 | PF04223 | Citrate lyase, alpha subunit (CitF) |
| 18. | -0.004 | 4 | PF00505 | HMG (high mobility group) box |
| 19. | -0.004 | 8 | PF10633 | NPCBM-associated, NEW3 domain of alpha-galactosidase |
| 20. | -0.004 | 6 | PF05034 | Methylaspartate ammonia-lyase N-terminus |
| 21. | -0.004 | 6 | PF07476 | Methylaspartate ammonia-lyase C-terminus |
| 22. | -0.004 | 7 | PF00607 | gag gene protein p24 (core nucleocapsid protein) |
| 23. | -0.004 | 1 | PF08191 | LRR adjacent |
| 24. | -0.004 | 1 | PF06860 | Protein of unknown function (DUF1252) |
| 25. | -0.004 | 14 | PF08780 | Nucleotidyltransferase substrate binding protein like |
| 26. | -0.004 | 18 | PF02230 | Phospholipase/Carboxylesterase |
| 27. | -0.004 | 11 | PF03806 | AbgT putative transporter family |
| 28. | -0.004 | 2 | PF09657 | CRISPR-associated protein Csx8 (Cas\_Csx8) |
| 29. | -0.004 | 1 | PF07550 | Protein of unknown function (DUF1533) |
| 30. | -0.004 | 16 | PF02550 | Acetyl-CoA hydrolase/transferase N-terminal domain |
| 31. | -0.004 | 11 | PF10099 | Uncharacterized protein conserved in bacteria (DUF2337) |
| 32. | -0.004 | 9 | PF02649 | Uncharacterized ACR, COG1469 |
| 33. | -0.004 | 10 | PF03594 | Benzoate membrane transport protein |
| 34. | -0.004 | 18 | PF01212 | Beta-eliminating lyase |
| 35. | -0.003 | 12 | PF07167 | Poly-beta-hydroxybutyrate polymerase (PhaC) N-terminus |
| 36. | -0.003 | 15 | PF05226 | CHASE2 domain |
| 37. | -0.003 | 1 | PF06225 | Poxvirus A4/B15 family |
| 38. | -0.003 | 18 | PF03328 | HpcH/HpaI aldolase/citrate lyase family |
| 39. | -0.003 | 6 | PF05583 | Albicidin resistance domain |
| 40. | -0.003 | 19 | PF07969 | Amidohydrolase family |
| 41. | -0.003 | 16 | PF04367 | Protein of unknown function (DUF502) |
| 42. | -0.003 | 10 | PF08751 | TrwC relaxase |
| 43. | -0.003 | 4 | PF10414 | Sirohaem synthase dimerisation region |
| 44. | -0.003 | 20 | PF02424 | ApbE family |
| 45. | -0.003 | 20 | PF02880 | Phosphoglucomutase/phosphomannomutase, alpha/beta/alpha domain III |
| 46. | -0.003 | 17 | PF08447 | PAS fold |
| 47. | -0.003 | 19 | PF02371 | Transposase IS116/IS110/IS902 family |
| 48. | -0.003 | 12 | PF04248 | Domain of unknown function (DUF427) |
| 49. | -0.003 | 2 | PF05718 | Poxvirus intermediate transcription factor |
| 50. | -0.003 | 9 | PF08747 | Domain of unknown function (DUF1788) |
