# Supplementary material for: Predicting phenotypic traits of prokaryotes from protein domain frequencies
Source: BMC Bioinformatics. 2010 Sep 24;11:481. doi: 10.1186/1471-2105-11-481 (PMC2955703; doi:10.1186/1471-2105-11-481)
Supplement: Additional file 2 — Lists of phenotype-specific discriminative domain families. The archive "discDomains.zip" contains lists of the 50 most discriminative (indicative and counterindicative) Pfam domain families associated with the four phenotype categories "Endospores","Gram stain", "Motility" and "Oxygen Requirement" in HTML format. [file 1471-2105-11-481-S2.ZIP › DiscDomains_GramStain.html]

RLSC phenotype predicition


### Prediction performance for phenotype "Gram\_Stain":

  
Sens./Spec./Harmonic Mean: 0.978/0.955/0.966
  
auPRC/aucScore: 0.981/1.000
  
best parameter lambda: 1.000000e+04
  

### positive discriminative Pfam domains

  

| Rank | weight | # groups | Pfam-ID | Pfam description |
| --- | --- | --- | --- | --- |
| 1. | +0.005 | 1 | PF08700 | Vps51/Vps67 |
| 2. | +0.005 | 1 | PF06878 | Pkip-1 protein |
| 3. | +0.005 | 3 | PF04893 | Yip1 domain |
| 4. | +0.004 | 3 | PF05580 | SpoIVB peptidase S55 |
| 5. | +0.004 | 19 | PF01032 | FecCD transport family |
| 6. | +0.004 | 10 | PF06778 | Chlorite dismutase |
| 7. | +0.004 | 1 | PF09504 | Bsp6I restriction endonuclease |
| 8. | +0.004 | 6 | PF01908 | Protein of unknown function DUF75 |
| 9. | +0.004 | 16 | PF00781 | Diacylglycerol kinase catalytic domain |
| 10. | +0.004 | 6 | PF08353 | Domain of unknown function (DUF1727) |
| 11. | +0.004 | 4 | PF07198 | DUF1410 domain |
| 12. | +0.004 | 3 | PF10438 | Cyclo-malto-dextrinase C-terminal domain |
| 13. | +0.004 | 7 | PF01504 | Phosphatidylinositol-4-phosphate 5-Kinase |
| 14. | +0.004 | 11 | PF03861 | ANTAR domain |
| 15. | +0.004 | 3 | PF06947 | Protein of unknown function (DUF1290) |
| 16. | +0.004 | 4 | PF10103 | Uncharacterised conserved protein (DUF2342) |
| 17. | +0.004 | 8 | PF05949 | Bacterial protein of unknown function (DUF881) |
| 18. | +0.004 | 4 | PF06153 | Protein of unknown function (DUF970) |
| 19. | +0.004 | 1 | PF08769 | Sporulation initiation factor Spo0A C terminal |
| 20. | +0.004 | 1 | PF09665 | Type II restriction endonuclease (RE\_Alw26IDE) |
| 21. | +0.004 | 4 | PF08598 | Sds3-like |
| 22. | +0.003 | 16 | PF01268 | Formate--tetrahydrofolate ligase |
| 23. | +0.003 | 13 | PF00443 | Ubiquitin carboxyl-terminal hydrolase |
| 24. | +0.003 | 2 | PF09571 | XcyI restriction endonuclease |
| 25. | +0.003 | 4 | PF09488 | Mannosyl-3-phosphoglycerate synthase (osmo\_MPGsynth) |
| 26. | +0.003 | 1 | PF06686 | Stage III sporulation protein AC (SpoIIIAC) |
| 27. | +0.003 | 17 | PF01580 | FtsK/SpoIIIE family |
| 28. | +0.003 | 9 | PF05991 | Protein of unknown function (DUF901) |
| 29. | +0.003 | 1 | PF04825 | N terminus of Rad21 / Rec8 like protein |
| 30. | +0.003 | 6 | PF00519 | Papillomavirus helicase |
| 31. | +0.003 | 2 | PF03990 | Domain of unknown function (DUF348) |
| 32. | +0.003 | 20 | PF00300 | Phosphoglycerate mutase family |
| 33. | +0.003 | 2 | PF04855 | SNF5 / SMARCB1 / INI1 |
| 34. | +0.003 | 18 | PF00909 | Ammonium Transporter Family |
| 35. | +0.003 | 8 | PF04203 | Sortase family |
| 36. | +0.003 | 10 | PF03816 | Cell envelope-related transcriptional attenuator domain |
| 37. | +0.003 | 1 | PF06781 | Uncharacterised protein family (UPF0233) |
| 38. | +0.003 | 20 | PF00486 | Transcriptional regulatory protein, C terminal |
| 39. | +0.003 | 1 | PF04569 | Protein of unknown function |
| 40. | +0.003 | 4 | PF02467 | Transcription factor WhiB |
| 41. | +0.003 | 1 | PF06135 | Bacterial protein of unknown function (DUF965) |
| 42. | +0.003 | 12 | PF02645 | Uncharacterised protein, DegV family COG1307 |
| 43. | +0.003 | 7 | PF07905 | Purine catabolism regulatory protein-like family |
| 44. | +0.003 | 13 | PF07730 | Histidine kinase |
| 45. | +0.003 | 2 | PF06257 | Protein of unknown function (DUF1021) |
| 46. | +0.003 | 11 | PF01424 | R3H domain |
| 47. | +0.003 | 1 | PF01770 | Reduced folate carrier |
| 48. | +0.003 | 5 | PF04816 | Family of unknown function (DUF633) |
| 49. | +0.003 | 4 | PF10635 | DisA bacterial checkpoint controller linker region |
| 50. | +0.003 | 16 | PF01680 | SOR/SNZ family |

### negative discriminative Pfam domains

  

| Rank | weight | # groups | Pfam-ID | Pfam description |
| --- | --- | --- | --- | --- |
| 1. | -0.005 | 8 | PF09936 | Uncharacterized protein conserved in bacteria (DUF2168) |
| 2. | -0.005 | 1 | PF07834 | RanGAP1 C-terminal domain |
| 3. | -0.004 | 2 | PF09735 | Membrane-associated apoptosis protein |
| 4. | -0.004 | 18 | PF01103 | Surface antigen |
| 5. | -0.004 | 16 | PF02550 | Acetyl-CoA hydrolase/transferase N-terminal domain |
| 6. | -0.004 | 6 | PF04165 | Protein of unknown function (DUF401) |
| 7. | -0.004 | 2 | PF08205 | CD80-like C2-set immunoglobulin domain |
| 8. | -0.004 | 1 | PF08767 | CRM1 C terminal |
| 9. | -0.004 | 1 | PF05719 | Golgi phosphoprotein 3 (GPP34) |
| 10. | -0.004 | 18 | PF00263 | Bacterial type II and III secretion system protein |
| 11. | -0.004 | 3 | PF04547 | Protein of unknown function, DUF590 |
| 12. | -0.004 | 19 | PF00691 | OmpA family |
| 13. | -0.004 | 19 | PF03739 | Predicted permease YjgP/YjgQ family |
| 14. | -0.003 | 6 | PF03648 | Glycosyl hydrolase family 67 N-terminus |
| 15. | -0.003 | 13 | PF00207 | Alpha-2-macroglobulin family |
| 16. | -0.003 | 17 | PF07244 | Surface antigen variable number repeat |
| 17. | -0.003 | 13 | PF04465 | Protein of unknown function (DUF499) |
| 18. | -0.003 | 20 | PF00529 | HlyD family secretion protein |
| 19. | -0.003 | 2 | PF02212 | Dynamin GTPase effector domain |
| 20. | -0.003 | 2 | PF07672 | Mycoplasma MFS transporter |
| 21. | -0.003 | 12 | PF10569 | Alpha-macro-globulin thiol-ester bond-forming region |
| 22. | -0.003 | 20 | PF07719 | Tetratricopeptide repeat |
| 23. | -0.003 | 17 | PF02472 | Biopolymer transport protein ExbD/TolR |
| 24. | -0.003 | 15 | PF03331 | UDP-3-O-acyl N-acetylglycosamine deacetylase |
| 25. | -0.003 | 2 | PF10383 | Transcription-silencing protein Clr2 |
| 26. | -0.003 | 17 | PF06835 | Protein of unknown function (DUF1239) |
| 27. | -0.003 | 14 | PF04413 | 3-Deoxy-D-manno-octulosonic-acid transferase (kdotransferase) |
| 28. | -0.003 | 1 | PF06646 | High affinity transport system protein p37 |
| 29. | -0.003 | 16 | PF03938 | Outer membrane protein (OmpH-like) |
| 30. | -0.003 | 13 | PF08309 | LVIVD repeat |
| 31. | -0.003 | 17 | PF02606 | Tetraacyldisaccharide-1-P 4'-kinase |
| 32. | -0.003 | 20 | PF03572 | Peptidase family S41 |
| 33. | -0.003 | 2 | PF01284 | Membrane-associating domain |
| 34. | -0.003 | 1 | PF05185 | PRMT5 arginine-N-methyltransferase |
| 35. | -0.003 | 20 | PF00158 | Sigma-54 interaction domain |
| 36. | -0.003 | 1 | PF10632 | He\_PIG associated, NEW1 domain of bacterial glycohydrolase |
| 37. | -0.003 | 19 | PF01618 | MotA/TolQ/ExbB proton channel family |
| 38. | -0.003 | 19 | PF01075 | Glycosyltransferase family 9 (heptosyltransferase) |
| 39. | -0.003 | 10 | PF07678 | A-macroglobulin complement component |
| 40. | -0.003 | 1 | PF06603 | Protein of unknown function (DUF1141) |
| 41. | -0.003 | 19 | PF00342 | Phosphoglucose isomerase |
| 42. | -0.003 | 20 | PF00515 | Tetratricopeptide repeat |
| 43. | -0.003 | 20 | PF00873 | AcrB/AcrD/AcrF family |
| 44. | -0.003 | 17 | PF01339 | CheB methylesterase |
| 45. | -0.003 | 2 | PF04258 | Signal peptide peptidase |
| 46. | -0.003 | 1 | PF05470 | Eukaryotic translation initiation factor 3 subunit 8 N-terminus (eIF3c\_N) |
| 47. | -0.003 | 1 | PF00600 | Influenza non-structural protein (NS1) |
| 48. | -0.003 | 20 | PF02800 | Glyceraldehyde 3-phosphate dehydrogenase, C-terminal domain |
| 49. | -0.003 | 15 | PF04613 | UDP-3-O-[3-hydroxymyristoyl] glucosamine N-acyltransferase, LpxD |
| 50. | -0.003 | 20 | PF02321 | Outer membrane efflux protein |
