# Supplementary material for: Predicting phenotypic traits of prokaryotes from protein domain frequencies
Source: BMC Bioinformatics. 2010 Sep 24;11:481. doi: 10.1186/1471-2105-11-481 (PMC2955703; doi:10.1186/1471-2105-11-481)
Supplement: Additional file 2 — Lists of phenotype-specific discriminative domain families. The archive "discDomains.zip" contains lists of the 50 most discriminative (indicative and counterindicative) Pfam domain families associated with the four phenotype categories "Endospores","Gram stain", "Motility" and "Oxygen Requirement" in HTML format. [file 1471-2105-11-481-S2.ZIP › DiscDomains_Motility.html]

RLSC phenotype predicition


### Prediction performance for phenotype "Motility":

  
Sens./Spec./Harmonic Mean: 0.948/0.916/0.932
  
auPRC/aucScore: 0.964/0.976
  
best parameter lambda: 1.000000e+04
  

### positive discriminative Pfam domains

  

| Rank | weight | # groups | Pfam-ID | Pfam description |
| --- | --- | --- | --- | --- |
| 1. | +0.008 | 18 | PF00015 | Methyl-accepting chemotaxis protein (MCP) signaling domain |
| 2. | +0.007 | 20 | PF00672 | HAMP domain |
| 3. | +0.007 | 14 | PF00460 | Flagella basal body rod protein |
| 4. | +0.006 | 12 | PF08345 | Flagellar M-ring protein C-terminal |
| 5. | +0.006 | 15 | PF06429 | Domain of unknown function (DUF1078) |
| 6. | +0.006 | 14 | PF00700 | Bacterial flagellin C-terminus |
| 7. | +0.006 | 16 | PF01312 | FlhB HrpN YscU SpaS Family |
| 8. | +0.006 | 11 | PF02120 | Flagellar hook-length control protein |
| 9. | +0.006 | 14 | PF02049 | Flagellar hook-basal body complex protein FliE |
| 10. | +0.006 | 14 | PF00669 | Bacterial flagellin N-terminus |
| 11. | +0.006 | 15 | PF01052 | Surface presentation of antigens (SPOA) |
| 12. | +0.006 | 19 | PF08666 | SAF domain |
| 13. | +0.006 | 14 | PF02154 | Flagellar motor switch protein FliM |
| 14. | +0.006 | 14 | PF02743 | Cache domain |
| 15. | +0.006 | 14 | PF03963 | Flagellar hook capping protein |
| 16. | +0.006 | 16 | PF07195 | Flagellar hook-associated protein 2 C-terminus |
| 17. | +0.006 | 13 | PF03748 | Flagellar basal body-associated protein FliL |
| 18. | +0.006 | 8 | PF03646 | FlaG protein |
| 19. | +0.006 | 15 | PF00813 | FliP family |
| 20. | +0.006 | 14 | PF02107 | Flagellar L-ring protein |
| 21. | +0.006 | 16 | PF02895 | Signal transducing histidine kinase, homodimeric domain |
| 22. | +0.005 | 14 | PF02465 | Flagellar hook-associated protein 2 C-terminus |
| 23. | +0.005 | 16 | PF04552 | Sigma-54, DNA binding domain |
| 24. | +0.005 | 17 | PF00771 | FHIPEP family |
| 25. | +0.005 | 17 | PF01584 | CheW-like domain |
| 26. | +0.005 | 12 | PF00309 | Sigma-54 factor, Activator interacting domain (AID) |
| 27. | +0.005 | 15 | PF01514 | Secretory protein of YscJ/FliF family |
| 28. | +0.005 | 15 | PF01313 | Bacterial export proteins, family 3 |
| 29. | +0.005 | 11 | PF04316 | Anti-sigma-28 factor, FlgM |
| 30. | +0.005 | 14 | PF07559 | Flagellar basal body protein FlaE |
| 31. | +0.005 | 14 | PF01706 | FliG C-terminal domain |
| 32. | +0.005 | 14 | PF02561 | Flagellar protein FliS |
| 33. | +0.005 | 13 | PF02108 | Flagellar assembly protein FliH |
| 34. | +0.005 | 10 | PF04964 | Flp/Fap pilin component |
| 35. | +0.005 | 16 | PF01311 | Bacterial export proteins, family 1 |
| 36. | +0.005 | 15 | PF02119 | Flagellar P-ring protein |
| 37. | +0.005 | 2 | PF04200 | Lipoprotein associated domain |
| 38. | +0.005 | 13 | PF04509 | CheC-like family |
| 39. | +0.005 | 19 | PF03102 | NeuB family |
| 40. | +0.004 | 2 | PF05377 | Flagella accessory protein C (FlaC) |
| 41. | +0.004 | 17 | PF04963 | Sigma-54 factor, core binding domain |
| 42. | +0.004 | 1 | PF02816 | Alpha-kinase family |
| 43. | +0.004 | 6 | PF02050 | Flagellar FliJ protein |
| 44. | +0.004 | 4 | PF00427 | Phycobilisome Linker polypeptide |
| 45. | +0.004 | 20 | PF00027 | Cyclic nucleotide-binding domain |
| 46. | +0.004 | 5 | PF07194 | P2 response regulator binding domain |
| 47. | +0.004 | 7 | PF05682 | Phosphorylase kinase alpha/beta |
| 48. | +0.004 | 9 | PF05130 | FlgN protein |
| 49. | +0.004 | 12 | PF07196 | Flagellin hook IN motif |
| 50. | +0.004 | 2 | PF04396 | Protein of unknown function, DUF537 |

### negative discriminative Pfam domains

  

| Rank | weight | # groups | Pfam-ID | Pfam description |
| --- | --- | --- | --- | --- |
| 1. | -0.007 | 1 | PF07649 | C1-like domain |
| 2. | -0.007 | 9 | PF07040 | Protein of unknown function (DUF1326) |
| 3. | -0.006 | 1 | PF07921 | Fibritin C-terminal region |
| 4. | -0.006 | 1 | PF05038 | Cytochrome Cytochrome b558 alpha-subunit |
| 5. | -0.006 | 1 | PF03805 | Cytoadherence-linked asexual protein |
| 6. | -0.006 | 2 | PF10455 | Bin/amphiphysin/Rvs domain for vesicular trafficking |
| 7. | -0.005 | 1 | PF07056 | Protein of unknown function (DUF1335) |
| 8. | -0.005 | 4 | PF03122 | Herpes virus major capsid protein |
| 9. | -0.005 | 9 | PF01442 | Apolipoprotein A1/A4/E domain |
| 10. | -0.005 | 1 | PF03164 | Trafficking protein Mon1 |
| 11. | -0.005 | 2 | PF06151 | Trehalose receptor |
| 12. | -0.005 | 1 | PF08079 | Ribosomal L30 N-terminal domain |
| 13. | -0.005 | 2 | PF02718 | Herpesvirus UL31-like protein |
| 14. | -0.005 | 8 | PF09865 | Predicted periplasmic protein (DUF2092) |
| 15. | -0.005 | 2 | PF03345 | Dolichyl-diphosphooligosaccharide-protein glycosyltransferase 48kD subunit |
| 16. | -0.005 | 1 | PF01801 | Cytomegalovirus glycoprotein L |
| 17. | -0.005 | 1 | PF04709 | Anti-Mullerian hormone, N terminal region |
| 18. | -0.005 | 2 | PF08616 | Spindle pole body interacting protein |
| 19. | -0.005 | 1 | PF05020 | NPL4 family, putative zinc binding region |
| 20. | -0.005 | 6 | PF03815 | LCCL domain |
| 21. | -0.005 | 2 | PF00640 | Phosphotyrosine interaction domain (PTB/PID) |
| 22. | -0.005 | 8 | PF08770 | Sulphur oxidation protein SoxZ |
| 23. | -0.005 | 2 | PF04182 | B-block binding subunit of TFIIIC |
| 24. | -0.005 | 1 | PF10434 | Monopolin complex protein MAM1 |
| 25. | -0.005 | 1 | PF09729 | Gti1/Pac2 family |
| 26. | -0.005 | 2 | PF02076 | Pheromone A receptor |
| 27. | -0.005 | 4 | PF03514 | GRAS family transcription factor |
| 28. | -0.005 | 1 | PF00002 | 7 transmembrane receptor (Secretin family) |
| 29. | -0.005 | 2 | PF07930 | D-aminopeptidase, domain B |
| 30. | -0.005 | 1 | PF08098 | Anemonia sulcata toxin III family |
| 31. | -0.005 | 5 | PF01403 | Sema domain |
| 32. | -0.005 | 2 | PF10303 | Protein of unknown function (DUF2408) |
| 33. | -0.005 | 1 | PF04759 | Protein of unknown function, DUF617 |
| 34. | -0.005 | 7 | PF09948 | Predicted metal-binding integral membrane protein (DUF2182) |
| 35. | -0.005 | 2 | PF05887 | Procyclic acidic repetitive protein (PARP) |
| 36. | -0.004 | 2 | PF02116 | Fungal pheromone mating factor STE2 GPCR |
| 37. | -0.004 | 1 | PF05308 | Protein of unknown function (DUF729) |
| 38. | -0.004 | 1 | PF02207 | Putative zinc finger in N-recognin (UBR box) |
| 39. | -0.004 | 2 | PF04855 | SNF5 / SMARCB1 / INI1 |
| 40. | -0.004 | 3 | PF05428 | Corticotropin-releasing factor binding protein (CRF-BP) |
| 41. | -0.004 | 4 | PF03013 | Pyrimidine dimer DNA glycosylase |
| 42. | -0.004 | 10 | PF04383 | KilA-N domain |
| 43. | -0.004 | 15 | PF04140 | Isoprenylcysteine carboxyl methyltransferase (ICMT) family |
| 44. | -0.004 | 9 | PF08902 | Domain of unknown function (DUF1848) |
| 45. | -0.004 | 2 | PF00061 | Lipocalin / cytosolic fatty-acid binding protein family |
| 46. | -0.004 | 2 | PF02964 | Methane monooxygenase, hydrolase gamma chain |
| 47. | -0.004 | 1 | PF06734 | UL97 |
| 48. | -0.004 | 12 | PF07876 | Stress responsive A/B Barrel Domain |
| 49. | -0.004 | 2 | PF04152 | Mre11 DNA-binding presumed domain |
| 50. | -0.004 | 1 | PF07149 | Pes-10 |
