# Supplementary material for: Predicting phenotypic traits of prokaryotes from protein domain frequencies
Source: BMC Bioinformatics. 2010 Sep 24;11:481. doi: 10.1186/1471-2105-11-481 (PMC2955703; doi:10.1186/1471-2105-11-481)
Supplement: Additional file 2 — Lists of phenotype-specific discriminative domain families. The archive "discDomains.zip" contains lists of the 50 most discriminative (indicative and counterindicative) Pfam domain families associated with the four phenotype categories "Endospores","Gram stain", "Motility" and "Oxygen Requirement" in HTML format. [file 1471-2105-11-481-S2.ZIP › DiscDomains_OxygenRequirement.html]

RLSC phenotype predicition


### Prediction performance for phenotype "OxygenRequirement":

  
Sens./Spec./Harmonic Mean: 0.988/0.962/0.975
  
auPRC/aucScore: 0.985/1.000
  
best parameter lambda: 1.000000e+03
  

### positive discriminative Pfam domains

  

| Rank | weight | # groups | Pfam-ID | Pfam description |
| --- | --- | --- | --- | --- |
| 1. | +0.006 | 10 | PF06778 | Chlorite dismutase |
| 2. | +0.005 | 5 | PF09941 | Uncharacterized conserved protein (DUF2173) |
| 3. | +0.005 | 16 | PF07683 | Cobalamin synthesis protein cobW C-terminal domain |
| 4. | +0.005 | 19 | PF00081 | Iron/manganese superoxide dismutases, alpha-hairpin domain |
| 5. | +0.005 | 2 | PF07106 | Tat binding protein 1(TBP-1)-interacting protein (TBPIP) |
| 6. | +0.005 | 15 | PF00463 | Isocitrate lyase family |
| 7. | +0.005 | 4 | PF06425 | Partner of SLD five, PSF3 |
| 8. | +0.005 | 17 | PF02817 | e3 binding domain |
| 9. | +0.005 | 4 | PF01322 | Cytochrome C' |
| 10. | +0.005 | 18 | PF00116 | Cytochrome C oxidase subunit II, periplasmic domain |
| 11. | +0.005 | 4 | PF05557 | Mitotic checkpoint protein |
| 12. | +0.005 | 10 | PF03079 | ARD/ARD' family |
| 13. | +0.004 | 17 | PF02630 | SCO1/SenC |
| 14. | +0.004 | 9 | PF04955 | HupE / UreJ protein |
| 15. | +0.004 | 1 | PF04780 | Protein of unknown function (DUF629) |
| 16. | +0.004 | 17 | PF02737 | 3-hydroxyacyl-CoA dehydrogenase, NAD binding domain |
| 17. | +0.004 | 16 | PF00033 | Cytochrome b(N-terminal)/b6/petB |
| 18. | +0.004 | 11 | PF09969 | Uncharacterized conserved protein (DUF2203) |
| 19. | +0.004 | 18 | PF01596 | O-methyltransferase |
| 20. | +0.004 | 18 | PF01188 | Mandelate racemase / muconate lactonizing enzyme, C-terminal domain |
| 21. | +0.004 | 8 | PF06948 | Protein of unknown function (DUF1291) |
| 22. | +0.004 | 19 | PF00581 | Rhodanese-like domain |
| 23. | +0.004 | 12 | PF05982 | Domain of unknown function (DUF897) |
| 24. | +0.004 | 19 | PF02777 | Iron/manganese superoxide dismutases, C-terminal domain |
| 25. | +0.004 | 2 | PF10715 | Endoribonuclease RegB T4-bacteriophage encoded |
| 26. | +0.004 | 2 | PF09592 | Protein of unknown function (DUF2031) |
| 27. | +0.004 | 2 | PF06194 | Phage Conserved Open Reading Frame 51 |
| 28. | +0.004 | 7 | PF03206 | Nitrogen fixation protein NifW |
| 29. | +0.004 | 17 | PF02746 | Mandelate racemase / muconate lactonizing enzyme, N-terminal domain |
| 30. | +0.004 | 20 | PF00202 | Aminotransferase class-III |
| 31. | +0.004 | 18 | PF00198 | 2-oxoacid dehydrogenases acyltransferase (catalytic domain) |
| 32. | +0.004 | 8 | PF06243 | Phenylacetic acid degradation B |
| 33. | +0.004 | 17 | PF01152 | Bacterial-like globin |
| 34. | +0.004 | 16 | PF02628 | Cytochrome oxidase assembly protein |
| 35. | +0.004 | 4 | PF07682 | Sulphur oxygenase reductase |
| 36. | +0.004 | 10 | PF05724 | Thiopurine S-methyltransferase (TPMT) |
| 37. | +0.004 | 18 | PF02785 | Biotin carboxylase C-terminal domain |
| 38. | +0.004 | 18 | PF00115 | Cytochrome C and Quinol oxidase polypeptide I |
| 39. | +0.004 | 13 | PF01730 | UreF |
| 40. | +0.004 | 14 | PF09423 | PhoD-like phosphatase |
| 41. | +0.004 | 7 | PF06934 | Fatty acid cis/trans isomerase (CTI) |
| 42. | +0.004 | 3 | PF00693 | Thymidine kinase from herpesvirus |
| 43. | +0.004 | 12 | PF10070 | Uncharacterized protein conserved in bacteria (DUF2309) |
| 44. | +0.004 | 15 | PF08267 | Cobalamin-independent synthase, N-terminal domain |
| 45. | +0.004 | 7 | PF04319 | NifZ domain |
| 46. | +0.004 | 20 | PF08240 | Alcohol dehydrogenase GroES-like domain |
| 47. | +0.004 | 19 | PF00908 | dTDP-4-dehydrorhamnose 3,5-epimerase |
| 48. | +0.004 | 18 | PF02535 | ZIP Zinc transporter |
| 49. | +0.004 | 11 | PF01712 | Deoxynucleoside kinase |
| 50. | +0.004 | 20 | PF00107 | Zinc-binding dehydrogenase |

### negative discriminative Pfam domains

  

| Rank | weight | # groups | Pfam-ID | Pfam description |
| --- | --- | --- | --- | --- |
| 1. | -0.011 | 2 | PF04484 | Family of unknown function (DUF566) |
| 2. | -0.010 | 2 | PF03081 | Exo70 exocyst complex subunit |
| 3. | -0.009 | 2 | PF06610 | Protein of unknown function (DUF1144) |
| 4. | -0.008 | 4 | PF05153 | Family of unknown function (DUF706) |
| 5. | -0.007 | 13 | PF03977 | Na+-transporting methylmalonyl-CoA/oxaloacetate decarboxylase, beta subunit |
| 6. | -0.007 | 13 | PF10371 | Domain of unknown function |
| 7. | -0.006 | 5 | PF00353 | Hemolysin-type calcium-binding repeat (2 copies) |
| 8. | -0.006 | 4 | PF05772 | NinB protein |
| 9. | -0.006 | 5 | PF00233 | 3'5'-cyclic nucleotide phosphodiesterase |
| 10. | -0.005 | 11 | PF04277 | Oxaloacetate decarboxylase, gamma chain |
| 11. | -0.005 | 21 | PF04055 | Radical SAM superfamily |
| 12. | -0.005 | 13 | PF01228 | Glycine radical |
| 13. | -0.005 | 11 | PF02665 | Nitrate reductase gamma subunit |
| 14. | -0.005 | 4 | PF03935 | Beta-glucan synthesis-associated protein (SKN1) |
| 15. | -0.005 | 19 | PF01558 | Pyruvate ferredoxin/flavodoxin oxidoreductase |
| 16. | -0.005 | 1 | PF00594 | Vitamin K-dependent carboxylation/gamma-carboxyglutamic (GLA) domain |
| 17. | -0.005 | 1 | PF05455 | GvpH |
| 18. | -0.005 | 1 | PF05465 | Halobacterial gas vesicle protein C (GVPC) repeat |
| 19. | -0.005 | 1 | PF06102 | Domain of unknown function (DUF947) |
| 20. | -0.005 | 3 | PF08263 | Leucine rich repeat N-terminal domain |
| 21. | -0.005 | 17 | PF04011 | LemA family |
| 22. | -0.005 | 10 | PF01880 | Desulfoferrodoxin |
| 23. | -0.005 | 2 | PF07373 | CAMP factor (Cfa) |
| 24. | -0.005 | 16 | PF01891 | Cobalt uptake substrate-specific transmembrane region |
| 25. | -0.005 | 4 | PF08580 | Yeast cortical protein KAR9 |
| 26. | -0.005 | 21 | PF00037 | 4Fe-4S binding domain |
| 27. | -0.005 | 1 | PF05442 | Microvirus A protein |
| 28. | -0.005 | 6 | PF03051 | Peptidase C1-like family |
| 29. | -0.005 | 4 | PF00513 | Late Protein L2 |
| 30. | -0.005 | 19 | PF01855 | Pyruvate flavodoxin/ferredoxin oxidoreductase, thiamine diP-binding domain |
| 31. | -0.005 | 8 | PF02335 | Cytochrome c552 |
| 32. | -0.005 | 2 | PF01657 | Domain of unknown function DUF26 |
| 33. | -0.005 | 16 | PF00301 | Rubredoxin |
| 34. | -0.005 | 5 | PF00172 | Fungal Zn(2)-Cys(6) binuclear cluster domain |
| 35. | -0.005 | 2 | PF00477 | Small hydrophilic plant seed protein |
| 36. | -0.005 | 9 | PF05121 | Gas vesicle protein K |
| 37. | -0.005 | 1 | PF04175 | Protein of unknown function (DUF406) |
| 38. | -0.005 | 20 | PF02397 | Bacterial sugar transferase |
| 39. | -0.005 | 15 | PF00022 | Actin |
| 40. | -0.005 | 17 | PF02915 | Rubrerythrin |
| 41. | -0.005 | 1 | PF09574 | Protein of unknown function (Duf2374) |
| 42. | -0.005 | 17 | PF10418 | Iron-sulfur cluster binding domain of dihydroorotate dehydrogenase B |
| 43. | -0.005 | 2 | PF07356 | Protein of unknown function (DUF1481) |
| 44. | -0.005 | 1 | PF01489 | Geminivirus nuclear export factor BR1 |
| 45. | -0.005 | 3 | PF01603 | Protein phosphatase 2A regulatory B subunit (B56 family) |
| 46. | -0.005 | 1 | PF03634 | TCP family transcription factor |
| 47. | -0.005 | 1 | PF05562 | Cold acclimation protein WCOR413 |
| 48. | -0.005 | 3 | PF07496 | CW-type Zinc Finger |
| 49. | -0.005 | 1 | PF10510 | Phosphatidylinositol-glycan biosynthesis class S protein |
| 50. | -0.005 | 7 | PF03982 | Diacylglycerol acyltransferase |
