# Supplementary material for: Predicting phenotypic traits of prokaryotes from protein domain frequencies
Source: BMC Bioinformatics. 2010 Sep 24;11:481. doi: 10.1186/1471-2105-11-481 (PMC2955703; doi:10.1186/1471-2105-11-481)
Supplement: Additional file 3 — Clustering dendrograms of discriminative domain families. The file "Dendrograms.pdf" contains the phenotype-specific phylogenetic clustering dendrograms for the 50 most discriminative domain families. For the phenotype categories "Gram stain" and "Oxygen Requirement" dendrograms for positive and negative discriminative domains are shown, for the "Endospores" and "Motility" phenotype categories only the dendrogram associated with positive discriminative domains is shown. [file 1471-2105-11-481-S3.PDF]

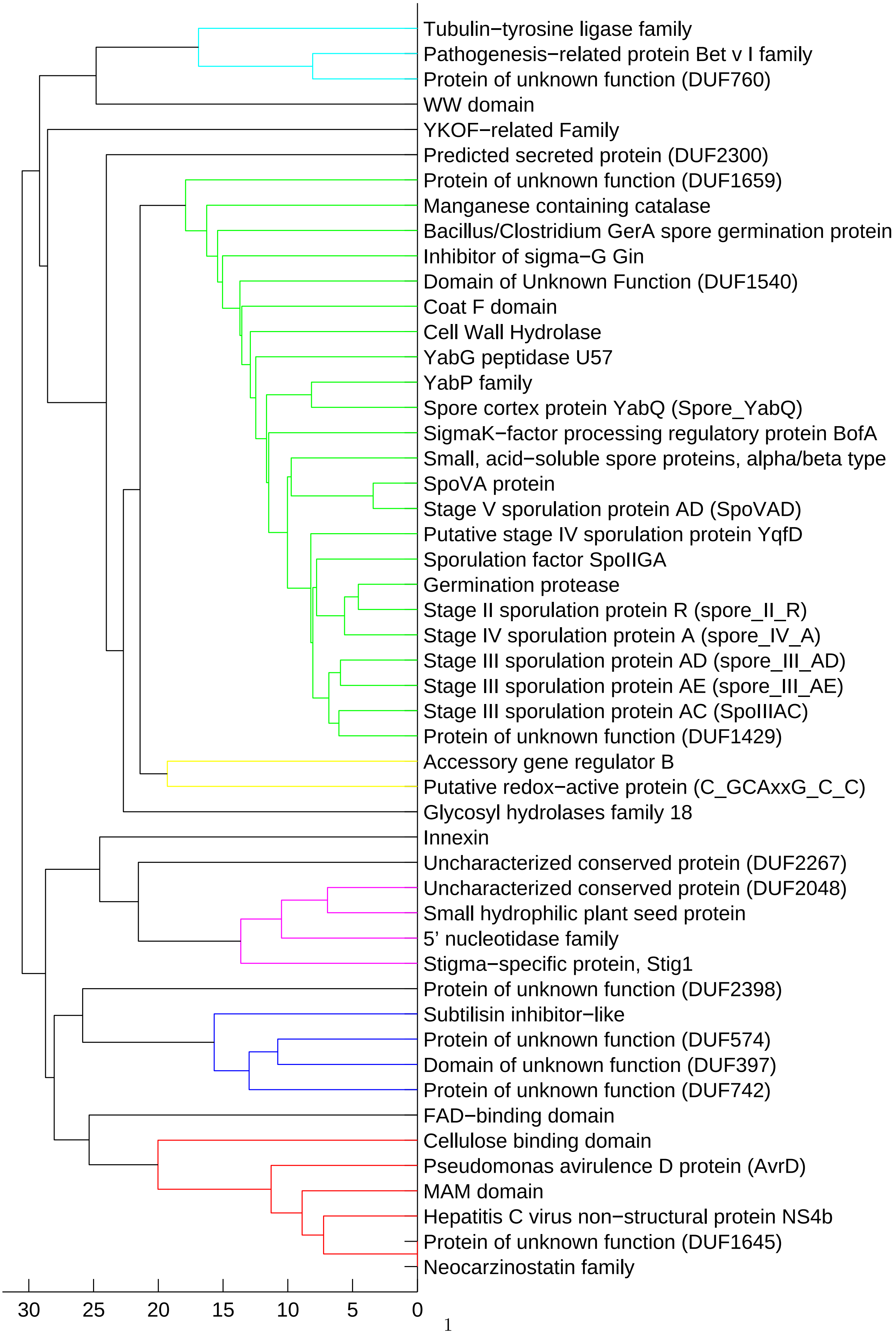

Figure 1: Clustering dendrogram for phenotype category “Endospores”

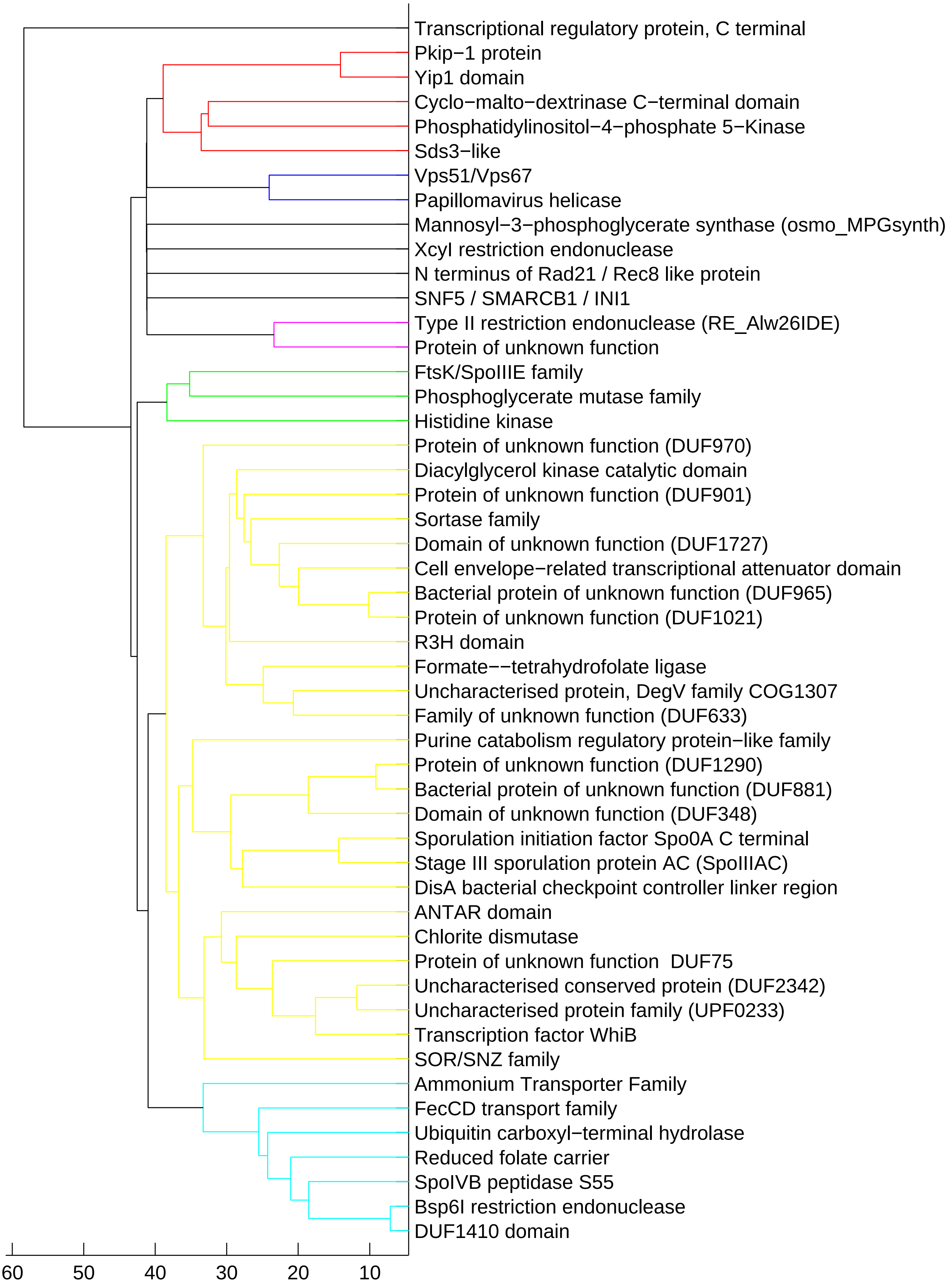

Figure 2: Clustering dendrogram for phenotype category "Gram Stain", most discriminative positive domain families

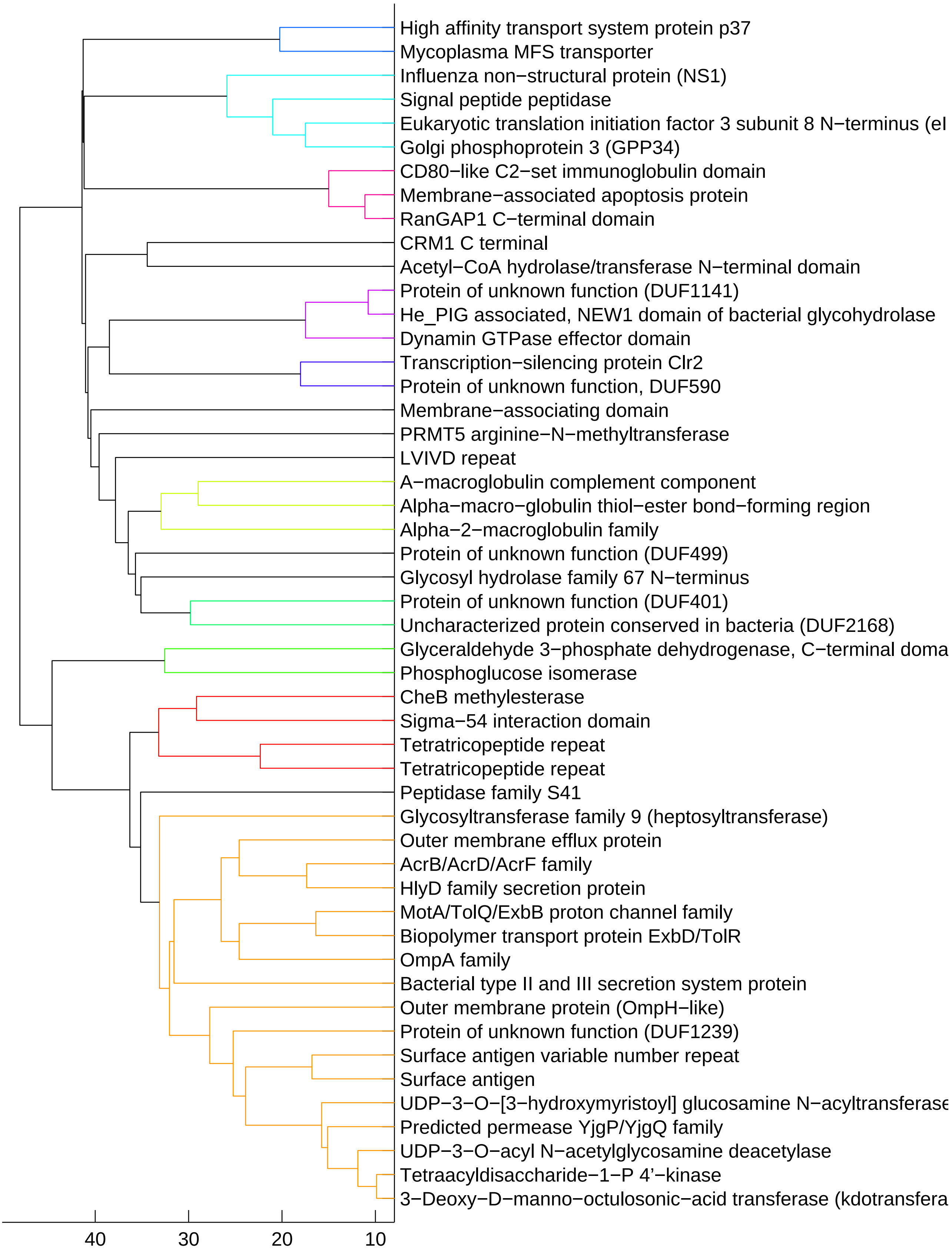

Figure 3: Clustering dendrogram for phenotype category “Gram Stain”, most discriminative negative domain families

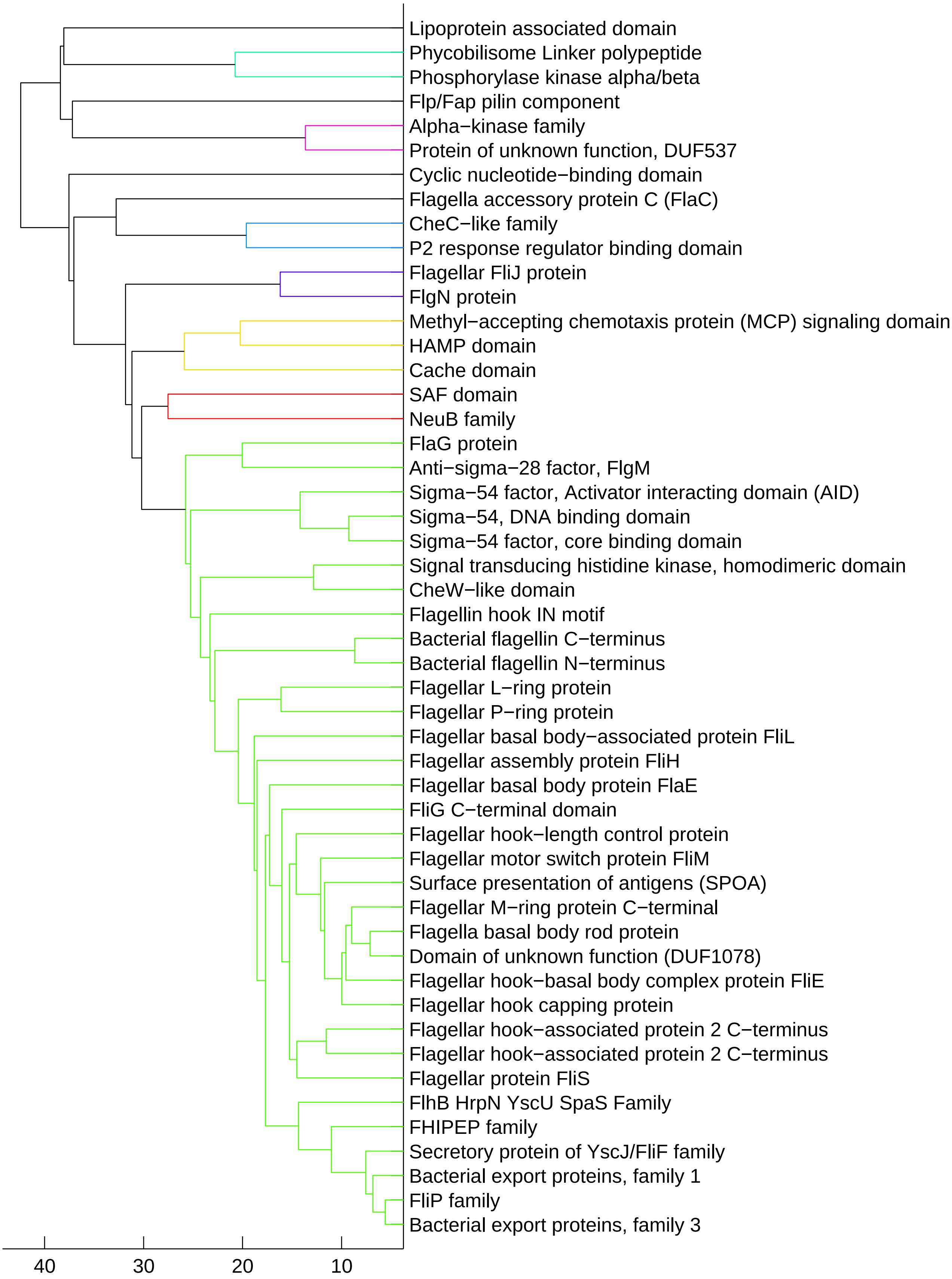

Figure 4: Clustering dendrogram for phenotype category “Motility”

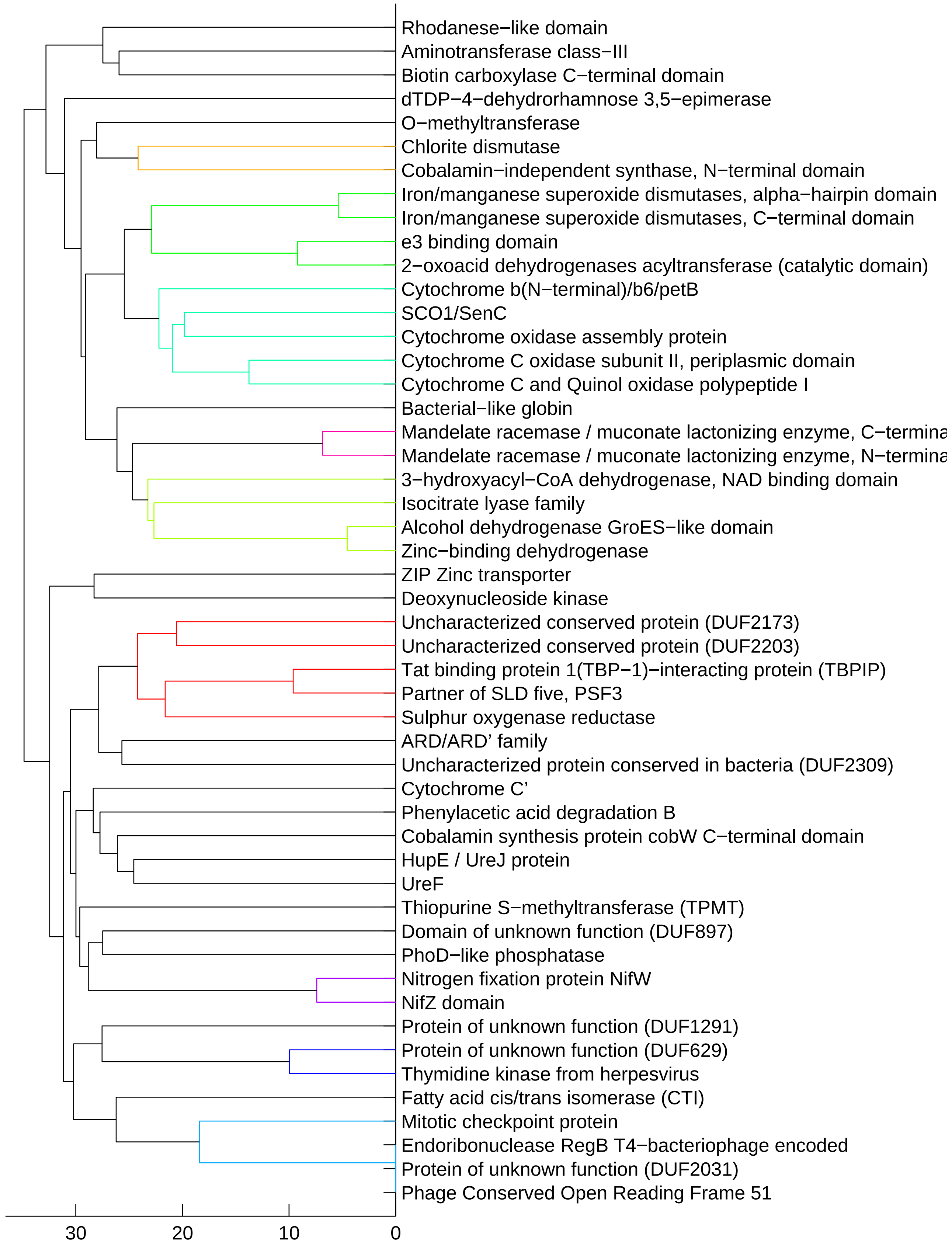

Figure 5: Clustering dendrogram for phenotype category “Oxygen Requirement”, most discriminative positive domain families

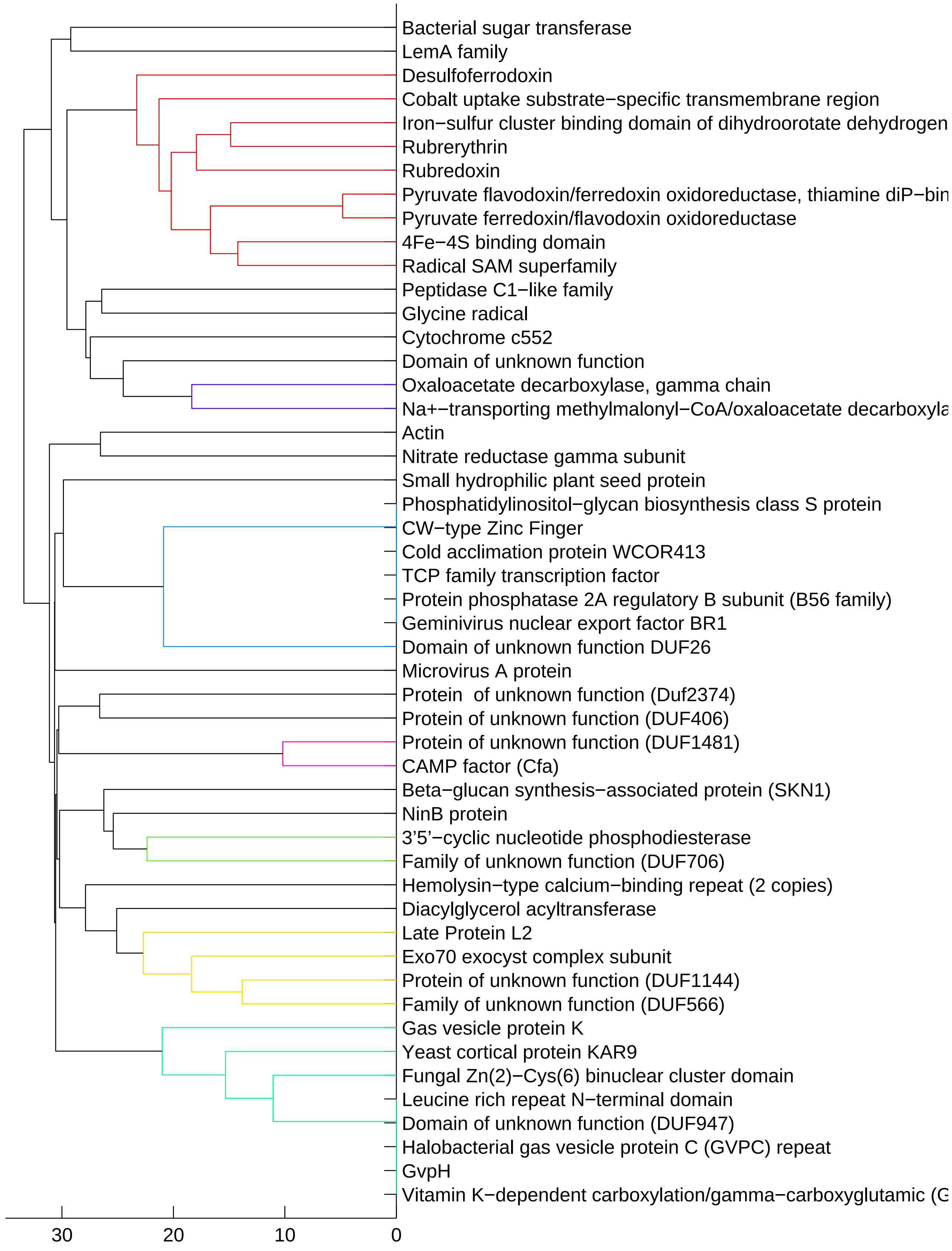

Figure 6: Clustering dendrogram for phenotype category “Oxygen Requirement”, most discriminative negative domain families
