# Supplementary material for: Predicting phenotypic traits of prokaryotes from protein domain frequencies
Source: BMC Bioinformatics. 2010 Sep 24;11:481. doi: 10.1186/1471-2105-11-481 (PMC2955703; doi:10.1186/1471-2105-11-481)
Supplement: Additional file 4 — Comparison of predicted phenotypes to NCBI annotation. The file "DeviationNCBI.pdf" contains the prediction results of the domain-based approach for the set of 443 test organisms separated according to NCBI phenotype categories for the four phenotypes used in this study. The lists contain the predicted labels as well as the original label according to the NCBI annotation; label differences are highlighted using a red cell background. Furthermore, organisms that have been identified as possibly containing a wrong NCBI phenoype annotation in section "Quality of annotation" are highlighted using a bold face type. [file 1471-2105-11-481-S4.PDF]

Deviation of prediction, sorted by phenotype

Organisms, for which annotation errors have been found by literature check are formatted with bold face.

**Endospores**	NCBI		prediction	
organism	annotation	label	label	score
Bacillus anthracis str. A0248	Yes	1	1	0.97
Bacillus cereus 03BB102	Yes	1	1	1.03
Bacillus cereus 172560W	Yes	1	1	1.04
Bacillus cereus 95/8201	Yes	1	1	0.96
Bacillus cereus AH1271	Yes	1	1	0.96
Bacillus cereus AH1272	Yes	1	1	1
Bacillus cereus AH1273	Yes	1	1	0.96
Bacillus cereus AH603	Yes	1	1	0.98
Bacillus cereus AH621	Yes	1	1	0.91
Bacillus cereus AH676	Yes	1	1	0.99
Bacillus cereus ATCC 10876	Yes	1	1	1.05
Bacillus cereus ATCC 4342	Yes	1	1	1.03
Bacillus cereus BDRD-Cer4	Yes	1	1	1
Bacillus cereus BDRD-ST196	Yes	1	1	0.93
Bacillus cereus BDRD-ST24	Yes	1	1	1.02
Bacillus cereus BDRD-ST26	Yes	1	1	0.96
Bacillus cereus F65185	Yes	1	1	1.05
Bacillus cereus H3081.97	Yes	1	1	1.02
Bacillus cereus MM3	Yes	1	1	0.99
Bacillus cereus R309803	Yes	1	1	0.84
Bacillus cereus Rock3-28	Yes	1	1	0.9
Bacillus cereus Rock3-29	Yes	1	1	0.89
Bacillus cereus Rock3-44	Yes	1	1	0.83
Bacillus cereus Rock4-18	Yes	1	1	0.88
Bacillus cereus m1293	Yes	1	1	0.97
Bacillus cereus m1550	Yes	1	1	1.1
Bacillus mycoides DSM 2048	Yes	1	1	0.88
Bacillus mycoides Rock1-4	Yes	1	1	1.07
Bacillus mycoides Rock3-17	Yes	1	1	1.07
Bacillus pseudomycoides DSM 12442	Yes	1	1	0.96
Bacillus sp. NRRL B-14911	Yes	1	1	0.78
Bacillus thuringiensis IBL 200	Yes	1	1	1.19
Bacillus thuringiensis IBL 4222	Yes	1	1	1.12
Bacillus thuringiensis serovar andalousiensis BGSC 4AW1	Yes	1	1	1.08
Bacillus thuringiensis serovar huazhongensis BGSC 4BD1	Yes	1	1	1.1
Bacillus thuringiensis serovar kurstaki str. T03a001	Yes	1	1	1.02
Bacillus thuringiensis serovar pakistani str. T13001	Yes	1	1	1
Bacillus thuringiensis serovar pondicheriensis BGSC 4BA1	Yes	1	1	1.04
Bacillus thuringiensis serovar pulsiensis BGSC 4CC1	Yes	1	1	1.06
Bacillus thuringiensis serovar sotto str. T04001	Yes	1	1	1.02
Bacillus thuringiensis serovar thuringiensis str. T01001	Yes	1	1	1
Bacillus thuringiensis serovar tochigiensis BGSC 4Y1	Yes	1	1	1.1
Bifidobacterium dentium ATCC 27678	Yes	1	-1	-0.72
Catenulispora acidiphila DSM 44928	Yes	1	1	0.52
Clostridium asparagiforme DSM 15981	Yes	1	-1	-0.21
Clostridium bartlettii DSM 16795	Yes	1	1	0.4
Clostridium bolteae ATCC BAA-613	Yes	1	1	0.04
Clostridium botulinum A2 str. Kyoto	Yes	1	1	1.03
Clostridium botulinum Ba4 str. 657	Yes	1	1	0.99
Clostridium botulinum D str. 1873	Yes	1	1	1.01
Clostridium cellulovorans 743B	Yes	1	1	0.85
Clostridium hiranonis DSM 13275	Yes	1	-1	-0.08
Clostridium kluyveri NBRC 12016	Yes	1	1	0.97
Clostridium leptum DSM 753	Yes	1	1	0.13
Clostridium nexile DSM 1787	Yes	1	1	0.37
Clostridium papyrosolvens DSM 2782	Yes	1	1	0.84
Clostridium ramosum DSM 1402	Yes	1	-1	-0.21

Clostridium scindens ATCC 35704	Yes	1	1	0.23
Clostridium sp. L2-50	Yes	1	1	0.16
Clostridium spiroforme DSM 1552	Yes	1	-1	-0.1
Geobacillus sp. Y412MC52	Yes	1	1	0.98
Kribbella flavida DSM 17836	Yes	1	1	0.18
Saccharomonospora viridis DSM 43017	Yes	1	-1	-0.09
Stigmatella aurantiaca DW4/3-1	Yes	1	1	0.79
Streptosporangium roseum DSM 43021	Yes	1	1	0.43
Thermoanaerobacter ethanolicus CCSD1	Yes	1	1	0.76
Thermoanaerobacter sp. X561	Yes	1	1	0.74
Thermoanaerobacterium thermosaccharolyticum DSM 571	Yes	1	1	0.59
Thermomonospora curvata DSM 43183	Yes	1	1	0.27
Acidimicrobium ferrooxidans DSM 10331	No	-1	-1	-0.63
Acidobacterium capsulatum ATCC 51196	No	-1	-1	-0.92
Acidovorax delafieldii 2AN	No	-1	-1	-0.94
Actinomyces odontolyticus ATCC 17982	No	-1	-1	-0.78
Alistipes putredinis DSM 17216	No	-1	-1	-0.96
Anaerococcus prevotii DSM 20548	No	-1	-1	-0.76
Anaerofustis stercorihominis DSM 17244	No	-1	-1	-0.08
Anaerostipes caccae DSM 14662	No	-1	1	0.12
Asticcacaulis excentricus CB 48	No	-1	-1	-0.95
Bacteroides coprocola DSM 17136	No	-1	-1	-0.96
Bacteroides dorei DSM 17855	No	-1	-1	-0.83
Bacteroides eggerthii DSM 20697	No	-1	-1	-0.63
Bacteroides intestinalis DSM 17393	No	-1	-1	-0.77
Bacteroides ovatus ATCC 8483	No	-1	-1	-0.97
Bacteroides pectinophilus ATCC 43243	No	-1	-1	-0.06
Bacteroides plebeius DSM 17135	No	-1	-1	-0.96
Bacteroides stercoris ATCC 43183	No	-1	-1	-0.87
Bacteroides uniformis ATCC 8492	No	-1	-1	-1.01
Bermanella marisrubri	No	-1	-1	-1.1
Beutenbergia cavernae DSM 12333	No	-1	-1	-0.35
Bifidobacterium adolescentis L2-32	No	-1	-1	-0.7
Bifidobacterium angulatum DSM 20098	No	-1	-1	-0.84
Bifidobacterium animalis subsp. lactis Bl-04	No	-1	-1	-0.97
Bifidobacterium animalis subsp. lactis DSM 10140	No	-1	-1	-0.97
Bifidobacterium breve DSM 20213	No	-1	-1	-0.88
Blautia hydrogenotrophica DSM 10507	No	-1	1	0.14
Borrelia burgdorferi 118a	No	-1	-1	-1.02
Borrelia burgdorferi 72a	No	-1	-1	-1.04
Borrelia burgdorferi 94a	No	-1	-1	-1.04
Borrelia burgdorferi Bol26	No	-1	-1	-1
Brachyspira murdochii DSM 12563	No	-1	-1	-0.91
Bryantella formatexigens DSM 14469	No	-1	1	0.1
Capnocytophaga ochracea DSM 7271	No	-1	-1	-0.97
Chitinophaga pinensis DSM 2588	No	-1	-1	-0.45
Collinsella stercoris DSM 13279	No	-1	-1	-0.79
Coprococcus comes ATCC 27758	No	-1	1	0.08
Coprococcus eutactus ATCC 27759	No	-1	-1	0
Corynebacterium aurimucosum ATCC 700975	No	-1	-1	-1.03
Corynebacterium kroppenstedtii DSM 44385	No	-1	-1	-0.93
Cryptobacterium curtum DSM 15641	No	-1	-1	-0.82
Deinococcus deserti VCD115	No	-1	-1	-0.57
Denitrovibrio acetiphilus DSM 12809	No	-1	-1	-0.85
Desulfovibrio piger ATCC 29098	No	-1	-1	-0.81
Dethiosulfovibrio peptidovorans DSM 11002	No	-1	-1	-0.94
Dickeya dadantii Ech703	No	-1	-1	-0.75
Dorea formicigenerans ATCC 27755	No	-1	1	0.13
Dorea longicatena DSM 13814	No	-1	1	0.32
Dyadobacter fermentans DSM 18053	No	-1	-1	-0.53
Edwardsiella ictaluri 93-146	No	-1	-1	-1.03
Eikenella corrodens ATCC 23834	No	-1	-1	-1.05
Erythrobacter sp. NAP1	No	-1	-1	-0.93

Escherichia coli 53638	No	-1	-1	-0.98
Escherichia coli B str. REL606	No	-1	-1	-0.98
Escherichia coli BL21	No	-1	-1	-1
Escherichia coli BW2952	No	-1	-1	-1.02
Escherichia coli O157:H7 str. TW14359	No	-1	-1	-1
Eubacterium biforme DSM 3989	No	-1	-1	-0.64
Eubacterium rectale ATCC 33656	No	-1	-1	-0.08
Faecalibacterium prausnitzii A2-165	No	-1	-1	-0.18
Faecalibacterium prausnitzii M21/2	No	-1	-1	-0.03
Fibrobacter succinogenes subsp. succinogenes S85	No	-1	-1	-0.51
Francisella novicida FTE	No	-1	-1	-1.21
Gemmatimonas aurantiaca T-27	No	-1	-1	-0.8
Halogeometricum borinquense DSM 11551	No	-1	-1	-0.68
Halorhabdus utahensis DSM 12940	No	-1	-1	-0.67
Hirschia baltica ATCC 49814	No	-1	-1	-0.94
Holdemania filiformis DSM 12042	No	-1	-1	-0.27
Hyphomicrobium denitrificans ATCC 51888	No	-1	-1	-0.9
Jonesia denitrificans DSM 20603	No	-1	-1	-0.3
Kangiella koreensis DSM 16069	No	-1	-1	-0.64
Kytococcus sedentarius DSM 20547	No	-1	-1	-0.52
Laribacter hongkongensis HLHK9	No	-1	-1	-0.67
Macrococcus caseolyticus JCSC5402	No	-1	-1	-0.52
Methylotenera mobilis JLW8	No	-1	-1	-1.05
Methylovorus sp. SIP3-4	No	-1	-1	-1.17
Mycobacterium bovis BCG str. Tokyo 172	No	-1	-1	-0.98
Mycobacterium tuberculosis KZN 1435	No	-1	-1	-0.99
Mycoplasma conjunctivae	No	-1	-1	-1.02
Mycoplasma mycoides subsp. capri str. GM12	No	-1	-1	-0.96
Neisseria sicca ATCC 29256	No	-1	-1	-0.96
Nitrosomonas sp. AL212	No	-1	-1	-1.2
Parabacteroides johnsonii DSM 18315	No	-1	-1	-0.8
Parabacteroides merdae ATCC 43184	No	-1	-1	-0.94
Parvularcula bermudensis HTCC2503	No	-1	-1	-1.15
Pedobacter heparinus DSM 2366	No	-1	-1	-0.78
Photorhabdus asymbiotica	No	-1	-1	-0.84
Prevotella copri DSM 18205	No	-1	-1	-0.9
Proteus penneri ATCC 35198	No	-1	-1	-0.98
Providencia rustigianii DSM 4541	No	-1	-1	-1.1
Providencia stuartii ATCC 25827	No	-1	-1	-1.04
Ruminococcus gnavus ATCC 29149	No	-1	1	0.33
Ruminococcus lactaris ATCC 29176	No	-1	-1	-0.04
Ruminococcus obeum ATCC 29174	No	-1	1	0.26
Ruminococcus torques ATCC 27756	No	-1	1	0.43
Sebaldella termitidis ATCC 33386	No	-1	-1	-0.24
Slackia heliotrinireducens DSM 20476	No	-1	-1	-0.55
Sphaerobacter thermophilus DSM 20745	No	-1	-1	-0.6
Staphylococcus aureus subsp. aureus USA300_TCH959	No	-1	-1	-1.02
Streptococcus dysgalactiae subsp. equisimilis GGS_124	No	-1	-1	-0.94
Streptococcus infantarius subsp. infantarius ATCC BAA-102	No	-1	-1	-0.86
Streptococcus mutans NN2025	No	-1	-1	-0.92
Streptococcus pneumoniae 70585	No	-1	-1	-0.99
Streptococcus pneumoniae JJA	No	-1	-1	-1.03
Streptococcus pneumoniae P1031	No	-1	-1	-0.99
Streptococcus pneumoniae Taiwan19F-14	No	-1	-1	-0.99
Streptococcus suis 89/1591	No	-1	-1	-0.79
Streptococcus suis BM407	No	-1	-1	-0.98
Streptococcus suis P1/7	No	-1	-1	-1.02
Streptococcus suis SC84	No	-1	-1	-0.99
Subdoligranulum variabile DSM 15176	No	-1	-1	-0.02
Sulfolobus islandicus M.16.4	No	-1	-1	-0.86
Sulfolobus islandicus Y.N.15.51	No	-1	-1	-0.77
Sulfolobus solfataricus 98/2	No	-1	-1	-0.93
Sulfurihydrogenibium azorense Az-Fu1	No	-1	-1	-0.95

Sulfurihydrogenibium yellowstonense SS-5	No	-1	-1	-0.84
Thermanaerovibrio acidaminovorans DSM 6589	No	-1	-1	-0.82
Thermococcus gammatolerans EJ3	No	-1	-1	-0.71
Thiomonas intermedia K12	No	-1	-1	-1.11
Tolumonas auensis DSM 9187	No	-1	-1	-0.94
Tsukamurella paurometabola DSM 20162	No	-1	-1	-0.61
Veillonella parvula DSM 2008	No	-1	-1	-0.76
Vibrio cholerae 12129(1)	No	-1	-1	-0.98
Vibrio cholerae BX 330286	No	-1	-1	-0.98
Vibrio cholerae M66-2	No	-1	-1	-1
Vibrio cholerae MJ-1236	No	-1	-1	-1.11
Vibrio cholerae NCTC 8457	No	-1	-1	-0.98
Vibrio cholerae O395	No	-1	-1	-1.04
Vibrio cholerae RC9	No	-1	-1	-1
Vibrio cholerae TM 11079-80	No	-1	-1	-1.04
Vibrio cholerae TMA 21	No	-1	-1	-1.03
Vibrio cholerae bv. albensis VL426	No	-1	-1	-0.97
Yersinia aldovae ATCC 35236	No	-1	-1	-1.02
Yersinia rohdei ATCC 43380	No	-1	-1	-0.99
marine actinobacterium PHSC20C1	No	-1	-1	-0.58

**Gram Stain**	NCBI		prediction	
organism	annotation	label	label	score
Acidimicrobium ferrooxidans DSM 10331	+	1	1	0.07
Actinomyces odontolyticus ATCC 17982	+	1	1	0.87
Actinosynnema mirum DSM 43827	+	1	1	0.76
Anaerococcus prevotii DSM 20548	+	1	1	0.41
Anaerofustis stercorihominis DSM 17244	+	1	1	0.53
Anaerotruncus colihominis DSM 17241	+	1	1	0.31
Bacillus anthracis str. A0248	+	1	1	0.99
Bacillus cereus 03BB102	+	1	1	0.98
Bacillus cereus 172560W	+	1	1	0.98
Bacillus cereus 95/8201	+	1	1	0.98
Bacillus cereus AH1271	+	1	1	0.98
Bacillus cereus AH1272	+	1	1	1.02
Bacillus cereus AH1273	+	1	1	1.02
Bacillus cereus AH603	+	1	1	0.95
Bacillus cereus AH621	+	1	1	0.98
Bacillus cereus AH676	+	1	1	1.01
Bacillus cereus ATCC 10876	+	1	1	0.97
Bacillus cereus ATCC 4342	+	1	1	1
Bacillus cereus BDRD-Cer4	+	1	1	1.01
Bacillus cereus BDRD-ST196	+	1	1	0.97
Bacillus cereus BDRD-ST24	+	1	1	0.95
Bacillus cereus BDRD-ST26	+	1	1	1.05
Bacillus cereus F65185	+	1	1	1.02
Bacillus cereus H3081.97	+	1	1	1.03
Bacillus cereus MM3	+	1	1	0.97
Bacillus cereus R309803	+	1	1	0.95
Bacillus cereus Rock3-28	+	1	1	0.98
Bacillus cereus Rock3-29	+	1	1	0.95
Bacillus cereus Rock3-44	+	1	1	1.01
Bacillus cereus Rock4-18	+	1	1	1.04
Bacillus cereus m1293	+	1	1	0.98
Bacillus cereus m1550	+	1	1	0.99
Bacillus mycoides DSM 2048	+	1	1	1.01
Bacillus mycoides Rock1-4	+	1	1	0.99
Bacillus mycoides Rock3-17	+	1	1	1
Bacillus pseudomycoides DSM 12442	+	1	1	0.98
Bacillus sp. NRRL B-14911	+	1	1	0.94
Bacillus thuringiensis IBL 200	+	1	1	0.97
Bacillus thuringiensis IBL 4222	+	1	1	1.07
Bacillus thuringiensis serovar andalousiensis BGSC 4AW1	+	1	1	0.99

Bacillus thuringiensis serovar huazhongensis BGSC 4BD1	+	1	1	0.97
Bacillus thuringiensis serovar kurstaki str. T03a001	+	1	1	1
Bacillus thuringiensis serovar pakistani str. T13001	+	1	1	0.99
Bacillus thuringiensis serovar pondicheriensis BGSC 4BA1	+	1	1	0.98
Bacillus thuringiensis serovar pulsiensis BGSC 4CC1	+	1	1	0.96
Bacillus thuringiensis serovar sotto str. T04001	+	1	1	1.02
Bacillus thuringiensis serovar thuringiensis str. T01001	+	1	1	0.91
Bacillus thuringiensis serovar tochigiensis BGSC 4Y1	+	1	1	1.03
Beutenbergia cavernae DSM 12333	+	1	1	0.57
Bifidobacterium adolescentis L2-32	+	1	1	0.97
Bifidobacterium angulatum DSM 20098	+	1	1	0.88
Bifidobacterium animalis subsp. lactis Bl-04	+	1	1	0.97
Bifidobacterium animalis subsp. lactis DSM 10140	+	1	1	0.97
Bifidobacterium breve DSM 20213	+	1	1	0.89
Bifidobacterium dentium ATCC 27678	+	1	1	0.9
Blautia hydrogenotrophica DSM 10507	+	1	1	0.63
Brachybacterium faecium DSM 4810	+	1	1	0.62
Brevibacillus brevis NBRC 100599	+	1	1	0.54
Bryantella formatexigens DSM 14469	+	1	1	0.64
Catenulispora acidiphila DSM 44928	+	1	1	0.62
Cellulomonas flavigena DSM 20109	+	1	1	0.75
Clostridium asparagiforme DSM 15981	+	1	1	0.32
Clostridium bartlettii DSM 16795	+	1	1	0.64
Clostridium bolteae ATCC BAA-613	+	1	1	0.35
Clostridium botulinum A2 str. Kyoto	+	1	1	1
Clostridium botulinum D str. 1873	+	1	1	0.86
Clostridium hiranonis DSM 13275	+	1	1	0.73
Clostridium hylemonae DSM 15053	+	1	1	0.54
Clostridium kluyveri NBRC 12016	+	1	1	0.83
Clostridium leptum DSM 753	+	1	1	0.83
Clostridium nexile DSM 1787	+	1	1	0.97
Clostridium ramosum DSM 1402	+	1	1	0.6
Clostridium scindens ATCC 35704	+	1	1	0.74
Clostridium sp. L2-50	+	1	1	0.74
Clostridium sp. M62/1	+	1	1	0.63
Clostridium sp. SS2/1	+	1	1	0.76
Clostridium spiroforme DSM 1552	+	1	1	0.66
Collinsella stercoris DSM 13279	+	1	1	0.7
Coprococcus comes ATCC 27758	+	1	1	0.76
Coprococcus eutactus ATCC 27759	+	1	1	0.62
Corynebacterium aurimucosum ATCC 700975	+	1	1	0.81
Corynebacterium kroppenstedtii DSM 44385	+	1	1	0.79
Corynebacterium matruchotii ATCC 14266	+	1	1	0.8
Corynebacterium tuberculostearicum SK141	+	1	1	0.82
Cryptobacterium curtum DSM 15641	+	1	1	0.16
Dorea formicigenerans ATCC 27755	+	1	1	0.7
Dorea longicatena DSM 13814	+	1	1	0.96
Enterococcus faecalis TX0104	+	1	1	1.01
Eubacterium biforme DSM 3989	+	1	1	0.68
Eubacterium dolichum DSM 3991	+	1	1	0.64
Eubacterium rectale ATCC 33656	+	1	1	0.51
Eubacterium siraeum DSM 15702	+	1	1	0.47
Eubacterium ventriosum ATCC 27560	+	1	1	0.85
Geobacillus sp. Y4.1MC1	+	1	1	0.83
Geobacillus sp. Y412MC52	+	1	1	0.99
Gordonia bronchialis DSM 43247	+	1	1	0.7
Holdemania filiformis DSM 12042	+	1	1	0.52
Janibacter sp. HTCC2649	+	1	1	0.77
Jonesia denitrificans DSM 20603	+	1	1	0.69
Kribbella flavida DSM 17836	+	1	1	0.62
Kytococcus sedentarius DSM 20547	+	1	1	0.67
Lactobacillus rhamnosus GG	+	1	1	0.88
Lactobacillus rhamnosus HN001	+	1	1	0.84

Lactobacillus rhamnosus Lc 705	+	1	1	0.89
Macrococcus caseolyticus JCSC5402	+	1	1	0.76
Methanobrevibacter smithii DSM 2375	+	1	1	0.64
Mycobacterium bovis BCG str. Tokyo 172	+	1	1	0.99
Mycobacterium tuberculosis KZN 1435	+	1	1	0.99
Parvimonas micra ATCC 33270	+	1	1	0.52
Rhodococcus erythropolis PR4	+	1	1	0.77
Rhodococcus opacus B4	+	1	1	0.71
Ruminococcus gnavus ATCC 29149	+	1	1	0.85
Ruminococcus lactaris ATCC 29176	+	1	1	0.87
Ruminococcus obeum ATCC 29174	+	1	1	0.85
Ruminococcus torques ATCC 27756	+	1	1	0.87
Slackia heliotrinireducens DSM 20476	+	1	1	0.17
Sphaerobacter thermophilus DSM 20745	+	1	-1	-0.21
Stackebrandtia nassauensis DSM 44728	+	1	1	0.55
Staphylococcus aureus subsp. aureus USA300_TCH959	+	1	1	0.92
Streptococcus dysgalactiae subsp. equisimilis GGS_124	+	1	1	0.9
Streptococcus infantarius subsp. infantarius ATCC BAA-102	+	1	1	0.98
Streptococcus mutans NN2025	+	1	1	0.98
Streptococcus pneumoniae JJA	+	1	1	0.98
Streptococcus pneumoniae Taiwan19F-14	+	1	1	0.97
Streptococcus suis 89/1591	+	1	1	1
Streptococcus suis BM407	+	1	1	1
Streptococcus suis P1/7	+	1	1	0.97
Streptococcus suis SC84	+	1	1	0.98
Streptosporangium roseum DSM 43021	+	1	1	0.67
Thermoanaerobacter ethanolicus CCSD1	+	1	1	0.73
Thermoanaerobacter sp. X513	+	1	1	0.8
Thermoanaerobacter sp. X561	+	1	1	0.78
Thermoanaerobacterium thermosaccharolyticum DSM 571	+	1	1	0.58
Thermomonospora curvata DSM 43183	+	1	1	0.77
Tsukamurella paurometabola DSM 20162	+	1	1	0.89
Xylanimonas cellulosilytica DSM 15894	+	1	1	0.67
marine actinobacterium PHSC20C1	+	1	1	0.6
Acetobacter pasteurianus IFO 3283-01	-	-1	-1	-0.88
Acetobacter pasteurianus IFO 3283-01-42C	-	-1	-1	-0.89
Acetobacter pasteurianus IFO 3283-03	-	-1	-1	-0.88
Acetobacter pasteurianus IFO 3283-07	-	-1	-1	-0.88
Acetobacter pasteurianus IFO 3283-22	-	-1	-1	-0.88
Acetobacter pasteurianus IFO 3283-26	-	-1	-1	-0.88
Acetobacter pasteurianus IFO 3283-32	-	-1	-1	-0.88
Acidobacterium capsulatum ATCC 51196	-	-1	-1	-0.24
Acidovorax delafieldii 2AN	-	-1	-1	-0.88
Actinobacillus minor 202	-	-1	-1	-0.77
Alistipes putredinis DSM 17216	-	-1	-1	-0.72
Asticcacaulis excentricus CB 48	-	-1	-1	-0.68
Azotobacter vinelandii DJ	-	-1	-1	-0.97
Bacteroides capillosus ATCC 29799	-	-1	1	0.74
Bacteroides coprocola DSM 17136	-	-1	-1	-0.11
Bacteroides dorei DSM 17855	-	-1	1	0.14
Bacteroides eggerthii DSM 20697	-	-1	-1	-0.28
Bacteroides intestinalis DSM 17393	-	-1	-1	-0.19
Bacteroides ovatus ATCC 8483	-	-1	-1	-0.3
Bacteroides pectinophilus ATCC 43243	-	-1	1	0.54
Bacteroides plebeius DSM 17135	-	-1	-1	-0.17
Bacteroides stercoris ATCC 43183	-	-1	-1	-0.46
Bacteroides uniformis ATCC 8492	-	-1	-1	-0.2
Bartonella grahamii as4aup	-	-1	-1	-1.08
Bermanella marisrubri	-	-1	-1	-1.13
Brachyspira murdochii DSM 12563	-	-1	-1	-0.49
Brucella ceti str. Cudo	-	-1	-1	-0.96
Brucella melitensis ATCC 23457	-	-1	-1	-0.96
Burkholderia glumae BGR1	-	-1	-1	-1.02

Burkholderia mallei GB8 horse 4	-	-1	-1	-1
Burkholderia mallei PRL-20	-	-1	-1	-1.03
Burkholderia pseudomallei MSHR346	-	-1	-1	-0.97
Campylobacter showae RM3277	-	-1	-1	-0.84
Candidatus Accumulibacter phosphatis clade IIA str. UW-1	-	-1	-1	-1.01
Candidatus Pelagibacter ubique HTCC1002	-	-1	-1	-0.98
Capnocytophaga ochracea DSM 7271	-	-1	-1	-0.78
Chitinophaga pinensis DSM 2588	-	-1	-1	-0.42
Chlamydia trachomatis B/Jali20/OT	-	-1	-1	-1.02
Citrobacter youngae ATCC 29220	-	-1	-1	-0.95
Clostridium cellulovorans 743B	-	-1	1	0.57
Clostridium papyrosolvens DSM 2782	-	-1	1	0.75
Croceibacter atlanticus HTCC2559	-	-1	-1	-0.84
Deinococcus deserti VCD115	-	-1	1	0.2
Denitrovibrio acetiphilus DSM 12809	-	-1	-1	-0.77
Desulfomicrobium baculatum DSM 4028	-	-1	-1	-0.87
Desulfovibrio piger ATCC 29098	-	-1	-1	-0.74
Dethiosulfovibrio peptidovorans DSM 11002	-	-1	-1	-0.35
Dickeya dadantii Ech703	-	-1	-1	-0.9
Dyadobacter fermentans DSM 18053	-	-1	-1	-0.52
Edwardsiella ictaluri 93-146	-	-1	-1	-1.02
Eikenella corrodens ATCC 23834	-	-1	-1	-1.01
Enterobacter cancerogenus ATCC 35316	-	-1	-1	-0.94
Erythrobacter sp. NAP1	-	-1	-1	-1.09
Escherichia albertii TW07627	-	-1	-1	-1.09
Escherichia coli 53638	-	-1	-1	-1.03
Escherichia coli B str. REL606	-	-1	-1	-1
Escherichia coli B171	-	-1	-1	-0.98
Escherichia coli BL21	-	-1	-1	-1.01
Escherichia coli BW2952	-	-1	-1	-1
Escherichia coli O157:H7 str. TW14359	-	-1	-1	-1
Escherichia coli O157:H7 str. TW14588	-	-1	-1	-0.99
**Eubacterium eligens ATCC 27750**	**-**	**-1**	1	**0.47**
Faecalibacterium prausnitzii A2-165	-	-1	1	0.66
Faecalibacterium prausnitzii M21/2	-	-1	1	0.63
Fibrobacter succinogenes subsp. succinogenes S85	-	-1	-1	-0.58
Francisella novicida FTE	-	-1	-1	-0.94
Fulvimarina pelagi HTCC2506	-	-1	-1	-1.05
Gemmatimonas aurantiaca T-27	-	-1	-1	-0.52
**Geodermatophilus obscurus DSM 43160**	**-**	**-1**	1	**0.55**
Haliangium ochraceum DSM 14365	-	-1	-1	-0.51
Halogeometricum borinquense DSM 11551	-	-1	-1	-0.76
Halorhabdus utahensis DSM 12940	-	-1	-1	-0.86
Helicobacter pylori B38	-	-1	-1	-0.99
Hoeflea phototrophica DFL-43	-	-1	-1	-0.92
Hyphomicrobium denitrificans ATCC 51888	-	-1	-1	-1
Idiomarina baltica OS145	-	-1	-1	-1.12
Kangiella koreensis DSM 16069	-	-1	-1	-1.2
Kordia algicida OT-1	-	-1	-1	-0.85
Laribacter hongkongensis HLHK9	-	-1	-1	-1.04
Leeuwenhoekiella blandensis MED217	-	-1	-1	-0.71
Leptotrichia buccalis DSM 1135	-	-1	-1	-0.02
Loktanella vestfoldensis SKA53	-	-1	-1	-1
Marinomonas sp. MED121	-	-1	-1	-0.84
Methanocaldococcus fervens AG86	-	-1	-1	-0.97
Methylobacterium extorquens AM1	-	-1	-1	-0.91
Methylobacterium extorquens DM4	-	-1	-1	-0.88
Methylotenera mobilis JLW8	-	-1	-1	-1.08
Methylovorus sp. SIP3-4	-	-1	-1	-1.13
Mycoplasma mycoides subsp. capri str. GM12	-	-1	-1	-0.5
Neisseria sicca ATCC 29256	-	-1	-1	-1
Neptuniibacter caesariensis	-	-1	-1	-1.03
Nitrobacter sp. Nb-311A	-	-1	-1	-0.91

Nitrosomonas sp. AL212	-	-1	-1	-1.08
Oceanicaulis alexandrii HTCC2633	-	-1	-1	-1.13
Oceanicola batsensis HTCC2597	-	-1	-1	-0.84
Oceanicola granulosus HTCC2516	-	-1	-1	-0.86
Parabacteroides johnsonii DSM 18315	-	-1	-1	-0.04
Parabacteroides merdae ATCC 43184	-	-1	1	0.02
Parvularcula bermudensis HTCC2503	-	-1	-1	-1.13
Pedobacter heparinus DSM 2366	-	-1	-1	-0.63
Phaeobacter gallaeciensis BS107	-	-1	-1	-0.96
Photobacterium angustum S14	-	-1	-1	-1.08
Photobacterium profundum 3TCK	-	-1	-1	-0.88
Photobacterium sp. SKA34	-	-1	-1	-1.01
Photorhabdus asymbiotica	-	-1	-1	-0.86
Planctomyces limnophilus DSM 3776	-	-1	-1	-0.8
Polaribacter irgensii 23-P	-	-1	-1	-0.74
Prevotella copri DSM 18205	-	-1	-1	-0.16
Proteus penneri ATCC 35198	-	-1	-1	-0.97
Providencia alcalifaciens DSM 30120	-	-1	-1	-0.92
Providencia rustigianii DSM 4541	-	-1	-1	-0.98
Providencia stuartii ATCC 25827	-	-1	-1	-0.87
Pseudoalteromonas tunicata D2	-	-1	-1	-1.04
Pseudomonas fluorescens SBW25	-	-1	-1	-0.94
Psychromonas sp. CNPT3	-	-1	-1	-1.04
Rhizobium sp. NGR234	-	-1	-1	-0.97
Rhodothermus marinus DSM 4252	-	-1	-1	-0.65
Rickettsia africae ESF-5	-	-1	-1	-1.03
Rickettsia peacockii str. Rustic	-	-1	-1	-1.02
Robiginitalea biformata HTCC2501	-	-1	-1	-0.74
Roseobacter sp. MED193	-	-1	-1	-0.87
Roseovarius nubinhibens ISM	-	-1	-1	-1.06
Roseovarius sp. 217	-	-1	-1	-1.01
Roseovarius sp. HTCC2601	-	-1	-1	-0.97
Salmonella enterica subsp. enterica serovar Kentucky str. CVM29188	-	-1	-1	-0.99
Salmonella enterica subsp. enterica serovar Virchow str. SL491	-	-1	-1	-0.99
Sebaldella termitidis ATCC 33386	-	-1	1	0.2
Sphingomonas sp. SKA58	-	-1	-1	-0.94
Stigmatella aurantiaca DW4/3-1	-	-1	-1	-0.75
Streptococcus pneumoniae P1031	-	-1	1	0.99
Subdoligranulum variabile DSM 15176	-	-1	1	0.57
Sulfitobacter sp. NAS-14.1	-	-1	-1	-0.88
Sulfurihydrogenibium azorense Az-Fu1	-	-1	-1	-0.95
Sulfurihydrogenibium yellowstonense SS-5	-	-1	-1	-0.82
Sulfurospirillum deleyianum DSM 6946	-	-1	-1	-0.71
Synechococcus sp. BL107	-	-1	-1	-1.04
Synechococcus sp. RS9916	-	-1	-1	-1.05
Synechococcus sp. RS9917	-	-1	-1	-0.87
Synechococcus sp. WH 5701	-	-1	-1	-0.95
Synechococcus sp. WH 7805	-	-1	-1	-1
Teredinibacter turnerae T7901	-	-1	-1	-0.91
Thermanaerovibrio acidaminovorans DSM 6589	-	-1	-1	-0.34
Thiomonas intermedia K12	-	-1	-1	-0.82
Tolumonas auensis DSM 9187	-	-1	-1	-0.7
Veillonella dispar ATCC 17748	-	-1	-1	-0.02
Veillonella parvula DSM 2008	-	-1	-1	-0.09
Vibrio alginolyticus 12G01	-	-1	-1	-0.95
Vibrio cholerae 12129(1)	-	-1	-1	-1.05
Vibrio cholerae 623-39	-	-1	-1	-1.01
Vibrio cholerae BX 330286	-	-1	-1	-0.99
Vibrio cholerae M66-2	-	-1	-1	-1.01
Vibrio cholerae MJ-1236	-	-1	-1	-1.06
Vibrio cholerae MZO-2	-	-1	-1	-0.99
Vibrio cholerae NCTC 8457	-	-1	-1	-1.01
Vibrio cholerae O395	-	-1	-1	-1.02

Vibrio cholerae RC9	-	-1	-1	-0.99
Vibrio cholerae TM 11079-80	-	-1	-1	-1.03
Vibrio cholerae TMA 21	-	-1	-1	-1.03
Vibrio cholerae bv. albensis VL426	-	-1	-1	-1.02
Vibrio harveyi HY01	-	-1	-1	-0.99
Vibrio parahaemolyticus AQ3810	-	-1	-1	-0.96
Vibrio sp. MED222	-	-1	-1	-1.01
Vibrio splendidus 12B01	-	-1	-1	-0.91
Yersinia aldovae ATCC 35236	-	-1	-1	-0.95
Yersinia bercovieri ATCC 43970	-	-1	-1	-0.96
Yersinia frederiksenii ATCC 33641	-	-1	-1	-0.89
Yersinia intermedia ATCC 29909	-	-1	-1	-0.88
Yersinia mollaretii ATCC 43969	-	-1	-1	-0.96
Yersinia pestis CA88-4125	-	-1	-1	-1.03
Yersinia pestis Pestoides A	-	-1	-1	-1.03
Yersinia pestis biovar Orientalis str. IP275	-	-1	-1	-1.01
Yersinia pestis biovar Orientalis str. PEXU2	-	-1	-1	-1
Yersinia rohdei ATCC 43380	-	-1	-1	-1.06
Zymomonas mobilis subsp. mobilis ATCC 10988	-	-1	-1	-0.99

**Motility**	NCBI		prediction	
organism	annotation	label	label	score
Acidovorax delafieldii 2AN	Yes	1	1	0.31
Asticcacaulis excentricus CB 48	Yes	1	1	0.86
Azotobacter vinelandii DJ	Yes	1	1	0.8
Bacillus anthracis str. A0248	Yes	1	1	0.9
Bacillus cereus 172560W	Yes	1	1	0.92
Bacillus cereus 95/8201	Yes	1	1	0.9
Bacillus cereus AH1271	Yes	1	1	0.94
Bacillus cereus AH1272	Yes	1	1	0.86
Bacillus cereus AH1273	Yes	1	1	0.86
Bacillus cereus AH603	Yes	1	1	0.9
Bacillus cereus AH621	Yes	1	1	0.85
Bacillus cereus AH676	Yes	1	1	0.95
Bacillus cereus ATCC 10876	Yes	1	1	0.99
Bacillus cereus ATCC 4342	Yes	1	1	0.95
Bacillus cereus BDRD-Cer4	Yes	1	1	0.95
Bacillus cereus BDRD-ST196	Yes	1	1	0.76
Bacillus cereus BDRD-ST24	Yes	1	1	0.99
Bacillus cereus BDRD-ST26	Yes	1	1	0.9
Bacillus cereus F65185	Yes	1	1	0.81
Bacillus cereus H3081.97	Yes	1	1	1.01
Bacillus cereus MM3	Yes	1	1	0.9
Bacillus cereus R309803	Yes	1	1	0.85
Bacillus cereus Rock3-28	Yes	1	1	0.95
Bacillus cereus Rock3-29	Yes	1	1	0.93
Bacillus cereus Rock3-44	Yes	1	1	0.58
Bacillus cereus Rock4-18	Yes	1	1	0.69
Bacillus cereus m1293	Yes	1	1	0.95
Bacillus cereus m1550	Yes	1	1	0.98
Bacillus thuringiensis IBL 200	Yes	1	1	1.06
Bacillus thuringiensis IBL 4222	Yes	1	1	0.98
Bacillus thuringiensis serovar andalousiensis BGSC 4AW1	Yes	1	1	1.01
Bacillus thuringiensis serovar huazhongensis BGSC 4BD1	Yes	1	1	0.94
Bacillus thuringiensis serovar kurstaki str. T03a001	Yes	1	1	0.96
Bacillus thuringiensis serovar pakistani str. T13001	Yes	1	1	0.96
Bacillus thuringiensis serovar pondicheriensis BGSC 4BA1	Yes	1	1	0.94
Bacillus thuringiensis serovar pulsiensis BGSC 4CC1	Yes	1	1	0.97
Bacillus thuringiensis serovar sotto str. T04001	Yes	1	1	1.06
Bacillus thuringiensis serovar thuringiensis str. T01001	Yes	1	1	0.88
Bacillus thuringiensis serovar tochigiensis BGSC 4Y1	Yes	1	1	0.94
Bermanella marisrubri	Yes	1	1	0.56
Borrelia burgdorferi 118a	Yes	1	1	1.09
Borrelia burgdorferi 29805	Yes	1	1	1.04
Borrelia burgdorferi 64b	Yes	1	1	1.09

Borrelia burgdorferi 72a	Yes	1	1	1.08
Borrelia burgdorferi 94a	Yes	1	1	1.07
Borrelia burgdorferi Bol26	Yes	1	1	1.1
Borrelia burgdorferi WI91-23	Yes	1	1	1.13
Borrelia garinii Far04	Yes	1	1	1.03
Borrelia garinii PBr	Yes	1	1	1.03
Borrelia sp. SV1	Yes	1	1	1.06
Borrelia spielmanii A14S	Yes	1	1	1
Brachyspira murdochii DSM 12563	Yes	1	1	0.33
Brevibacillus brevis NBRC 100599	Yes	1	1	0.64
Burkholderia pseudomallei MSHR346	Yes	1	1	0.37
Capnocytophaga ochracea DSM 7271	Yes	1	-1	-0.27
Cellulomonas flavigena DSM 20109	Yes	1	-1	-0.68
Chitinophaga pinensis DSM 2588	Yes	1	1	0.19
Clostridium bolteae ATCC BAA-613	Yes	1	1	0.19
Clostridium botulinum A2 str. Kyoto	Yes	1	1	0.93
Clostridium botulinum Ba4 str. 657	Yes	1	1	1.04
Clostridium botulinum D str. 1873	Yes	1	1	0.76
Clostridium kluyveri NBRC 12016	Yes	1	1	0.81
Clostridium leptum DSM 753	Yes	1	-1	-0.03
Clostridium scindens ATCC 35704	Yes	1	1	0.06
Denitrovibrio acetiphilus DSM 12809	Yes	1	1	0.36
Desulfovibrio piger ATCC 29098	Yes	1	1	0.33
Dethiosulfovibrio peptidovorans DSM 11002	Yes	1	-1	-0.08
Dickeya dadantii Ech703	Yes	1	1	0.76
Edwardsiella ictaluri 93-146	Yes	1	1	0.5
Eikenella corrodens ATCC 23834	Yes	1	-1	-0.48
Erythrobacter sp. NAP1	Yes	1	1	0.88
Escherichia coli 53638	Yes	1	1	0.97
Escherichia coli B str. REL606	Yes	1	1	0.89
Escherichia coli B171	Yes	1	1	0.95
Escherichia coli BL21	Yes	1	1	0.9
Escherichia coli BW2952	Yes	1	1	0.96
Escherichia coli O157:H7 str. TW14359	Yes	1	1	1.02
Escherichia coli O157:H7 str. TW14588	Yes	1	1	0.95
Gemmatimonas aurantiaca T-27	Yes	1	1	0.08
Geobacillus sp. Y412MC52	Yes	1	1	0.47
Haliangium ochraceum DSM 14365	Yes	1	1	0.26
Halogeometricum borinquense DSM 11551	Yes	1	1	0.55
Halomicrobium mukohataei DSM 12286	Yes	1	1	0.52
Halorhabdus utahensis DSM 12940	Yes	1	1	0.73
Helicobacter pylori B38	Yes	1	1	0.97
Hirschia baltica ATCC 49814	Yes	1	1	0.7
Hoeflea phototrophica DFL-43	Yes	1	1	0.39
Hyphomicrobium denitrificans ATCC 51888	Yes	1	1	0.36
Idiomarina baltica OS145	Yes	1	1	0.76
Jonesia denitrificans DSM 20603	Yes	1	-1	-0.26
Laribacter hongkongensis HLHK9	Yes	1	1	0.53
Marinomonas sp. MED121	Yes	1	1	0.55
Methylobacterium extorquens AM1	Yes	1	1	0.86
Methylobacterium extorquens DM4	Yes	1	1	0.72
Methylotenera mobilis JLW8	Yes	1	1	0.52
Methylovorus sp. SIP3-4	Yes	1	1	0.6
Mycobacterium tuberculosis KZN 1435	Yes	1	-1	-1
Neptuniibacter caesariensis	Yes	1	1	0.56
Nitrococcus mobilis Nb-231	Yes	1	1	0.79
Nitrosomonas sp. AL212	Yes	1	1	0.73
Oceanicaulis alexandrii HTCC2633	Yes	1	1	0.8
Parvularcula bermudensis HTCC2503	Yes	1	1	0.87
Pedobacter heparinus DSM 2366	Yes	1	-1	-0.05
Photobacterium angustum S14	Yes	1	1	0.67
Photobacterium profundum 3TCK	Yes	1	1	0.52
Photorhabdus asymbiotica	Yes	1	1	0.66
Planctomyces limnophilus DSM 3776	Yes	1	1	0.12
Polaribacter irgensii 23-P	Yes	1	1	0.05

Proteus penneri ATCC 35198	Yes	1	1	0.48
Providencia alcalifaciens DSM 30120	Yes	1	1	0.44
Providencia rustigianii DSM 4541	Yes	1	1	0.38
Providencia stuartii ATCC 25827	Yes	1	1	0.42
Pseudoalteromonas tunicata D2	Yes	1	1	0.88
Pseudomonas fluorescens SBW25	Yes	1	1	0.89
Psychroflexus torquis ATCC 700755	Yes	1	1	0.34
Psychromonas sp. CNPT3	Yes	1	1	0.34
Rhizobium sp. NGR234	Yes	1	1	0.63
Roseobacter sp. MED193	Yes	1	1	0.3
Roseovarius nubinhibens ISM	Yes	1	1	0.12
Salmonella enterica subsp. enterica serovar Kentucky str. CVM29188	Yes	1	1	0.95
Salmonella enterica subsp. enterica serovar Virchow str. SL491	Yes	1	1	1.02
Stigmatella aurantiaca DW4/3-1	Yes	1	1	0.76
Sulfitobacter sp. NAS-14.1	Yes	1	1	0.26
Sulfurihydrogenibium azorense Az-Fu1	Yes	1	1	0.57
Sulfurihydrogenibium yellowstonense SS-5	Yes	1	1	0.74
Sulfurospirillum deleyianum DSM 6946	Yes	1	1	0.55
Teredinibacter turnerae T7901	Yes	1	1	0.69
Thermanaerovibrio acidaminovorans DSM 6589	Yes	1	-1	-0.04
Thermococcus gammatolerans EJ3	Yes	1	1	0.82
Thermotoga naphthophila RKU-10	Yes	1	1	0.81
Thiomonas intermedia K12	Yes	1	1	0.27
Vibrio alginolyticus 12G01	Yes	1	1	0.91
Vibrio cholerae 12129(1)	Yes	1	1	0.82
Vibrio cholerae 623-39	Yes	1	1	0.9
Vibrio cholerae BX 330286	Yes	1	1	0.91
Vibrio cholerae M66-2	Yes	1	1	0.9
Vibrio cholerae MJ-1236	Yes	1	1	0.86
Vibrio cholerae MZO-2	Yes	1	1	0.81
Vibrio cholerae NCTC 8457	Yes	1	1	1
Vibrio cholerae O395	Yes	1	1	0.89
Vibrio cholerae RC9	Yes	1	1	0.95
Vibrio cholerae TM 11079-80	Yes	1	1	0.82
Vibrio cholerae TMA 21	Yes	1	1	0.82
Vibrio cholerae bv. albensis VL426	Yes	1	1	0.76
Vibrio harveyi HY01	Yes	1	1	1.18
Vibrio parahaemolyticus AQ3810	Yes	1	1	0.99
Vibrio sp. MED222	Yes	1	1	0.61
Vibrio splendidus 12B01	Yes	1	1	0.59
Yersinia aldovae ATCC 35236	Yes	1	1	0.71
Yersinia bercovieri ATCC 43970	Yes	1	1	0.81
Yersinia frederiksenii ATCC 33641	Yes	1	1	0.79
Yersinia intermedia ATCC 29909	Yes	1	1	0.84
Yersinia mollaretii ATCC 43969	Yes	1	1	0.88
Yersinia rohdei ATCC 43380	Yes	1	1	0.68
Acidobacterium capsulatum ATCC 51196	No	-1	-1	-0.17
Actinomyces odontolyticus ATCC 17982	No	-1	-1	-0.73
Alistipes putredinis DSM 17216	No	-1	-1	-0.35
Anaerococcus prevotii DSM 20548	No	-1	-1	-0.85
Anaerotruncus colihominis DSM 17241	No	-1	1	0.41
**Bacillus cereus 03BB102**	**No**	**-1**	**1**	**0.98**
Bacillus mycoides DSM 2048	No	-1	1	0.77
Bacillus mycoides Rock1-4	No	-1	1	0.73
Bacillus mycoides Rock3-17	No	-1	1	0.72
Bacillus pseudomycoides DSM 12442	No	-1	1	0.68
Bacteroides dorei DSM 17855	No	-1	-1	-0.66
Bacteroides eggerthii DSM 20697	No	-1	-1	-0.49
Bacteroides pectinophilus ATCC 43243	No	-1	1	0.38
Bacteroides plebeius DSM 17135	No	-1	-1	-0.35
Beutenbergia cavernae DSM 12333	No	-1	-1	-0.41
Bifidobacterium adolescentis L2-32	No	-1	-1	-0.86
Bifidobacterium angulatum DSM 20098	No	-1	-1	-0.74
Bifidobacterium animalis subsp. lactis Bl-04	No	-1	-1	-1.02
Bifidobacterium animalis subsp. lactis DSM 10140	No	-1	-1	-1.02

Bifidobacterium breve DSM 20213	No	-1	-1	-0.87
Brachybacterium faecium DSM 4810	No	-1	-1	-0.49
Bryantella formatexigens DSM 14469	No	-1	1	0.19
**Burkholderia glumae BGR1**	**No**	**-1**	**1**	**0.63**
Burkholderia mallei GB8 horse 4	No	-1	-1	-0.2
Burkholderia mallei PRL-20	No	-1	-1	-0.27
Clostridium cellulovorans 743B	No	-1	1	0.75
Clostridium hiranonis DSM 13275	No	-1	-1	-0.25
Clostridium hylemonae DSM 15053	No	-1	1	0.16
Clostridium spiroforme DSM 1552	No	-1	-1	-0.57
Collinsella stercoris DSM 13279	No	-1	-1	-0.74
Coprococcus comes ATCC 27758	No	-1	-1	-0.02
Coprococcus eutactus ATCC 27759	No	-1	1	0
Corynebacterium aurimucosum ATCC 700975	No	-1	-1	-0.89
Corynebacterium tuberculostearicum SK141	Non-motile	-1	-1	-0.72
Croceibacter atlanticus HTCC2559	No	-1	1	0.23
Cryptobacterium curtum DSM 15641	No	-1	-1	-0.68
Deinococcus deserti VCD115	No	-1	-1	-0.2
Dyadobacter fermentans DSM 18053	No	-1	1	0.07
Escherichia albertii TW07627	No	-1	1	0.87
Faecalibacterium prausnitzii A2-165	No	-1	-1	-0.08
Faecalibacterium prausnitzii M21/2	No	-1	-1	-0.11
Fibrobacter succinogenes subsp. succinogenes S85	No	-1	-1	-0.04
Francisella novicida FTE	No	-1	-1	-0.89
Geodermatophilus obscurus DSM 43160	No	-1	-1	-0.27
Gordonia bronchialis DSM 43247	No	-1	-1	-0.74
Kangiella koreensis DSM 16069	No	-1	1	0.38
Kordia algicida OT-1	No	-1	1	0.38
Kytococcus sedentarius DSM 20547	No	-1	-1	-0.53
Leptotrichia buccalis DSM 1135	No	-1	-1	-0.29
Macrococcus caseolyticus JCSC5402	No	-1	-1	-0.51
Meiothermus ruber DSM 1279	No	-1	-1	-0.05
Meiothermus silvanus DSM 9946	No	-1	-1	-0.4
Methanobrevibacter smithii DSM 2375	No	-1	-1	-0.8
Mycobacterium bovis BCG str. Tokyo 172	No	-1	-1	-0.99
Mycoplasma conjunctivae	No	-1	-1	-0.56
Mycoplasma mycoides subsp. capri str. GM12	No	-1	-1	-0.79
Neisseria sicca ATCC 29256	No	-1	-1	-0.29
Oceanicola batsensis HTCC2597	No	-1	1	0.16
Oceanicola granulosus HTCC2516	No	-1	1	0.47
Prevotella copri DSM 18205	No	-1	-1	-0.12
Rhodococcus opacus B4	No	-1	-1	-0.66
Ruminococcus lactaris ATCC 29176	No	-1	-1	-0.04
Ruminococcus obeum ATCC 29174	No	-1	-1	-0.13
Ruminococcus torques ATCC 27756	No	-1	-1	-0.25
Sebaldella termitidis ATCC 33386	No	-1	1	0.03
Slackia heliotrinireducens DSM 20476	No	-1	-1	-0.31
Stackebrandtia nassauensis DSM 44728	No	-1	-1	-0.53
Staphylococcus aureus subsp. aureus USA300_TCH959	No	-1	-1	-0.87
Streptococcus dysgalactiae subsp. equisimilis GGS_124	No	-1	-1	-0.84
Streptococcus infantarius subsp. infantarius ATCC BAA-102	No	-1	-1	-0.91
Streptococcus mutans NN2025	No	-1	-1	-0.87
Streptococcus pneumoniae 70585	No	-1	-1	-1.02
Streptococcus pneumoniae JJA	No	-1	-1	-1.04
Streptococcus pneumoniae P1031	No	-1	-1	-0.96
Streptococcus pneumoniae Taiwan19F-14	No	-1	-1	-0.96
Streptococcus suis 89/1591	No	-1	-1	-0.71
Streptococcus suis BM407	No	-1	-1	-0.87
Streptococcus suis P1/7	No	-1	-1	-0.91
Streptococcus suis SC84	No	-1	-1	-0.89
Subdoligranulum variabile DSM 15176	No	-1	-1	-0.32
Sulfolobus islandicus M.16.27	No	-1	-1	-0.48
Sulfolobus islandicus M.16.4	No	-1	-1	-0.67
Sulfolobus islandicus Y.N.15.51	No	-1	-1	-0.61
Sulfolobus solfataricus 98/2	No	-1	-1	-0.69

Tolumonas auensis DSM 9187	No	-1	-1	-0.07
Tsukamurella paurometabola DSM 20162	No	-1	-1	-0.74
Veillonella parvula DSM 2008	No	-1	-1	-0.49
Xylanimonas cellulosilytica DSM 15894	No	-1	-1	-0.55
Zymomonas mobilis subsp. mobilis ATCC 10988	No	-1	1	0.36

**Oxygen Requirement**	NCBI		prediction	
organism	annotation	label	label	score
Acetobacter pasteurianus IFO 3283-01	Aerobic	1	1	1.1
Acetobacter pasteurianus IFO 3283-01-42C	Aerobic	1	1	1.06
Acetobacter pasteurianus IFO 3283-03	Aerobic	1	1	1.1
Acetobacter pasteurianus IFO 3283-07	Aerobic	1	1	1.1
Acetobacter pasteurianus IFO 3283-22	Aerobic	1	1	1.1
Acetobacter pasteurianus IFO 3283-26	Aerobic	1	1	1.1
Acetobacter pasteurianus IFO 3283-32	Aerobic	1	1	1.1
Acidobacterium capsulatum ATCC 51196	Aerobic	1	1	0.37
Acidovorax delafieldii 2AN	Aerobic	1	1	0.89
Asticcacaulis excentricus CB 48	Aerobic	1	1	0.87
Azotobacter vinelandii DJ	Aerobic	1	1	0.76
Bacillus cereus 03BB102	Aerobic	1	1	1
Bacillus cereus 172560W	Aerobic	1	1	0.95
Bacillus cereus 95/8201	Aerobic	1	1	0.99
Bacillus cereus AH1271	Aerobic	1	1	0.98
Bacillus cereus AH1272	Aerobic	1	1	0.93
Bacillus cereus AH1273	Aerobic	1	1	0.91
Bacillus cereus AH603	Aerobic	1	1	0.88
Bacillus cereus AH621	Aerobic	1	1	0.91
Bacillus cereus AH676	Aerobic	1	1	0.96
Bacillus cereus ATCC 10876	Aerobic	1	1	0.96
Bacillus cereus ATCC 4342	Aerobic	1	1	1.04
Bacillus cereus BDRD-Cer4	Aerobic	1	1	0.91
Bacillus cereus BDRD-ST196	Aerobic	1	1	0.9
Bacillus cereus BDRD-ST24	Aerobic	1	1	1
Bacillus cereus BDRD-ST26	Aerobic	1	1	0.89
Bacillus cereus F65185	Aerobic	1	1	0.99
Bacillus cereus H3081.97	Aerobic	1	1	0.97
Bacillus cereus MM3	Aerobic	1	1	0.96
Bacillus cereus R309803	Aerobic	1	1	0.9
Bacillus cereus Rock3-28	Aerobic	1	1	0.94
Bacillus cereus Rock3-29	Aerobic	1	1	1.02
Bacillus cereus Rock3-44	Aerobic	1	1	0.74
Bacillus cereus Rock4-18	Aerobic	1	1	0.88
Bacillus cereus m1293	Aerobic	1	1	0.97
Bacillus cereus m1550	Aerobic	1	1	0.97
Bacillus mycoides Rock1-4	Aerobic	1	1	0.89
Bacillus mycoides Rock3-17	Aerobic	1	1	0.83
Bacillus pseudomycoides DSM 12442	Aerobic	1	1	0.81
Bacillus thuringiensis IBL 200	Aerobic	1	1	0.98
Bacillus thuringiensis IBL 4222	Aerobic	1	1	0.96
Bacillus thuringiensis serovar andalousiensis BGSC 4AW1	Aerobic	1	1	1.01
Bacillus thuringiensis serovar huazhongensis BGSC 4BD1	Aerobic	1	1	0.98
Bacillus thuringiensis serovar kurstaki str. T03a001	Aerobic	1	1	0.92
Bacillus thuringiensis serovar pakistani str. T13001	Aerobic	1	1	0.89
Bacillus thuringiensis serovar pondicheriensis BGSC 4BA1	Aerobic	1	1	0.98
Bacillus thuringiensis serovar pulsiensis BGSC 4CC1	Aerobic	1	1	0.95
Bacillus thuringiensis serovar sotto str. T04001	Aerobic	1	1	0.98
Bacillus thuringiensis serovar thuringiensis str. T01001	Aerobic	1	1	0.98
Bacillus thuringiensis serovar tochigiensis BGSC 4Y1	Aerobic	1	1	1
Bartonella grahamii as4aup	Aerobic	1	1	1.06
Bermanella marisrubri	Aerobic	1	1	0.88
Beutenbergia cavernae DSM 12333	Aerobic	1	1	0.72
Brachybacterium faecium DSM 4810	Aerobic	1	1	0.51
Brevibacillus brevis NBRC 100599	Aerobic	1	1	0.72
Brucella ceti str. Cudo	Aerobic	1	1	1.1
Burkholderia glumae BGR1	Aerobic	1	1	0.95

Burkholderia mallei PRL-20	Aerobic	1	1	1.05
Burkholderia pseudomallei MSHR346	Aerobic	1	1	1.04
Candidatus Pelagibacter ubique HTCC1002	Aerobic	1	1	1.08
Catenulispora acidiphila DSM 44928	Aerobic	1	1	0.84
Chitinophaga pinensis DSM 2588	Aerobic	1	1	0.67
Croceibacter atlanticus HTCC2559	Aerobic	1	1	1.17
Deinococcus deserti VCD115	Aerobic	1	1	0.91
**Dethiosulfovibrio peptidovorans DSM 11002**	**Aerobic**	**1**	-1	**-0.78**
Dyadobacter fermentans DSM 18053	Aerobic	1	1	0.69
Erythrobacter sp. NAP1	Aerobic	1	1	0.79
Francisella novicida FTE	Aerobic	1	1	1.01
Fulvimarina pelagi HTCC2506	Aerobic	1	1	0.89
Gemmatimonas aurantiaca T-27	Aerobic	1	1	0.61
Geobacillus sp. Y4.1MC1	Aerobic	1	1	0.68
Geodermatophilus obscurus DSM 43160	Aerobic	1	1	1.04
Gordonia bronchialis DSM 43247	Aerobic	1	1	0.95
Haliangium ochraceum DSM 14365	Aerobic	1	1	0.74
Halogeometricum borinquense DSM 11551	Aerobic	1	1	0.62
Halorhabdus utahensis DSM 12940	Aerobic	1	1	0.39
Helicobacter pylori B38	Aerobic	1	1	0.89
Hirschia baltica ATCC 49814	Aerobic	1	1	0.8
Hyphomicrobium denitrificans ATCC 51888	Aerobic	1	1	0.87
Idiomarina baltica OS145	Aerobic	1	1	0.69
Kordia algicida OT-1	Aerobic	1	1	1.02
Kribbella flavida DSM 17836	Aerobic	1	1	0.83
Kytococcus sedentarius DSM 20547	Aerobic	1	1	0.92
Loktanella vestfoldensis SKA53	Aerobic	1	1	0.24
Marinomonas sp. MED121	Aerobic	1	1	0.76
Meiothermus ruber DSM 1279	Aerobic	1	1	0.95
Meiothermus silvanus DSM 9946	Aerobic	1	1	0.95
Methylobacterium extorquens DM4	Aerobic	1	1	0.95
Methylotenera mobilis JLW8	Aerobic	1	1	0.66
Methylovorus sp. SIP3-4	Aerobic	1	1	0.9
Mycobacterium bovis BCG str. Tokyo 172	Aerobic	1	1	1.02
Mycobacterium tuberculosis KZN 1435	Aerobic	1	1	1.01
Neisseria sicca ATCC 29256	Aerobic	1	1	0.96
Neptuniibacter caesariensis	Aerobic	1	1	0.64
Nitrococcus mobilis Nb-231	Aerobic	1	1	0.75
Oceanicaulis alexandrii HTCC2633	Aerobic	1	1	1.03
Oceanicola batsensis HTCC2597	Aerobic	1	1	1.01
Oceanicola granulosus HTCC2516	Aerobic	1	1	0.71
Parvularcula bermudensis HTCC2503	Aerobic	1	1	1.08
Pedobacter heparinus DSM 2366	Aerobic	1	1	0.48
Planctomyces limnophilus DSM 3776	Aerobic	1	1	0.46
Polaribacter irgensii 23-P	Aerobic	1	1	0.89
Pseudomonas fluorescens SBW25	Aerobic	1	1	1.05
Psychroflexus torquis ATCC 700755	Aerobic	1	1	1.04
Ralstonia solanacearum IPO1609	Aerobic	1	1	1.1
Rhizobium sp. NGR234	Aerobic	1	1	1.16
Rhodococcus opacus B4	Aerobic	1	1	1.17
Rhodothermus marinus DSM 4252	Aerobic	1	1	0.62
Rickettsia africae ESF-5	Aerobic	1	1	1.04
Rickettsia peacockii str. Rustic	Aerobic	1	1	0.98
Robiginitalea biformata HTCC2501	Aerobic	1	1	0.83
Roseobacter sp. MED193	Aerobic	1	1	0.9
Roseovarius nubinhibens ISM	Aerobic	1	1	0.68
Roseovarius sp. 217	Aerobic	1	1	0.61
Roseovarius sp. HTCC2601	Aerobic	1	1	0.87
Sphingomonas sp. SKA58	Aerobic	1	1	1.08
Stackebrandtia nassauensis DSM 44728	Aerobic	1	1	0.88
Stigmatella aurantiaca DW4/3-1	Aerobic	1	1	0.73
Streptosporangium roseum DSM 43021	Aerobic	1	1	1
Sulfitobacter sp. NAS-14.1	Aerobic	1	1	0.9
Sulfolobus islandicus L.S.2.15	Aerobic	1	1	0.82
Sulfolobus islandicus M.14.25	Aerobic	1	1	0.73

Sulfolobus islandicus M.16.27	Aerobic	1	1	0.59
Sulfolobus islandicus M.16.4	Aerobic	1	1	0.78
Sulfolobus solfataricus 98/2	Aerobic	1	1	0.97
Sulfurihydrogenibium yellowstonense SS-5	Aerobic	1	1	0.06
Teredinibacter turnerae T7901	Aerobic	1	1	0.69
Thermomonospora curvata DSM 43183	Aerobic	1	1	0.73
Thiomonas intermedia K12	Aerobic	1	1	0.86
Xylanimonas cellulosilytica DSM 15894	Aerobic	1	1	0.64
**Acidimicrobium ferrooxidans DSM 10331**	**Anaerobic**	**-1**	**1**	**0.78**
Alistipes putredinis DSM 17216	Anaerobic	-1	-1	-0.55
Anaerococcus prevotii DSM 20548	Anaerobic	-1	-1	-0.75
Anaerofustis stercorihominis DSM 17244	Anaerobic	-1	-1	-1.3
Anaerostipes caccae DSM 14662	Anaerobic	-1	-1	-1.18
Anaerotruncus colihominis DSM 17241	Anaerobic	-1	-1	-0.55
Bacteroides capillosus ATCC 29799	Anaerobic	-1	-1	-1.07
Bacteroides coprocola DSM 17136	Anaerobic	-1	-1	-0.67
Bacteroides dorei DSM 17855	Anaerobic	-1	-1	-0.85
Bacteroides eggerthii DSM 20697	Anaerobic	-1	-1	-0.91
Bacteroides intestinalis DSM 17393	Anaerobic	-1	-1	-1.09
Bacteroides ovatus ATCC 8483	Anaerobic	-1	-1	-0.92
Bacteroides pectinophilus ATCC 43243	Anaerobic	-1	-1	-0.84
Bacteroides plebeius DSM 17135	Anaerobic	-1	-1	-0.97
Bacteroides stercoris ATCC 43183	Anaerobic	-1	-1	-0.89
Bacteroides uniformis ATCC 8492	Anaerobic	-1	-1	-1.07
Bifidobacterium adolescentis L2-32	Anaerobic	-1	-1	-0.9
Bifidobacterium angulatum DSM 20098	Anaerobic	-1	-1	-1.02
Bifidobacterium animalis subsp. lactis Bl-04	Anaerobic	-1	-1	-0.99
Bifidobacterium animalis subsp. lactis DSM 10140	Anaerobic	-1	-1	-1
Bifidobacterium breve DSM 20213	Anaerobic	-1	-1	-0.86
Bifidobacterium dentium ATCC 27678	Anaerobic	-1	-1	-0.68
Blautia hydrogenotrophica DSM 10507	Anaerobic	-1	-1	-0.64
Bryantella formatexigens DSM 14469	Anaerobic	-1	-1	-0.81
Citrobacter youngae ATCC 29220	Anaerobic	-1	-1	-0.78
Clostridium asparagiforme DSM 15981	Anaerobic	-1	-1	-0.5
Clostridium bartlettii DSM 16795	Anaerobic	-1	-1	-1.08
Clostridium bolteae ATCC BAA-613	Anaerobic	-1	-1	-0.45
Clostridium botulinum A2 str. Kyoto	Anaerobic	-1	-1	-0.99
Clostridium botulinum Ba4 str. 657	Anaerobic	-1	-1	-0.96
Clostridium botulinum D str. 1873	Anaerobic	-1	-1	-1.07
Clostridium cellulovorans 743B	Anaerobic	-1	-1	-0.63
Clostridium hiranonis DSM 13275	Anaerobic	-1	-1	-1.18
Clostridium hylemonae DSM 15053	Anaerobic	-1	-1	-0.65
Clostridium kluyveri NBRC 12016	Anaerobic	-1	-1	-1.02
Clostridium leptum DSM 753	Anaerobic	-1	-1	-1
Clostridium nexile DSM 1787	Anaerobic	-1	-1	-0.76
Clostridium ramosum DSM 1402	Anaerobic	-1	-1	-0.58
Clostridium scindens ATCC 35704	Anaerobic	-1	-1	-0.81
Clostridium sp. L2-50	Anaerobic	-1	-1	-0.97
Clostridium sp. M62/1	Anaerobic	-1	-1	-0.85
Clostridium sp. SS2/1	Anaerobic	-1	-1	-0.79
Clostridium spiroforme DSM 1552	Anaerobic	-1	-1	-1.15
Collinsella stercoris DSM 13279	Anaerobic	-1	-1	-1.08
Coprococcus comes ATCC 27758	Anaerobic	-1	-1	-1
Coprococcus eutactus ATCC 27759	Anaerobic	-1	-1	-0.82
Cryptobacterium curtum DSM 15641	Anaerobic	-1	-1	-0.56
Denitrovibrio acetiphilus DSM 12809	Anaerobic	-1	-1	-0.63
Desulfomicrobium baculatum DSM 4028	Anaerobic	-1	-1	-0.85
Desulfovibrio piger ATCC 29098	Anaerobic	-1	-1	-0.86
Dorea formicigenerans ATCC 27755	Anaerobic	-1	-1	-0.9
Dorea longicatena DSM 13814	Anaerobic	-1	-1	-1.25
Eikenella corrodens ATCC 23834	Anaerobic	-1	1	0.65
Eubacterium biforme DSM 3989	Anaerobic	-1	-1	-0.76
Eubacterium dolichum DSM 3991	Anaerobic	-1	-1	-0.7
Eubacterium eligens ATCC 27750	Anaerobic	-1	-1	-0.8
Eubacterium rectale ATCC 33656	Anaerobic	-1	-1	-0.99

Eubacterium siraeum DSM 15702	Anaerobic	-1	-1	-1.02
Eubacterium ventriosum ATCC 27560	Anaerobic	-1	-1	-0.88
Faecalibacterium prausnitzii A2-165	Anaerobic	-1	-1	-0.91
Faecalibacterium prausnitzii M21/2	Anaerobic	-1	-1	-0.94
Fibrobacter succinogenes subsp. succinogenes S85	Anaerobic	-1	-1	-0.46
Holdemania filiformis DSM 12042	Anaerobic	-1	-1	-0.63
Laribacter hongkongensis HLHK9	Anaerobic	-1	1	0.26
Leptotrichia buccalis DSM 1135	Anaerobic	-1	-1	-0.52
Methanobrevibacter smithii DSM 2375	Anaerobic	-1	-1	-1.05
Methanocaldococcus fervens AG86	Anaerobic	-1	-1	-1.03
Mycoplasma mycoides subsp. capri str. GM12	Anaerobic	-1	1	0.35
Parabacteroides johnsonii DSM 18315	Anaerobic	-1	-1	-1
Parabacteroides merdae ATCC 43184	Anaerobic	-1	-1	-1.12
Parvimonas micra ATCC 33270	Anaerobic	-1	-1	-0.72
Prevotella copri DSM 18205	Anaerobic	-1	-1	-0.83
Proteus penneri ATCC 35198	Anaerobic	-1	-1	-0.57
Ruminococcus gnavus ATCC 29149	Anaerobic	-1	-1	-0.95
Ruminococcus lactaris ATCC 29176	Anaerobic	-1	-1	-1.35
Ruminococcus obeum ATCC 29174	Anaerobic	-1	-1	-1.24
Ruminococcus torques ATCC 27756	Anaerobic	-1	-1	-1.13
Sebaldella termitidis ATCC 33386	Anaerobic	-1	-1	-0.65
Slackia heliotrinireducens DSM 20476	Anaerobic	-1	-1	-0.95
Subdoligranulum variabile DSM 15176	Anaerobic	-1	-1	-1.12
Thermanaerovibrio acidaminovorans DSM 6589	Anaerobic	-1	-1	-0.85
Thermoanaerobacter ethanolicus CCSD1	Anaerobic	-1	-1	-1.06
Thermoanaerobacter sp. X513	Anaerobic	-1	-1	-1
Thermoanaerobacter sp. X561	Anaerobic	-1	-1	-0.99
Thermoanaerobacterium thermosaccharolyticum DSM 571	Anaerobic	-1	-1	-1.02
Thermococcus gammatolerans EJ3	Anaerobic	-1	-1	-0.93
Thermotoga naphthophila RKU-10	Anaerobic	-1	-1	-1.05
Veillonella dispar ATCC 17748	Anaerobic	-1	-1	-0.74
Veillonella parvula DSM 2008	Anaerobic	-1	-1	-0.73
Vibrio cholerae TMA 21	Anaerobic	-1	-1	-0.59
Vibrio cholerae bv. albensis VL426	Anaerobic	-1	-1	-0.62
Zymomonas mobilis subsp. mobilis ATCC 10988	Anaerobic	-1	1	0.47
