# Supplementary material for: Predicting phenotypic traits of prokaryotes from protein domain frequencies
Source: BMC Bioinformatics. 2010 Sep 24;11:481. doi: 10.1186/1471-2105-11-481 (PMC2955703; doi:10.1186/1471-2105-11-481)
Supplement: Additional file 6 — Histograms of phenotype-specific phylogenetic distribution of example organisms. The file "histoGroups.pdf" contains phylum-level histogram plots of the phenotype-specific number of positive and negative examples. [file 1471-2105-11-481-S6.PDF]

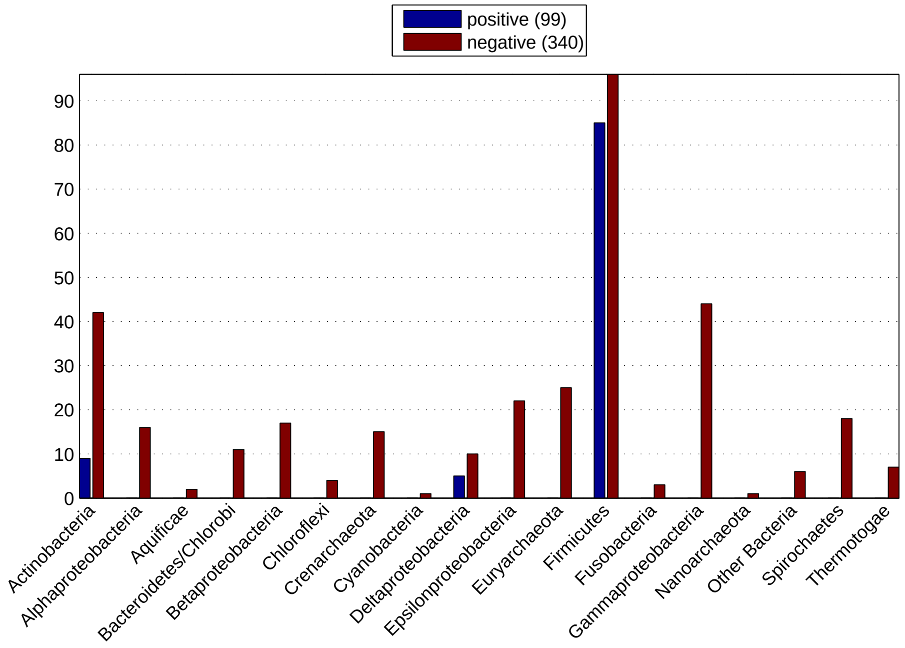

Figure 1: Histogram plot of the number of positive and negative examples for phenotype category "Endospores"

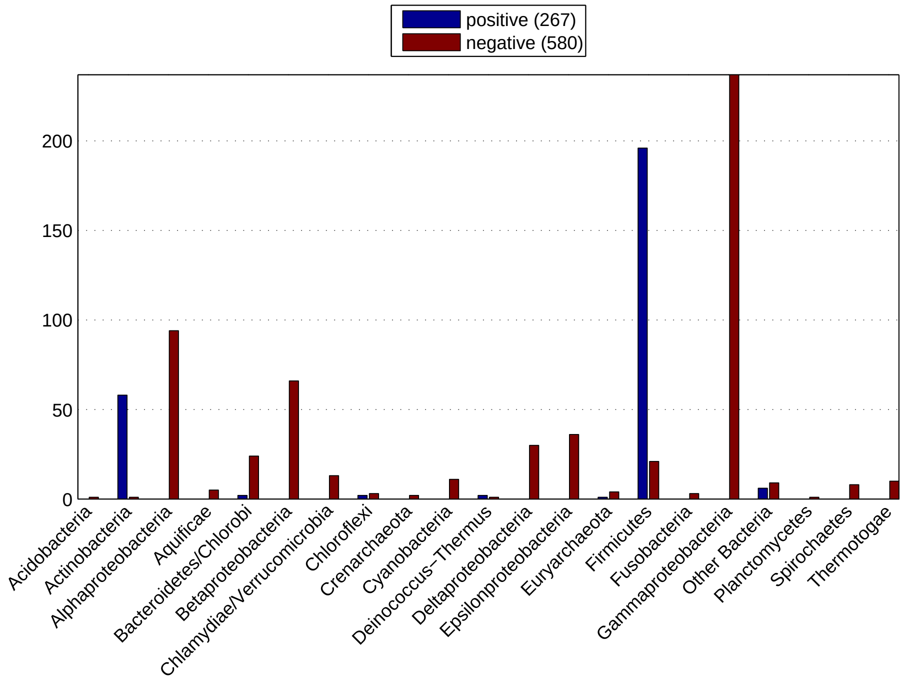

Figure 2: Histogram plot of the number of positive and negative examples for phenotype category "Gram Stain"

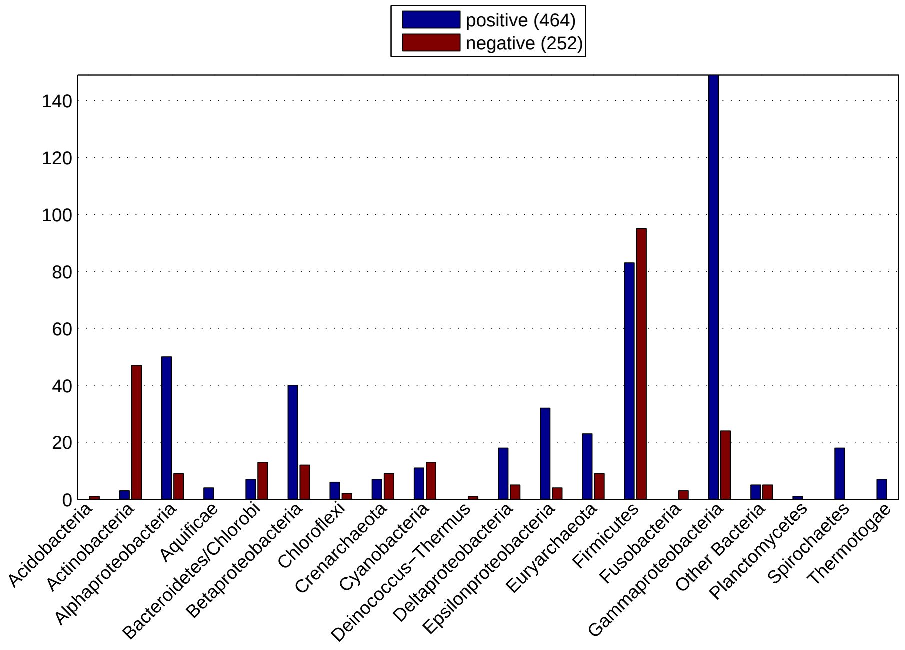

Figure 3: Histogram plot of the number of positive and negative examples for phenotype category "Motility"

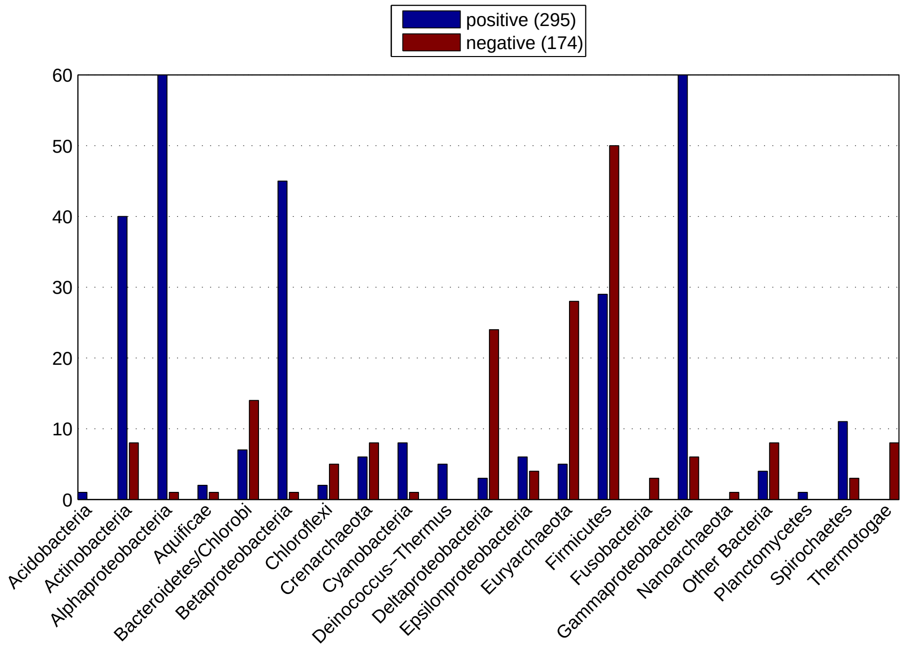

Figure 4: Histogram plot of the number of positive and negative examples for phenotype category "Oxygen Requirement"
